# Supplementary material for: Diabetes as a risk factor for the onset of frozen shoulder: a systematic review and meta-analysis
Source: BMJ Open. 2023 Jan 4;13(1):e062377. doi: 10.1136/bmjopen-2022-062377 (PMC9815013; doi:10.1136/bmjopen-2022-062377)
Supplement: Supplementary data [file bmjopen-2022-062377supp001.pdf]

Appendix A

The following searches were originally conducted in December 2018 and updated in June 2021.

MEDLINE

Interface: OVID.

1. ((shoulder* or glenohumer* or subacromi* or acromi* or rotator cuff) adj3 (instability or bursitis or frozen or impinge* or tendonitis or tendinitis or pain* or osteoarthr* or periarthriti* or peri arthriti* or arthralgia)).ti,ab,kw.

2. Shoulder Impingement Syndrome/

3. exp Bursitis/

4. Rotator Cuff/

5. adhesive capsuliti*.ti,ab,kw.

6. Shoulder Pain/

7. or/1-6

8. exp Pain/

9. pain*.ti,ab,kw.

10. Arthralgia/

11. arthralgia.ti,ab,kw.

12. or/8-11

13. Shoulder/

14. Shoulder joint/

15. Acromioclavicular Joint/

16. (shoulder* or glenohumer* or subacromi* or acromi* or rotator cuff).ti,ab,kw.

17. or/13-16

18. 12 and 17

19. 7 or 18

20. exp Diabetes Mellitus/

21. diabet*.ti,ab,kw.

22. (DMi or DM i).ti,ab,kw.

23. (DM1 or DM 1).ti,ab,kw.

24. (DM2 or DM 2).ti,ab,kw.

25. (DMii or DM ii).ti,ab,kw.

26. (DM adj2 type).ti,ab,kw.

27. or/20-26

28. 19 and 27

29. exp animals/ not humans/

30. 28 not 29

EMBASE

Interface: OVID.

1. ((shoulder* or glenohumer* or subacromi* or acromi* or rotator cuff) adj3 (instability or bursitis or frozen or impinge* or tendonitis or tendinitis or pain* or osteoarthr* or periarthriti* or peri arthriti* or arthralgia)).ti,ab,kw.

2. exp shoulder impingement syndrome/

3. exp bursitis/

4. exp rotator cuff/

5. exp humeroscapular periarthritis/

6. adhesive capsuliti*.ti,ab,kw.

7. exp shoulder pain/

8. or/1-7

9. exp pain/

10. pain*.ti,ab,kw.

11. exp arthralgia/

12. arthralgia.ti,ab,kw.

13. or/9-12

14. exp shoulder/

15. Acromioclavicular Joint/

16. (shoulder* or glenohumer* or subacromi* or acromi* or rotator cuff).ti,ab,kw.

17. or/14-16

18. 13 and 17

19. 8 or 18

20. exp Diabetes Mellitus/

21. diabet*.ti,ab,kw.

22. (DMi or DM i).ti,ab,kw.

23. (DM1 or DM1).ti,ab,kw.

24. (DM2 or DM 2).ti,ab,kw.

25. (DMii or DM ii).ti,ab,kw.

26. (DM adj2 type).ti,ab,kw.

27. or/20-26

28. 19 and 27

29. exp animals/ not humans/

30. 28 not 29

31. limit 30 to embase

AMED

Interface: OVID.

1. ((shoulder* or glenohumer* or subacromi* or acromi* or rotator cuff) adj3 (instability or bursitis or frozen or impinge* or tendonitis or tendinitis or pain* or osteoarthr* or periarthriti* or peri arthriti* or arthralgia)).ti,ab.

2. exp Shoulder impingement syndrome/

3. exp Bursitis/

4. exp Rotator cuff/

5. adhesive capsuliti*.ti,ab.

6. exp shoulder pain/

7. or/1-6

8. exp Pain/

9. pain*.ti,ab.

10. exp Arthralgia/

11. arthralgia.ti,ab.

12. or/8-11

13. shoulder/

14. (shoulder* or glenohumer* or subacromi* or acromi* or rotator cuff).ti,ab.

15. or/13-14

16. 12 and 15

17. 7 or 16

18. exp Diabetes mellitus/

19. diabet*.ti,ab.

20. (DMi or DM i).ti,ab.

21. (DM1 or DM 1).ti,ab.

22. (DM2 or DM 2).ti,ab.

23. (DMii or DM ii).ti,ab.

24. (DM adj2 type).ti,ab.

25. or/18-24

26. 17 and 25

27. exp animals/ not humans/

28. 26 not 27

PsycINFO

Interface: OVID.

1. ((shoulder* or glenohumer* or subacromi* or acromi* or rotator cuff) adj3 (instability or bursitis or frozen or impinge* or tendonitis or tendinitis or pain* or osteoarthr* or periarthriti* or peri arthriti* or arthralgia)).ti,ab.

2. Shoulder Impingement Syndrome.ti,ab.

3. bursitis.ti,ab.

4. rotator cuff.ti,ab.

5. adhesive capsuliti*.ti,ab.

6. shoulder pain.ti,ab.

7. or/1-6

8. exp PAIN/

9. pain*.ti,ab.

10. arthralgia.ti,ab.

11. or/8-10

12. *"shoulder (anatomy)"/

13. shoulder*.ti,ab.

14. shoulder joint.ti,ab.

15. acromi*.ti,ab.

16. glenohumer*.ti,ab.

17. subacromi*.ti,ab.

18. or/12-17

19. 11 and 18

20. 7 or 19

21. exp DIABETES MELLITUS/

22. diabet*.ti,ab.

23. (DMi or DM i).ti,ab.

24. (DM1 or DM 1).ti,ab.

25. (DM2 or DM 2).ti,ab.
26. (DMii or DM ii).ti,ab.

27. (DM adj2 type).ti,ab.

28. or/21-27

29. 20 and 28

Web of Science

Science Citation Index Expanded and the Science Conference Proceedings Citation Index.

((

TS=(Shoulder* NEAR/3 instability) OR TS=(Shoulder* NEAR/3 bursitis) OR TS=(Shoulder*

NEAR/3 frozen) OR TS=(Shoulder* NEAR/3 impinge*) OR TS=(Shoulder* NEAR/3 tendonitis) OR TS=(Shoulder* NEAR/3 tendinitis) OR TS=(Shoulder* NEAR/3 pain) OR TS=(Shoulder*

NEAR/3 osteoarthr*) OR TS=(Shoulder* NEAR/3 periarthriti*) OR TS=(Shoulder* NEAR/3

“peri arthriti*”) OR TS=(Shoulder* NEAR/3 arthralgia)

OR

TS=(glenohumer* NEAR/3 instability) OR TS=(glenohumer* NEAR/3 bursitis) OR TS=(glenohumer*

NEAR/3 frozen) OR TS=(glenohumer* NEAR/3 impinge*) OR TS=(glenohumer* NEAR/3

tendonitis) OR TS=(glenohumer* NEAR/3 tendinitis) OR TS=(glenohumer* NEAR/3 pain)

OR TS=(glenohumer* NEAR/3 osteoarthr*) OR TS=(glenohumer* NEAR/3 periarthriti*) OR

TS=(glenohumer* NEAR/3 “peri arthriti*”) OR TS=(glenohumer* NEAR/3 arthralgia)

OR

TS=(subacromi* NEAR/3 instability) OR TS=(subacromi* NEAR/3 bursitis) OR TS=(subacromi*

NEAR/3 frozen) OR TS=(subacromi* NEAR/3 impinge*) OR TS=(subacromi* NEAR/3 tendonitis) OR TS=(subacromi* NEAR/3 tendinitis) OR TS=(subacromi* NEAR/3 pain) OR TS=(subacromi*

NEAR/3 osteoarthr*) OR TS=(subacromi* NEAR/3 periarthriti*) OR TS=(subacromi* NEAR/3

“peri arthriti*”) OR TS=(subacromi* NEAR/3 arthralgia)

OR

TS=(acromi* NEAR/3 instability) OR TS=(acromi* NEAR/3 bursitis) OR TS=(acromi* NEAR/3

frozen) OR TS=(acromi* NEAR/3 impinge*) OR TS=(acromi* NEAR/3 tendonitis) OR TS=(acromi*

NEAR/3 tendinitis) OR TS=(acromi* NEAR/3 pain) OR TS=(acromi* NEAR/3 osteoarthr*)

OR TS=(acromi* NEAR/3 periarthriti*) OR TS=(acromi* NEAR/3 “peri arthriti*”) OR TS=(acromi*

NEAR/3 arthralgia)

OR

TS=(“rotator cuff” NEAR/3 instability) OR TS=(“rotator cuff” NEAR/3 bursitis) OR TS=(“rotator cuff” NEAR/3 frozen) OR TS=(“rotator cuff” NEAR/3 impinge*) OR TS=(“rotator cuff” NEAR/3

tendonitis) OR TS=(“rotator cuff” NEAR/3 tendinitis) OR TS=(“rotator cuff” NEAR/3 pain)

OR TS=(“rotator cuff” NEAR/3 osteoarthr*) OR TS=(“rotator cuff” NEAR/3 periarthriti*) OR

TS=(“rotator cuff” NEAR/3 “peri arthriti*”) OR TS=(“rotator cuff” NEAR/3 arthralgia)

OR

TS=(“Rotator cuff”)

OR

TS=(“Adhesive capsuliti*”)

)

OR

TS=(arthralgia NEAR/3 shoulder* or arthralgia NEAR/3 glenohumer* or arthralgia NEAR/3

subacromi* or arthralgia NEAR/3 acromi* or arthralgia NEAR/3 “rotator cuff”)

OR TS=(pain* NEAR/3 shoulder* or pain* NEAR/3 glenohumer* or pain* NEAR/3 subacromi* or pain* NEAR/3 acromi* or pain* NEAR/3 “rotator cuff”)

)

And

TS=(diabet* or DM1 or “DM 1” or DM2 or “DM 2” or DMi or “DM i” or DMii or “DM ii” or

DM NEAR/2 type)

CINAHL

Interface: EBSCO. Filters: title or abstract

(

((shoulder* or glenohumer* or subacromi* or acromi* or “rotator cuff”) N3 (instability or bursitis or frozen or impinge* or tendonitis or tendinitis or pain* or osteoarthr* or periarthriti* or

“peri arthriti*” or arthralgia))

OR

(MH “Shoulder Impingement Syndrome”) OR (MH “Bursitis+”) OR (MH “Rotator Cuff+”) OR

(MH “Periarthritis”) OR (MH “Adhesive Capsulitis+”) OR (MH “Shoulder Pain”)

OR

((MH “Pain+”) or pain or (MH “Arthralgia+”) or arthralgia) and ((MH “Shoulder”) or (MH

“Acromioclavicular Joint”) or shoulder* or glenohumer* or subacromi* or acromi* or “rotator

cuff”)

)

AND

((MH “Diabetes Mellitus+”) or diabet* or (DMi or “DM i”) or (DM1 or “DM 1”) or (DMii or “DM ii”) or (DM2 or “DM 2”) or (DM N2 type))

Epistemonikos

Filters: title or abstract. Primary study. Not an RCT.

((“frozen shoulder” or “shoulder impinge*” or “shoulder bursitis” or “shoulder tendonitis” or “shoulder tendinitis” or “shoulder pain” or “pain in the shoulder” or “painful shoulder” or “shoulder osteoarthr*” or “shoulder joint arthr*” or “shoulder arthr”)

OR

(“glenohumeral impinge*” or “glenohumeral bursitis” or “glenohumeral tendonitis” or “glenohumeral tendinitis” or “glenohumeral pain” or “pain in the glenohumeral” or “glenohumeral osteoarthr*” or “glenohumeral arthr*” or “glenohumeral arthr”)

OR

(“subacromial impinge*” or “subacromial bursitis” or “subacromial tendonitis” or “subacromial tendinitis” or “subacromial pain” or “pain in the subacromial” or “subacromial osteoarthr*” or “subacromial arthr*” or “subacromial arthr”)

OR

“Rotator cuff”

OR

“periarthriti*”

OR

“peri arthriti*”

OR

“Adhesive capsuliti*”

)

AND

(diabet* or DM1 or DM2 or DMi or DMii or “type 1 DM” or “type 2 DM” or “type i DM” or “type ii DM”)

TRIP

("frozen shoulder" or "shoulder pain" or "periathriti*" or "peri arthriti*" or "adhesive capsuliti*" or "shoulder impingement" or "bursitis" or "rotator cuff") and "diabet*"

PEDro

Filters: body part = upper arm, shoulder or shoulder girdle

Title and abstract search: diabet*

Open Grey

Search 1: Diabet* and shoulder*

Search 2: Diabet* and glenohumer*

Search 3: Diabet* and subacromi*

Search 4: Diabet* and acromi*

Search 5: Diabet* and "rotator cuff*"

Search 6: Diabet* and bursitis

Search 7: Diabet* and periarthriti*

Search 8: Diabet* and "peri arthriti*"

Search 9: Diabet* and "adhesive capsuliti*"

Search 10: Diabet* and arthralgia

Grey literature report

Diabet*

Appendix B

Fig. B.1 Bar graph of QUIPS scores for each of the six bias domains: study participation, study attrition, diabetes/risk factor (RF) measurement, frozen shoulder/outcome measurement, study confounding, statistical analysis and reporting.

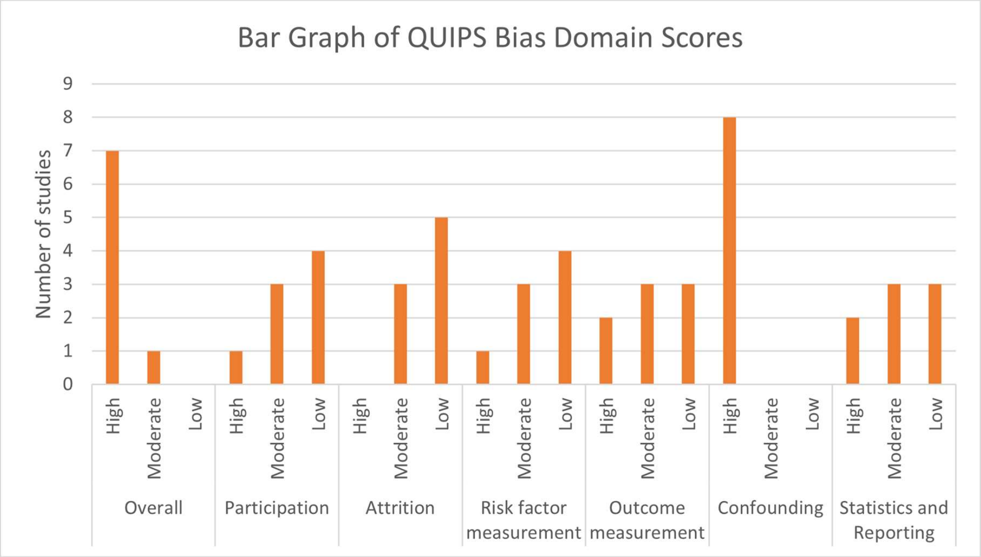

Appendix C

Table C.1 Raw data from each study.

**Case-Control Studies**				
Source	Number of cases	Number of controls	Number of cases with diabetes	Number of controls with diabetes
K. L. Boyle-Walker, et al., 1997 [32]	32	31	7	0
W. Li, et al., 2014 [33]	182	196	44	18
S-Y. Lee, et al., 2012 [34]	40	40	6	1
C. Milgrom, et al., 2008 [35]	126	98	37	11
K. Wang, et al., 2013 [36]	87	176	17	13
K. Kingston, et al., 2018 [37]	2190	2190	572	188
**Cohort studies**				
Source	Number of people with diabetes	Number of controls	Number of people with diabetes that developed frozen shoulder	Number of people without diabetes that developed frozen shoulder
Y-P. Huang, et al., 2013 [38]	78,827	236,481	946	2254
S-F. Lo, et al., 2013 [39]	5109	20,473	553	768
