# Supplementary material for: Diabetes as a risk factor for the onset of frozen shoulder: a systematic review and meta-analysis
Source: BMJ Open. 2023 Jan 4;13(1):e062377. doi: 10.1136/bmjopen-2022-062377 (PMC9815013; doi:10.1136/bmjopen-2022-062377)
Supplement: Author's manuscript [file bmjopen-2022-062377.draft_revisions.pdf]

BMJ Open is committed to open peer review. As part of this commitment we make the peer review history of every article we publish publicly available.

When an article is published we post the peer reviewers' comments and the authors' responses online. We also post the versions of the paper that were used during peer review. These are the versions that the peer review comments apply to.

The versions of the paper that follow are the versions that were submitted during the peer review process. They are not the versions of record or the final published versions. They should not be cited or distributed as the published version of this manuscript.

BMJ Open is an open access journal and the full, final, typeset and author-corrected version of record of the manuscript is available on our site with no access controls, subscription charges or pay-per-view fees (http://bmjopen.bmj.com).

If you have any questions on BMJ Open's open peer review process please email info.bmjopen@bmj.com

BMJ Open

Diabetes as a Risk Factor for the Onset of Frozen Shoulder: A Systematic Review and Meta-Analysis

Journal:	*BMJ Open*
Manuscript ID	bmjopen-2022-062377
Article Type:	Original research
Date Submitted by the Author:	28-Feb-2022
Complete List of Authors:	Dyer, Brett; Keele University, Primary Care Centre Versus Arthritis, School of Medicine<br>Rathod, Trishna; Keele University, Primary Care Centre Versus Arthritis, School of Medicine<br>Burton, Claire; Keele University, Primary Care Centre Versus Arthritis, School of Medicine<br>van der Windt, Danielle; Keele University, Primary Care Centre Versus Arthritis, School of Medicine<br>Bucknall, Milica; Keele University, Primary Care Centre Versus Arthritis, School of Medicine
Keywords:	DIABETES & ENDOCRINOLOGY, EPIDEMIOLOGY, PRIMARY CARE, RHEUMATOLOGY

SCHOLARONE™
Manuscripts

I, the Submitting Author has the right to grant and does grant on behalf of all authors of the Work (as defined in the below author licence), an exclusive licence and/or a non-exclusive licence for contributions from authors who are: i) UK Crown employees; ii) where BMJ has agreed a CC-BY licence shall apply, and/or iii) in accordance with the terms applicable for US Federal Government officers or employees acting as part of their official duties; on a worldwide, perpetual, irrevocable, royalty-free basis to BMJ Publishing Group Ltd ("BMJ") its licensees and where the relevant Journal is co-owned by BMJ to the co-owners of the Journal, to publish the Work in this journal and any other BMJ products and to exploit all rights, as set out in our licence.

The Submitting Author accepts and understands that any supply made under these terms is made by BMJ to the Submitting Author unless you are acting as an employee on behalf of your employer or a postgraduate student of an affiliated institution which is paying any applicable article publishing charge ("APC") for Open Access articles. Where the Submitting Author wishes to make the Work available on an Open Access basis (and intends to pay the relevant APC), the terms of reuse of such Open Access shall be governed by a Creative Commons licence – details of these licences and which Creative Commons licence will apply to this Work are set out in our licence referred to above.

Other than as permitted in any relevant BMJ Author's Self Archiving Policies, I confirm this Work has not been accepted for publication elsewhere, is not being considered for publication elsewhere and does not duplicate material already published. I confirm all authors consent to publication of this Work and authorise the granting of this licence.

For peer review only

Title Page

Authors: Brett P Dyer, Trishna Rathod-Mistry, Claire Burton, Danielle A van der Windt, Miliça Blagojevic-Bucknall.

Title: Diabetes as a Risk Factor for the Onset of Frozen Shoulder: A Systematic Review and Meta-Analysis.

Affiliation: Primary Care Centre Versus Arthritis, School of Medicine, Keele University, Staffordshire, UK

Corresponding Author: Brett P Dyer. **Email:** b.p.dyer@keele.ac.uk

ORCID ID's: Brett P Dyer: 0000-0001-8039-7631, Trishna Rathod-Mistry: 0000-0002-6369-4746, Claire Burton 0000-0003-4688-3075, Danielle A van der Windt: 0000-0002-7248-6703, Miliça Blagojevic-Bucknall: 0000-0001-7230-7771.

Author contributions: All authors contributed to the conception of the study and systematic review study selection. Data extraction and risk of bias assessment was performed by BPD, MB-B and TR-M. BPD performed the meta-analysis and narrative synthesis of the results and drafted the initial manuscript. All authors contributed to editing and approval of the final manuscript.

Funding: This work was supported by Versus Arthritis grant number 21899. Claire Burton is funded by an NIHR (National Institute for Health Research) Clinical Lectureship. The views expressed are those of the authors and not necessarily those of the National Health Service, the NIHR, or the Department of Health and Social Care.

Ethics approval: No Ethics Committee or Institutional Board approval is required.

Data availability: Data have been included in Table 1 and Appendix Table C.1.

Conflicts of interest statement: The authors have no conflicts of interest.

Word count: 2871 words.

For peer review only

Title

Diabetes as a Risk Factor for the Onset of Frozen Shoulder: A Systematic Review and Meta-Analysis

Abstract

Objective Summarise longitudinal observational studies to determine whether diabetes (types 1 and 2) is a risk factor for the onset of frozen shoulder.

Methods Studies were identified through a systematic literature search of eleven bibliographic databases, reference screening and emailing professional contacts. Longitudinal observational studies that estimated the association between diabetes and developing frozen shoulder were eligible. Risk of bias was judged using the Quality In Prognosis Studies tool. For studies providing sufficient data, random-effects meta-analysis was used to derive summary estimates of the association between diabetes and the onset of frozen shoulder.

Results A meta-analysis of six case-control studies including 5388 people estimated the odds of developing frozen shoulder for people with diabetes to be 3.69 (95% CI: 2.99, 4.56) times the odds for people without diabetes. Two cohort studies were identified, both suggesting diabetes was associated with the onset of frozen shoulder, with hazard ratios of 1.32 (95% CI: 1.22, 1.42) and 1.67 (95% CI: 1.46, 1.91). Risk of bias was judged as high in seven studies and moderate in one study.

Conclusions People with diabetes are more likely to develop frozen shoulder. High-quality studies are needed to confirm the strength and understand reasons for the association.

PROSPERO registration number CRD42019122963.

Keywords: Diabetes, Frozen shoulder, Adhesive capsulitis, Meta-analysis

Strengths and limitations of this study

- This systematic review is the first to summarise the results of longitudinal observational studies estimating the association between diabetes and the onset of frozen shoulder.
- Robust meta-analytic methods were used to synthesise and analyse data.
- Sensitivity to influential estimates and sensitivity to small study bias were assessed.
- Risk of bias was judged as high in seven studies and moderate in one study, limiting the certainty in evidence.
- Only two cohort studies were identified which meant that pooling of association estimates was not suitable.

1 - Introduction

Frozen shoulder, also known as adhesive capsulitis, is a painful and severely debilitating condition. The inflammatory contracture of the glenohumeral joint capsule in frozen shoulder restricts both active and passive range of motion, with loss of external rotation being especially characteristic of this condition [1].

Frozen shoulder generally presents between the ages of 50 and 60 years and rarely presents before 40 years [2]. Women (58%) are more likely to develop frozen shoulder than men (42%) [3]. The contralateral shoulder is also affected in 6% to 17% of patients [4]. Although the exact aetiology remains unclear, several factors have been found to be associated with frozen shoulder, including type 1 and type 2 diabetes and other metabolic factors, trauma, thyroid dysfunction, cardiovascular disease, and other musculoskeletal conditions such as Dupuytren's contracture [5].

For peer review only

The prevalence of frozen shoulder in the general population has been estimated at 2%. However, due to a high rate of misdiagnosis, this figure may be misleading, with the true prevalence of frozen shoulder in the general population more likely to be around 0.75% [1]. A meta-analysis of 13 cross-sectional studies estimated the prevalence of frozen shoulder in populations with diabetes to be 13.4% (95% confidence interval (CI): 10.2%, 17.2%), although there was substantial between-study heterogeneity [6]. Building on the work of (Zreik et al. 2016), this systematic review aims to summarise evidence from longitudinal observational studies to understand the temporal relationship between diabetes and frozen shoulder. It has been hypothesised that diabetes causes frozen shoulder. The evidence of a potential temporal relationship summarised in this systematic review is necessary (although it is not sufficient) to determine whether the association between diabetes and frozen shoulder is causal [7].

2 - Methods

The protocol for this systematic review was registered on PROSPERO (CRD42019122963) and the review was conducted and reported using PRISMA guidelines [8]. A systematic literature search of MEDLINE, EMBASE, AMED, PsycINFO, Web of Science core collection, CINAHL, Epistemonikos, Trip, PEDro, OpenGrey, and The Grey Literature Report was carried out in January 2019 and updated in June 2021. Reference lists of eligible studies were screened. Additionally, a professional contact of one author (DAvdW) was contacted to identify further studies. We retrieved all epidemiological studies containing index terms (e.g. Medical Subject Headings) and free-text words related to diabetes and shoulder pain more generally (not limited to frozen shoulder) to reduce the risk of missing potentially relevant publications. The search strategy for MEDLINE, which was constructed with the support of a health information specialist, can be found in Appendix A.

Reviewer BPD screened all titles and abstracts to check eligibility using the pre-defined inclusion and exclusion criteria, and reviewers MB-B and CB independently checked a 20% random sample. Reviewer BPD checked all full-texts for eligibility using the inclusion and exclusion criteria, and reviewers MB-B, CB, TR-M also independently checked eligibility. Disagreements regarding the inclusion of studies were resolved through discussion with DAvdW.

To be eligible for inclusion, studies were required to have a longitudinal, prospective or retrospective, observational study design. Cohort studies were required to have a study population consisting of people without frozen shoulder at inclusion and must have established whether diabetes was present at baseline (all types of diabetes were considered). Case-control studies were required to have a study population consisting of people with frozen shoulder and a control group without frozen shoulder, with diabetes defined as the exposure of interest. The paper must have presented an odds ratio, risk ratio or hazard ratio, or they must have presented sufficient data to allow the associations to be estimated. There were no restrictions to setting; population based as well as clinical cohorts were eligible. All non-English language papers were assessed by a reviewer with appropriate language skills. Cross-sectional studies and case series were excluded, as were studies where a full text could not be obtained.

Data extraction was completed by reviewer BPD and was independently checked by reviewers MB-B and TR-M. Types of data extracted included details of study design, setting, sample characteristics, exposure/outcome/covariate measurement, inclusion and exclusion criteria, sample size, attrition, covariate conditioning, follow-up time, statistical analysis, association estimates (odds ratio, risk ratio or hazard ratio) or raw data to estimate association sizes if they were not already presented. Risk of bias was independently assessed by pairs of reviewers (BPD, MB-B, TR-M). Risk of bias was judged using the Quality In Prognosis Studies (QUIPS) tool [9]. The QUIPS tool covers six domains: (1) study participation, (2) study attrition, (3) prognostic/risk factor measurement, (4) outcome measurement, (5) study confounding, (6) statistical analysis and reporting. Each of the six domains is scored as being at a low, medium, or high risk of bias [9]. Domain scores were used to guide judgement of the overall risk of bias (scored as low, medium or high) for the study. Overall risk of bias was based on author judgement and the use of a tallied or summated score was avoided. All disagreements regarding data extraction and assessment of risk of bias were resolved by discussion.

For peer review only

Case-control studies and cohort studies were analysed separately. Narrative synthesis was used where less than five studies were present and a random-effects meta-analysis model was used to estimate a summary estimate when five or more studies were present. Where adjusted and crude estimates were both presented, the adjusted estimate was used. Where a zero-cell count was present within the results of a study, a continuity correction of 0.5 was added to all cells for that study. Restricted maximum likelihood estimation [10] was used to estimate the between-study variance, τ^2, and the Hartung-Knapp-Sidik-Jonkman variance correction method [11] was used in the estimation of the pooled effect confidence interval. Heterogeneity was assessed using Cochran's Q statistic, complemented by the I^2 index [12]. Prediction intervals were not estimated since they are inaccurate when there is little heterogeneity ($I^2 < 0.3$), or an imbalance in study sizes exists, both of which were found in the meta-analysis in this review (see Section 3) [13]. Evidence of small-study bias was assessed with a funnel plot of log odds ratios against their standard errors [14]. A test for funnel plot asymmetry was not used since the meta-analysis included less than ten studies [15]. The influence of each study on the overall pooled estimate was assessed by repeating the meta-analysis, each time leaving out a single study [16]. Statistical analysis was carried out using Stata version 16.1 [17].

2.1 - Patient and Public Involvement

No patient involved.

3 - Results

The searches identified 1784 unique citations, 12 of which were selected for full-text screening, and eight studies consisting of a total of 346,278 people fulfilled the inclusion criteria (Figure 1). Table 1 summarises information on risk of bias, study design, setting, participants, sample size, and methods used for diagnosing diabetes and frozen shoulder. Of the eight studies that met the criteria for inclusion, six had case-control designs and two had cohort designs. Three studies (including the two cohort studies) collected information from electronic health records, four studies were hospital-based, and one study was based in a physical therapy clinic. Among the case-control studies, the percentage of female cases ranged from 52% to 75% and the mean age for cases ranged from 52.8 years to 57.2 years.

Table 1. Characteristics of Studies on diabetes as a risk factor for the onset of frozen shoulder

Source	Risk of Bias (QUIPS, overall assess-ment)	Design and Setting	% Female	Mean Age (years)	Sample Size	Method to diagnose diabetes and frozen shoulder	Variables conditioned on
**Case-control studies**							
K. L. Boyle-Walker, et al., 1997 [18]	High	Sex-Matched Case-Control at Physical Therapy Clinic in the USA	Case Group: 75%, Control Group: 68%	Not reported	Cases: 32, Controls: 31	Diabetes: Self-reported Questionnaire Frozen shoulder: Clinically diagnosed	Sex-matched
W. Li, et al., 2014 [19]	High	Hospital based case-control matched on time of hospitalisation in China	Case Group: 63%, Control Group: 55%	Cases: 57.2, Controls: 45.9	Cases: 182, Controls: 196	Diabetes: Face-to-face interview Frozen shoulder: Clinically diagnosed	Matched on time of hospitalisation, adjusted for history of minor shoulder trauma
S-Y. Lee, et al., 2012 [20]	High	Hospital based age- and	Case Group:	Cases: 52.8,	Cases: 40, Controls:	Diabetes: Unclear Frozen	Age- and sex-matched

		sex-matched case-control in South Korea	55%, Control Group: not reported	Controls: not reported	40	shoulder: Clinically diagnosed	
C. Milgrom, et al., 2008 [21]	High	Hospital based age-matched case-control in Israel	Case Group: 60%, Control Group: 65%	Cases: 54.9, Controls: 55.4	Cases: 126, Controls: 98	Diabetes: If patient was receiving drug treatment for Diabetes or whose serum glucose was higher than 200 mg/dl Frozen shoulder: Clinically diagnosed	Age-matched
K. Wang, et al., 2013 [22]	High	Hospital based age- and sex-matched case-control in Australia	Case Group: 64%, Control Group: 58%	Cases: 56, Controls: 55.3	Cases: 87, Controls: 176	Diabetes: Self-reported Frozen shoulder: Clinically diagnosed	Age- and sex-matched
K. Kingston, et al., 2018 [23]	High	Sex-matched case-control using electronic health records in the USA	Case Group: 58%, Control Group: 58%	Cases: 56.4, Controls: Not Reported	Cases: 2190, Controls: 2190	Diabetes: ICD-9 Code Frozen shoulder: ICD-9 Code	Sex-matched
**Cohort studies**							
Y-P. Huang, et al., 2013 [24]	High	Age- and sex-matched cohort with 3-year follow-up using electronic health records in Taiwan	Exposed Group: 47%, Non-Exposed Group: 47%	Exposed Group: 55.7, Non-Exposed Group: 55.5	Exposed Group: 78827, Non-Exposed Group: 236481	Diabetes: ICD-9 Code Frozen shoulder: ICD-9 Code	Age- and sex-matched. Multivariable analysis adjusted for age, sex, dyslipidaemia
S-F. Lo, et al., 2013 [25]	Moderate	Cohort with 8-year follow-up using electronic health records in Taiwan	Exposed Group: 52%, Non-Exposed Group: 51%	Not reported	Exposed Group: 5109, Non-Exposed Group: 20473	Diabetes: ICD-9 Code Frozen shoulder: ICD-9 Code	Multivariable analysis adjusted for age, income, stroke, hypertension, hyperlipidaemia, obesity, chronic obstructive pulmonary disease

Presence of diabetes was identified using ICD-9 codes (codes to classify diseases, symptoms, clinical findings and causes of disease and injury) from electronic health records in three studies, self-reported in three studies, identified with a glucose test or if the patient was receiving drug treatment for diabetes in one study, and was unclear in one study. Frozen shoulder was identified using ICD-9 codes in three studies and was

For peer review only

diagnosed clinically in five studies. Reporting of the types of diabetes was poor, with only one study doing so. (Lo et al. 2013) stated that 296 (5.8%) of the 5109 people with diabetes in their study had type 1 diabetes. Two studies were conducted in Taiwan, two in the USA and the remaining four were conducted in China, South Korea, Israel and Australia.

Overall QUIPS risk of bias scores for each study can be found in Table 1 and full QUIPS assessments can be found in Appendix Table B1. Overall, there was a 75% agreement between reviewers across the individual bias domains, and reviewers agreed on 4 of the 8 overall risk of bias scores. One of the cohort studies [25] was scored as being at a moderate risk of bias for their overall study rating and the other seven studies were rated as being at a high risk of bias overall. A bar graph of the scores for individual risk of bias domains can be found in Appendix Figure B1. Risk of bias was generally high across most domains, but especially so for the risk of unaccounted confounding, which was scored as being at a high risk of bias in all eight studies. Five of the case-control studies [18,20-23] only accounted for age, gender or a combination of the two. One study [19] matched on the time of hospitalisation and adjusted for history of minor shoulder trauma. One cohort study [24] adjusted for age, sex and dyslipidaemia; the other cohort study [25] adjusted for age, income, stroke, hypertension, hyperlipidaemia, obesity and chronic obstructive pulmonary disease.

Six case-control studies including a total of 5388 people were pooled in a random-effects meta-analysis, with a pooled odds ratio of 3.69 (95% CI: 2.99, 4.56) (Figure 2). The raw data extracted from each study that was used to calculate odds ratios can be found in Appendix Table C1. The estimated between-study variance was small (τ^2<0.01, 95% CI: <0.01, 0.23) and little heterogeneity was detected (Q=2.07, df=5, p=0.84; I^2 <0.01% (95% CI: <0.1%, 67.6%)), but the estimate for I^2 is imprecise as indicated by the wide 95% confidence interval. The influence analysis showed that excluding the largest study (K. Kingston et al. 2018), which contained 4380 of the 5388 participants, greatly reduces the precision of the pooled estimate but did not substantially affect the value of the pooled estimate (Figure 3). Further, excluding any other single study did not substantially affect the value of the pooled estimate (Figure 3). The two studies with the smallest standard errors for their effect estimates had the largest odds ratio's, making the funnel plot appear unsymmetrical. However, due to the small number of studies contributing to the funnel plot, the asymmetrical appearance could be due to chance (Figure 4).

The two cohort studies that were identified used Cox proportional-hazards models and obtained results suggesting that people with diabetes were more at risk of developing frozen shoulder. One cohort study [24] using electronic health records from Taiwan, with a 3-year follow-up and consisting of 315,308 people reported an age-, sex- and dyslipidaemia-adjusted hazard ratio of 1.32 (95% CI: 1.22, 1.42). Another cohort study [25], with an 8-year follow-up, consisting of 25,582 people, also using electronic health records from Taiwan, estimated an age-, income-, stroke-, hypertension-, hyperlipidaemia-, obesity- and chronic obstructive pulmonary disease- adjusted hazard ratio of 1.67 (95% CI: 1.46, 1.91).

4 - Discussion

This systematic review consists of eight studies each demonstrating evidence to suggest that diabetes is associated with the onset of frozen shoulder. Our meta-analysis of six case-control studies yielded a pooled odds ratio of 3.69 (95% CI: 2.99, 4.56), and the value of the pooled estimate was robust to the omission of any individual study. The odds ratio estimates of all but one study (Kingston et al. 2018) were imprecise with large confidence intervals; this meant that the confidence intervals overlapped well, resulting in a small I^2 value. It is also important to note that Cochran's Q statistic should be interpreted with caution since the number of studies included in the analysis was small [26].

The funnel plot appeared to show a slight asymmetry. Given that a small number of studies were available, it is difficult to assess accurately whether any small-study bias was present or if the appearance was due to chance. However, since our influence analysis has shown that the inclusion/exclusion of any individual study had very little impact on the pooled effect estimate, any potential small-study bias would be unlikely to substantially affect the results.

For peer review only

Two cohort studies were identified, both of which corroborate the evidence from the six case-control studies reported above, that people with diabetes are more likely to develop frozen shoulder than those without diabetes. Of the two cohort studies, one was deemed to be at a high risk of bias and the other at a moderate risk of bias. The hazard ratios in the two studies did differ, which could have partly been due to the differences in the covariates that were adjusted for and/or the differences in the duration of follow-up. Both studies were rated as being at a high of bias for the outcome-measurement domain as the length of follow-up (3 years [24] and 8 years [25]) was deemed too short to establish whether a patient would develop frozen shoulder in the future. Previous studies have suggested that the duration of diabetes may be associated with the risk of developing frozen shoulder [27,28], with one of the cohort studies in this review also stating that their study *"suggests that the development of [frozen shoulder] is associated with the duration of diabetes"* [24]. Therefore, future studies should ensure that the follow-up period is long enough to observe participants from diabetes diagnosis through to the ages for which frozen shoulder is common. A cross-sectional study of 1,373 patients presenting with frozen shoulder estimated that the mean age of onset for frozen shoulder was 55.4 years with a standard deviation of 9.9 years [3].

Another important limitation of the studies included in this review was the overall poor adjustment for confounding variables. All eight studies were rated as being at a high risk of unaccounted confounding. In each study, confounders were either ignored [18,20-24] or inappropriate statistical methods, such as univariable prefiltering and stepwise selection, were used [19,24,25]. These methods are especially poorly suited for aetiologic models [29]. Thus, these studies may have missed potentially important confounders [19,24,25] or erroneously adjusted for mediators, such as stroke [25].

Previously, a meta-analysis of cross-sectional studies established that frozen shoulder was more prevalent in people with diabetes than among people without diabetes. This systematic review provides evidence of a temporal relationship between diabetes and frozen shoulder. Understanding the temporal relationship is key to explaining why diabetes and frozen shoulder are associated, however further high-quality research with appropriate methods and study design is required to confirm the strength of the association and establish whether diabetes is indeed a cause of frozen shoulder.

Whilst sound and reliable epidemiological evidence of a causal relationship between diabetes and frozen shoulder is currently unavailable, elsewhere in the literature, researchers have hypothesised about potential pathological mechanisms through which diabetes may lead to frozen shoulder. Current evidence, based on histological studies, suggests that a pathophysiological process consisting of chronic inflammation and capsular fibrosis leads to the contracture in frozen shoulder [30,31]. It has been hypothesised that the accumulation of advanced glycation end products (AGE's), which lead to the cross-linking of collagen [32,33], may explain the fibrosis in the capsule of frozen shoulder patients [34]. Glycation is a process by which simple sugars bond to proteins, which is enhanced by persistent hyperglycaemia. Thus, the role of glycation and AGE's in the fibrosis of the shoulder capsule could potentially be a reason why diabetes is associated with the onset of frozen shoulder. Another potential reason why diabetes may be associated with frozen shoulder is that hyperglycaemia may induce proinflammatory cytokines [35] which have been found to be elevated in the capsule and synovium of frozen shoulder patients [36].

The association between glycaemic control and the risk of developing frozen shoulder should also be a focus for future research. One study found evidence to suggest that poor long-term glycaemic control in people with diabetes is associated with an increased incidence of frozen shoulder [37], whilst another study found no association between HbA1c level in people with diabetes and the prevalence of frozen shoulder [38]. Further research is required to investigate whether glycaemic control is associated with the onset of frozen shoulder.

High quality epidemiological research is required to better understand the association between diabetes and frozen shoulder. Further research should clearly and transparently report the methods through which adjustment sets are selected whilst using a model-building strategy that is appropriate for the research question of interest. Additionally, there is a lack of prospective studies investigating the association between diabetes and the onset of frozen shoulder; we identified only two prospective studies in this review, both of

For peer review only

which were from Taiwan. Future studies with prospective designs will help to gauge whether the findings of these two cohort studies are reproducible, and whether the results are consistent across different populations.

5 - Conclusion

This systematic review provides evidence that people with diabetes are more at risk of developing frozen shoulder than those without diabetes. However, high-quality cohort studies with sufficiently long follow-up and appropriate adjustment for confounders are required to better understand the association of diabetes with the onset of frozen shoulder. Given the evidence in this review, clinicians should consider checking whether patients with diabetes are experiencing musculoskeletal pain at their routine follow-up appointments.

6 - Acknowledgements

This study was supported by the Versus Arthritis PhD scholarship scheme [grant number 21899]. The author would also like to thank the information specialists of evidence synthesis team at the Primary Care Centre Versus Arthritis for their advice regarding the search strategy, and Elaine Willmore for taking time to check the results of our searches for this review. Further thanks are given to Dr Linda Chesterton, who was involved in the initial conceptualisation of this study. Claire Burton is funded by an NIHR (National Institute for Health Research) Clinical Lectureship. The views expressed are those of the authors and not necessarily those of the National Health Service, the NIHR, or the Department of Health and Social Care.

For peer review only

Abbreviations

QUIPS - Quality In Prognosis Studies

CI – Confidence interval

Q – Cochran's Q statistic

df – Degrees of freedom

Ethics Approval

No Ethics Committee or Institutional Board approval is required.

Author contributions

All authors contributed to the conception of the study and systematic review study selection. Data extraction and risk of bias assessment was performed by BPD, MB-B and TR-M. BPD performed the meta-analysis and narrative synthesis of the results and drafted the initial manuscript. All authors contributed to editing and approval of the final manuscript.

Funding

This work was supported by Versus Arthritis grant number 21899. Claire Burton is funded by an NIHR (National Institute for Health Research) Clinical Lectureship. The views expressed are those of the authors and not necessarily those of the National Health Service, the NIHR, or the Department of Health and Social Care.

Data availability

Data have been included in Table 1 and Appendix Table C.1.

Conflicts of interest statement

The authors have no conflicts of interest.

Supplementary Material

Appendix A contains the search strategy for MEDLINE.

Appendix B contains full QUIPS risk of bias assessments for each primary study and a bar graph of QUIPS scores for each of the six bias domains.

Appendix C contains the raw data extracted from each primary study that was used to calculate the odds ratios included in the meta-analysis.

References

[1] Bunker T. Frozen Shoulder. *Orthopaedics and Trauma*. 2011; 25(1):11-8. https://doi.org/10.1016/j.mporth.2011.01.007

[2] Dias R, Cutts S, Massoud S. Frozen shoulder. *BMJ*. 2005; 331:1453-6. https://doi.org/10.1136/bmj.331.7530.1453

[3] Cho C-H, Koo TW, Cho N-S, Park K-J, Lee BG, Shin D, Choi S, Cho S-H, Kim M-S, Ko S-H, Kim C-H, Park J-Y, Yoo Y-S. Demographic and Clinical Characteristics of Primary Frozen Shoulder in a Korean Population. *Clin Shoulder Elbow*. 2015; 18(3):133-7. doi: 10.5397/cise.2015.18.3.133

[4] Rizk TE, Pinals RS. Frozen shoulder. *Seminars in Arthritis and Rheumatism.* 1982; 11(4):440-52. doi: 10.1016/0049-0172(82)90030-0

[5] Cucchi D, Marmotti A, De Giorgi S, Costa A, D'Apolito R, Conca M, Russo A, Saccomanno MF, de Girolamo L. Risk Factors for Shoulder Stiffness: Current Concept. *Joints*. 2017; 5(4):217-23. doi: 10.1055/s-0037-1608951

[6] Zreik NH, Malik RA, Charalambos CP. Adhesive capsulitis of the shoulder and diabetes: a meta-analysis of prevalence. *MLTJ.* 2016; 6(1):26-34. doi: 10.11138/mltj/2016.6.1.026

[7] Rothman KJ, Greenland S, Lash TL. *Modern Epidemiology*: 3rd ed. Lippincott Williams & Wilkins. 2013.

For peer review only

[8] Moher D, Liberati A, Tetzlaff J, Altman DG. Preferred reporting items for systematic reviews and meta-analyses: the PRISMA statement. *BMJ.* 2009; 339:b2535. doi: 10.1136/bmj.b2535

[9] Hayden JA, van der Windt DA, Cartwright JL, Côté P, Bombardier C. Assessing bias in studies of prognostic factors. *Ann Intern Med*. 2019; 158(4):280–6. doi: 10.7326/0003-4819-158-4-201302190-00009

[10] Veroniki AA, Jackson D, Viechtbauer W, Bender R, Bowden J, Knapp G, Kuss O, Higgins JPT, Langan D, Salanti G. Methods to estimate the between-study variance and its uncertainty in meta-analysis. *Res Synth Meth. 2016;* 7(1):55-79. doi: 10.1002/jrsm.1164

[11] IntHout J, Ioannidis JPA, Borm GF. The Hartung-Knapp-Sidik-Jonkman method for random-effects meta-analysis is straightforward and considerably outperforms the standard Der-Simonian-Laird method. *BMC Med Res Methodol*. 2014; 14:25. https://doi.org/10.1186/1471-2288-14-25

[12] Higgins JPT, Thompson SG, Deeks JJ, Altman DG. Measuring inconsistency in meta-analyses. *BMJ.* 2003; 327:557-60. https://doi.org/10.1136/bmj.327.7414.557

[13] Partlett C, Riley RD. Random effects meta-analysis: Coverage performance of 95% confidence and prediction intervals following REML estimation. *Statist. Med*. 2016; 36(2):301-17. https://doi.org/10.1002/sim.7140

[14] Sterne JAC, Harbord RM. Funnel Plots in Meta-analysis. *Stata Journal*. 2004; 4(2):127-41. https://doi.org/10.1177/1536867X0400400204

[15] Higgins JPT, Green S. Cochrane Handbook for Systematic Reviews of Interventions Version 5.1.0 [updated March 2011]. *The Cochrane Collaboration*. 2011.

[16] Viechtbauer W, Cheung MWL. Outlier and influence diagnostics for meta-analysis. *Res Syn Meth. 2010;* 1(2):112-25. doi: 10.1002/jrsm.11

[17] StataCorp. 2019. *Stata Statistical Software: Release 16.* College Station, TX: StataCorp LLC.

[18] Boyle-Walker KL, Gabard DL, Bietsch E, Masek-VanArsdale DM, Robinson BL. A profile of patients with adhesive capsulitis. *J Hand Ther*. 1997; 10(3):222-8. https://doi.org/10.1016/S0894-1130(97)80025-7

[19] Li W, Lu N, Xu H, Wang H, Huang J. Case control study of risk factors for frozen shoulder in China. *Int J Rheum Dis*. 2014; 18(5):508-13. https://doi.org/10.1111/1756-185X.12246

[20] Lee S-Y, Park J, Song S-W. Correlation of MR Arthrographic Findings and Range of Shoulder Motions in Patients With Frozen Shoulder. *American Journal of Roentgenology*. 2012; 198(1):173-9. doi: 10.2214/AJR.10.6173

[21] Milgrom C, Novack V, Weil Y, Jaber S, Radeva-Petrova DR, Finestone A. Risk factors for idiopathic frozen shoulder. *Isr Med Assoc J*. 2008; 10(5):361-4. PMID: 18605360

[22] Wang K, Ho V, Hunter-Smith DJ, Beh PS, Smith KM, Weber AB. Risk factors in idiopathic adhesive capsulitis: a case control study. *J Shoulder Elbow Surg*. 2013; 22(7):24-9. doi: 10.11138/mltj/2016.6.1.026

[23] Kingston K, Curry EJ, Galvin JW, Li X. Shoulder adhesive capsulitis: epidemiology and predictors of surgery. *J Shoulder Elbow Surg*. 2018; 27(8):1437-43. doi: 10.1016/j.jse.2018.04.004

[24] Huang Y-P, Fann C-Y, Chiu Y-H, Yen M-F, Chen L-S, Chen H-H, Pan S-L. Association of Diabetes Mellitus With the Risk of Developing Adhesive Capsulitis of the Shoulder: A Longitudinal Population-Based Follow up Study. *Arthritis Care & Research*. 2013; 65(7):1197-202. https://doi.org/10.1002/acr.21938

[25] Lo S-F, Chu S-W, Muo C-H, Meng N-H, Chou L-W, Huang W-C, Huang C-M, Sung F-C. Diabetes mellitus and accompanying hyperlipidemia are independent risk factors for adhesive capsulitis: a nationwide population-based cohort study. *Rheumatology International*. 2014; 34:67-74. doi: 10.1007/s00296-013-2847-4

For peer review only

[26] Hardy RJ, Thompson SG. Detecting and describing heterogeneity in meta-analysis. *Stat Med*. 1998; 17(8):841-56. doi: 10.1002/(sici)1097-0258(19980430)17:8<841::aid-sim781>3.0.co;2-d

[27] Thomas SJ, McDougall C, Brown IDM, Jaberoo M-C, Stearns A, Ashraf R, Fisher M, Kelly IG. Prevalence of symptoms and signs of shoulder problems in people with diabetes mellitus. *J Shoulder and Elbow Surg.* 16(6); 748-51. doi: 10.1016/j.jse.2007.02.133

[28] Bridgeman JF, Periarthritis of the shoulder and diabetes mellitus. *Ann Rheum Dis.* 31: 69-71; 1972. doi: 10.1136/ard.31.1.69

[29] Heinze G, Dunkler D. Five myths about variable selection. *Transplant International*. 2017; 30(1):6-10. https://doi.org/10.1111/tri.12895

[30] Wong PL, Tan HC. A Review on Frozen Shoulder. *Singapore Med J*. 2010; 51(9):694-7. PMID: 20938608

[31] Cho C-H, Song K-S, Kim B-S, Kim D.H, Lho Y-M. Biological Aspect of Pathophysiology for Frozen Shoulder. *BioMed Research International*. 2018; Article ID 7274517. https://doi.org/10.1155/2018/7274517

[32] Hwang KR, Murrell GAC, Millar NL, Bonar F, Lam R, Walton JR. Advanced glycation end products in idiopathic frozen shoulders. *J Shoulder and Elbow Surg.* 2016; 25(6): 981-8. doi: 10.1016/j.jse.2015.10.015

[33] Goldin A, Beckman JA, Schmidt AM, Creager MA. Advanced Glycation End Products. *Circulation*. 2006; 114(6): 597-605. doi: 10.1161/CIRCULATIONAHA.106.621854.

[34] Hsu C-L, Sheu WH-H. Diabetes and shoulder disorders. *J Diabetes Investig*. 2016; 7(5):649-51. https://doi.org/10.1111/jdi.12491

[35] Shanmugam N, Reddy MA, Guha M, Natarajan R. High glucose-induced expression of proinflammatory cytokine and chemokine genes in monocytic cells. *Diabetes*. 2003; 52(5):1256-64. doi: 10.2337/diabetes.52.5.1256

[36] Lho Y-M, Ha E, Cho C-H, Song K-S, Min B-W, Bae K-C, Lee K-J, Hwang I, Park H-B. Inflammatory cytokines are overexpressed in the subacromial bursa of frozen shoulder. *J Shoulder and Elbow Surg.* 2013; 22(5): 666-72. https://doi.org/10.1016/j.jse.2012.06.014

[37] Chan JH, Ho BS, Alvi HM, Saltzman MD, Marra G. The relationship between the incidence of adhesive capsulitis and hemoglobin A1c. *J Shoulder and Elbow Surg.* 2017; 26(10):1834-7. https://doi.org/10.1016/j.jse.2017.03.015

[38] Yian EH, Contreras R, Sodl JF. Effects of Glycemic Control on Prevalence of Diabetic Frozen Shoulder. *J Bone Joint Surg.* 2012; 94(10):919-23. doi: 10.2106/JBJS.J.01930

For peer review only

Figure Captions

Fig. 1 PRISMA flow diagram summarising record identification and study selection.

Fig. 2 Random effects meta-analysis of the association between diabetes and the odds of developing frozen shoulder.

Fig. 3 Influence plot showing the result of repeating the original meta-analysis (Figure 2), each time with a different primary study removed.

Fig. 4 Funnel plot of log odds ratios for developing frozen shoulder in people with diabetes vs. those without diabetes.

For peer review only

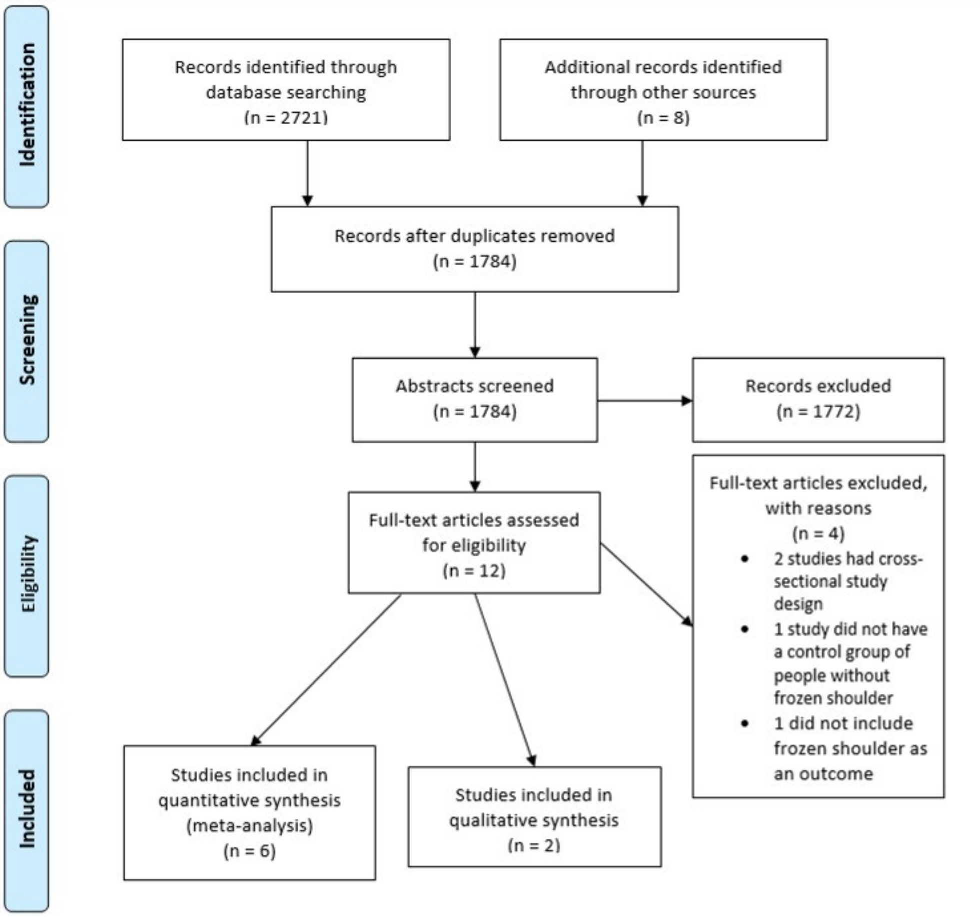

PRISMA flow diagram summarising record identification and study selection.

132x124mm (144 x 144 DPI)

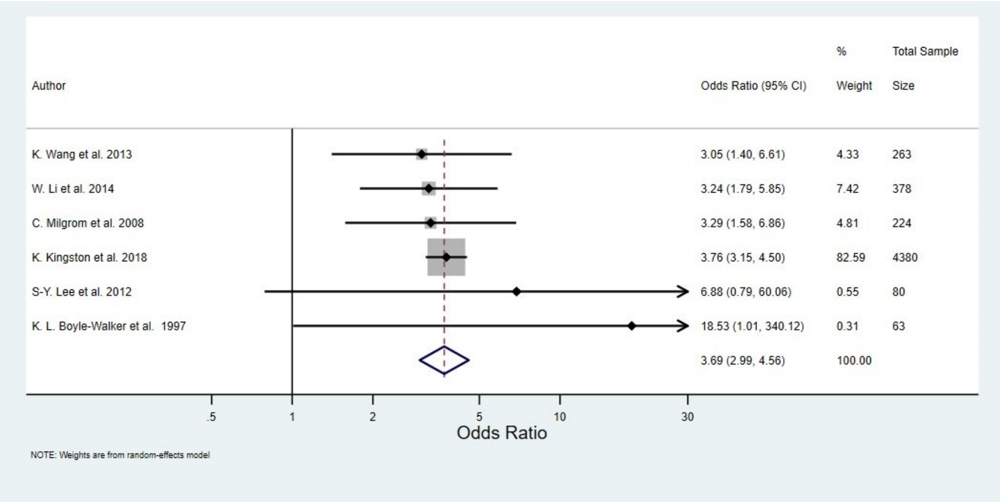

Random effects meta-analysis of the association between diabetes and the odds of developing frozen shoulder.

277x138mm (96 x 96 DPI)

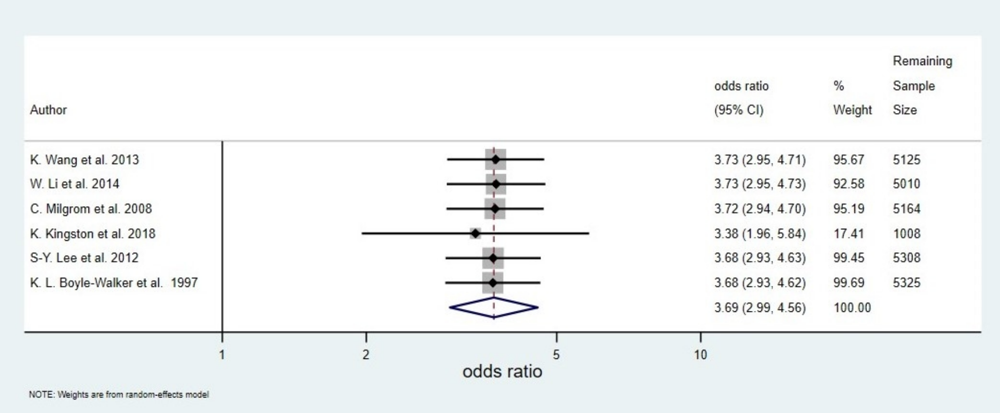

Influence plot showing the result of repeating the original meta-analysis (Figure 2), each time with a different primary study removed.

275x113mm (96 x 96 DPI)

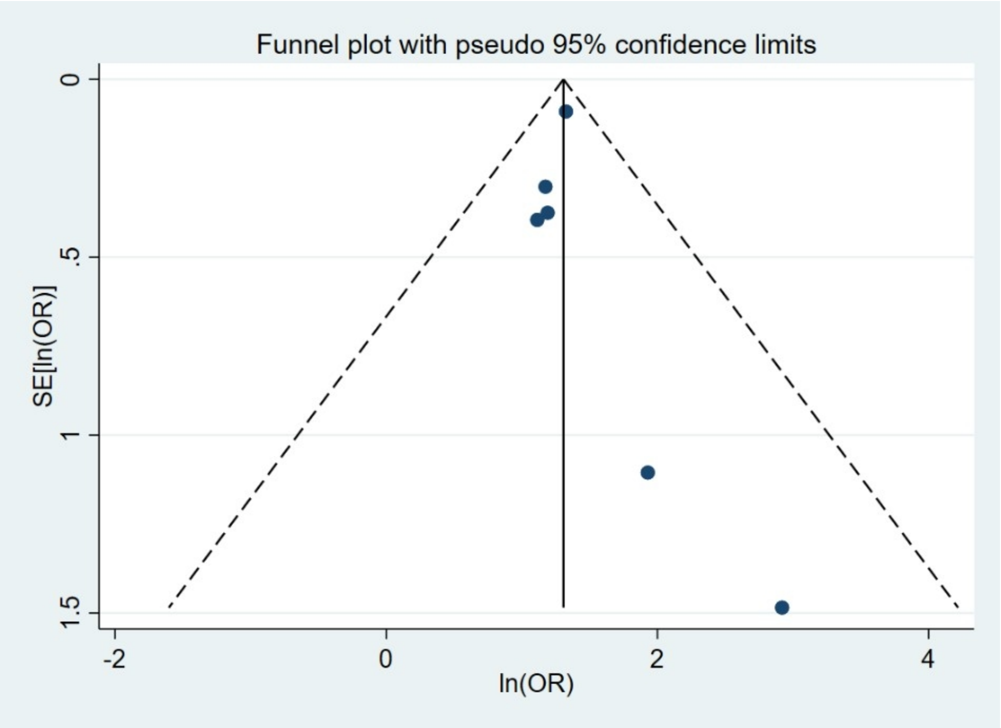

Funnel plot of log odds ratios for developing frozen shoulder in people with diabetes vs. those without diabetes.

109x79mm (220 x 220 DPI)

Appendix A

Interface: OVID. Updated systematic review search conducted on June 2021.

1. ((shoulder* or glenohumer* or subacromi* or acromi* or rotator cuff) adj3 (instability or bursitis or frozen or impinge* or tendonitis or tendinitis or pain* or osteoarthr* or periarthriti* or peri arthriti* or arthralgia)).ti,ab,kw.

2. Shoulder Impingement Syndrome/

3. exp Bursitis/

4. Rotator Cuff/

5. adhesive capsuliti*.ti,ab,kw.

6. Shoulder Pain/

7. or/1-6

8. exp Pain/

9. pain*.ti,ab,kw.

10. Arthralgia/

11. arthralgia.ti,ab,kw.

12. or/8-11

13. Shoulder/

14. Shoulder joint/

15. Acromioclavicular Joint/

16. (shoulder* or glenohumer* or subacromi* or acromi* or rotator cuff).ti,ab,kw.

17. or/13-16

18. 12 and 17

19. 7 or 18

20. exp Diabetes Mellitus/

21. diabet*.ti,ab,kw.

22. (DMi or DM i).ti,ab,kw.

23. (DM1 or DM 1).ti,ab,kw.

24. (DM2 or DM 2).ti,ab,kw.

25. (DMii or DM ii).ti,ab,kw.

26. (DM adj2 type).ti,ab,kw.

27. or/20-26

28. 19 and 27

29. exp animals/ not humans/

30. 28 not 29

For peer review only

Appendix B

Table B.1 QUIPS domain scores for each primary study

Source	Participation	Study Attrition	Risk Factor Measure-ment	Outcome Measure-ment	Confounding	Statistical Analysis and Presentation	Overall Risk of Bias
**Case-Control Studies**							
K. L. Boyle-Walker, et al., 1997 [18]	High	Moderate	High	Moderate	High	Moderate	High
W. Li, et al., 2014 [19]	Moderate	Low	Moderate	High	High	High	High
S-Y. Lee, et al., 2012 [20]	Moderate	Low	Moderate	Moderate	High	Moderate	High
C. Milgrom, et al., 2008 [21]	Moderate	Low	Low	Low	High	Low	High
K. Wang, et al., 2013 [22]	Low	Low	Low	Low	High	Low	High
K. Kingston, et al., 2018 [23]	Low	Moderate	Moderate	Low	High	Moderate	High
**Cohort studies**							
Y-P. Huang, et al., 2013 [24]	Low	Moderate	Low	High	High	High	High
S-F. Lo, et al., 2013 [25]	Low	Low	Low	Moderate	High	Low	Moderate

Fig. B.1 Bar graph of QUIPS scores for each of the six bias domains: study participation, study attrition, diabetes/risk factor (RF) measurement, frozen shoulder/outcome measurement, study confounding, statistical analysis and reporting.

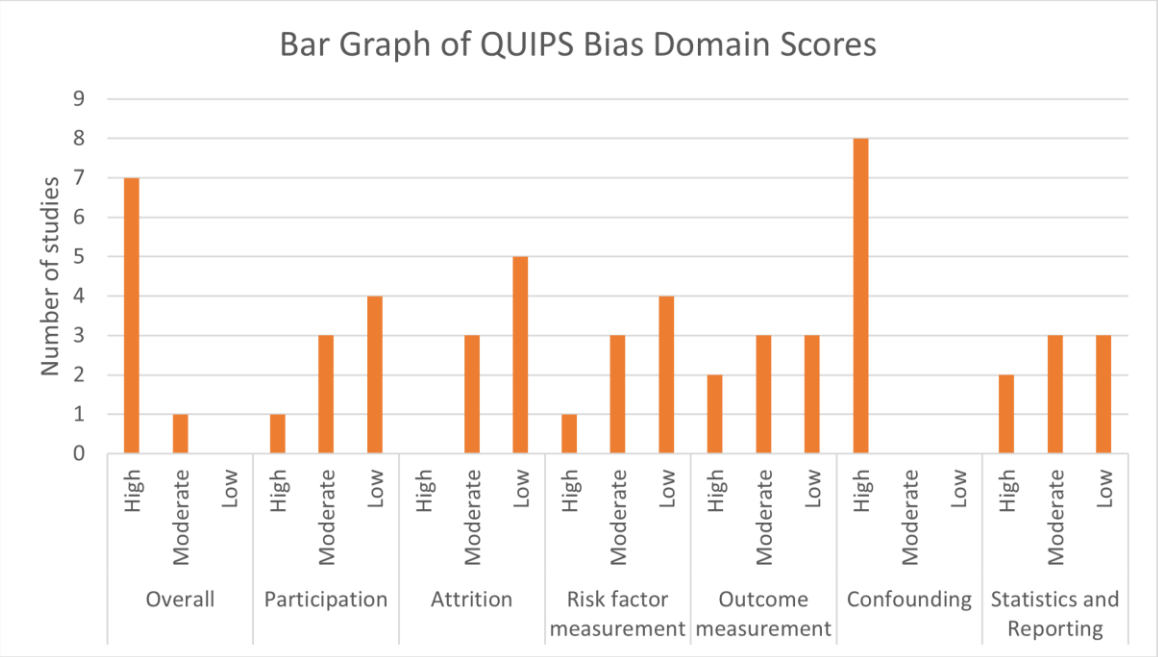

For peer review only

Appendix C

**Table C.1** Raw data from each study.				
**Case-Control Studies**				
Source	Number of cases	Number of controls	Number of cases with diabetes	Number of controls with diabetes
K. L. Boyle-Walker, et al., 1997 [18]	32	31	7	0
W. Li, et al., 2014 [19]	182	196	44	18
S-Y. Lee, et al., 2012 [20]	40	40	6	1
C. Milgrom, et al., 2008 [21]	126	98	37	11
K. Wang, et al., 2013 [22]	87	176	17	13
K. Kingston, et al., 2018 [23]	2190	2190	572	188
**Cohort studies**				
Source	Number of people with diabetes	Number of controls	Number of people with diabetes that developed frozen shoulder	Number of people without diabetes that developed frozen shoulder
Y-P. Huang, et al., 2013 [24]	78827	236481	946	2254
S-F. Lo, et al., 2013 [25]	5109	20473	553	768

For peer review only

PRISMA 2009 Checklist

Section/topic	#	Checklist item	Reported on page #
**TITLE**			
Title	1	Identify the report as a systematic review, meta-analysis, or both.	1
**ABSTRACT**			
Structured summary	2	Provide a structured summary including, as applicable: background; objectives; data sources; study eligibility criteria, participants, and interventions; study appraisal and synthesis methods; results; limitations; conclusions and implications of key findings; systematic review registration number.	1
**INTRODUCTION**			
Rationale	3	Describe the rationale for the review in the context of what is already known.	1
Objectives	4	Provide an explicit statement of questions being addressed with reference to participants, interventions, comparisons, outcomes, and study design (PICOS).	2
**METHODS**			
Protocol and registration	5	Indicate if a review protocol exists, if and where it can be accessed (e.g., Web address), and, if available, provide registration information including registration number.	2
Eligibility criteria	6	Specify study characteristics (e.g., PICOS, length of follow-up) and report characteristics (e.g., years considered, language, publication status) used as criteria for eligibility, giving rationale.	2
Information sources	7	Describe all information sources (e.g., databases with dates of coverage, contact with study authors to identify additional studies) in the search and date last searched.	2
Search	8	Present full electronic search strategy for at least one database, including any limits used, such that it could be repeated.	Appendix A
Study selection	9	State the process for selecting studies (i.e., screening, eligibility, included in systematic review, and, if applicable, included in the meta-analysis).	2
Data collection process	10	Describe method of data extraction from reports (e.g., piloted forms, independently, in duplicate) and any processes for obtaining and confirming data from investigators.	2
Data items	11	List and define all variables for which data were sought (e.g., PICOS, funding sources) and any assumptions and simplifications made.	2
Risk of bias in individual studies	12	Describe methods used for assessing risk of bias of individual studies (including specification of whether this was done at the study or outcome level), and how this information is to be used in any data synthesis.	2
Summary measures	13	State the principal summary measures (e.g., risk ratio, difference in means).	2
Synthesis of results	14	Describe the methods of handling data and combining results of studies, if done, including measures of consistency (e.g., I^2) for each meta-analysis.	3

For peer review only - http://bmjopen.bmj.com/site/about/guidelines.xhtml

PRISMA 2009 Checklist

Section/topic	#	Checklist item	Reported on page #
Risk of bias across studies	15	Specify any assessment of risk of bias that may affect the cumulative evidence (e.g., publication bias, selective reporting within studies).	2
Additional analyses	16	Describe methods of additional analyses (e.g., sensitivity or subgroup analyses, meta-regression), if done, indicating which were pre-specified.	3
**RESULTS**			
Study selection	17	Give numbers of studies screened, assessed for eligibility, and included in the review, with reasons for exclusions at each stage, ideally with a flow diagram.	3, Fig 1
Study characteristics	18	For each study, present characteristics for which data were extracted (e.g., study size, PICOS, follow-up period) and provide the citations.	Table 1
Risk of bias within studies	19	Present data on risk of bias of each study and, if available, any outcome level assessment (see item 12).	6, Appendix C
Results of individual studies	20	For all outcomes considered (benefits or harms), present, for each study: (a) simple summary data for each intervention group (b) effect estimates and confidence intervals, ideally with a forest plot.	6, Fig 2
Synthesis of results	21	Present results of each meta-analysis done, including confidence intervals and measures of consistency.	6, Fig 2
Risk of bias across studies	22	Present results of any assessment of risk of bias across studies (see Item 15).	Appendix C
Additional analysis	23	Give results of additional analyses, if done (e.g., sensitivity or subgroup analyses, meta-regression [see Item 16]).	6, Fig 3
**DISCUSSION**			
Summary of evidence	24	Summarize the main findings including the strength of evidence for each main outcome; consider their relevance to key groups (e.g., healthcare providers, users, and policy makers).	7
Limitations	25	Discuss limitations at study and outcome level (e.g., risk of bias), and at review-level (e.g., incomplete retrieval of identified research, reporting bias).	7
Conclusions	26	Provide a general interpretation of the results in the context of other evidence, and implications for future research.	8
**FUNDING**			
Funding	27	Describe sources of funding for the systematic review and other support (e.g., supply of data); role of funders for the systematic review.	8

From: Moher D, Liberati A, Tetzlaff J, Altman DG, The PRISMA Group (2009). Preferred Reporting Items for Systematic Reviews and Meta-Analyses: The PRISMA Statement. PLoS Med 6(7): e1000097. doi:10.1371/journal.pmed1000097

For peer review only - http://bmjopen.bmj.com/site/about/guidelines.xhtml

PRISMA 2009 Checklist

For more information, visit: www.prisma-statement.org.

Page 2 of 2

For peer review only

BMJ Open

Diabetes as a Risk Factor for the Onset of Frozen Shoulder: A Systematic Review and Meta-Analysis

Journal:	*BMJ Open*
Manuscript ID	bmjopen-2022-062377.R1
Article Type:	Original research
Date Submitted by the Author:	26-Sep-2022

Complete List of Authors:	Dyer, Brett; Keele University, Primary Care Centre Versus Arthritis, School of Medicine<br>Rathod, Trishna; Keele University, Primary Care Centre Versus Arthritis, School of Medicine<br>Burton, Claire; Keele University, Primary Care Centre Versus Arthritis, School of Medicine<br>van der Windt, Danielle; Keele University, Primary Care Centre Versus Arthritis, School of Medicine<br>Bucknall, Milica; Keele University, Primary Care Centre Versus Arthritis, School of Medicine

**Primary Subject Heading**:	Rheumatology
Secondary Subject Heading:	Diabetes and endocrinology, Epidemiology
Keywords:	DIABETES & ENDOCRINOLOGY, EPIDEMIOLOGY, PRIMARY CARE, RHEUMATOLOGY

SCHOLARONE™
Manuscripts

I, the Submitting Author has the right to grant and does grant on behalf of all authors of the Work (as defined in the below author licence), an exclusive licence and/or a non-exclusive licence for contributions from authors who are: i) UK Crown employees; ii) where BMJ has agreed a CC-BY licence shall apply, and/or iii) in accordance with the terms applicable for US Federal Government officers or employees acting as part of their official duties; on a worldwide, perpetual, irrevocable, royalty-free basis to BMJ Publishing Group Ltd ("BMJ") its licensees and where the relevant Journal is co-owned by BMJ to the co-owners of the Journal, to publish the Work in this journal and any other BMJ products and to exploit all rights, as set out in our licence.

The Submitting Author accepts and understands that any supply made under these terms is made by BMJ to the Submitting Author unless you are acting as an employee on behalf of your employer or a postgraduate student of an affiliated institution which is paying any applicable article publishing charge ("APC") for Open Access articles. Where the Submitting Author wishes to make the Work available on an Open Access basis (and intends to pay the relevant APC), the terms of reuse of such Open Access shall be governed by a Creative Commons licence – details of these licences and which Creative Commons licence will apply to this Work are set out in our licence referred to above.

Other than as permitted in any relevant BMJ Author's Self Archiving Policies, I confirm this Work has not been accepted for publication elsewhere, is not being considered for publication elsewhere and does not duplicate material already published. I confirm all authors consent to publication of this Work and authorise the granting of this licence.

For peer review only

Title Page

Authors: Brett P Dyer, Trishna Rathod-Mistry, Claire Burton, Danielle A van der Windt, Miliça Blagojevic-Bucknall.

Title: Diabetes as a Risk Factor for the Onset of Frozen Shoulder: A Systematic Review and Meta-Analysis.

Affiliation: Primary Care Centre Versus Arthritis, School of Medicine, Keele University, Staffordshire, UK

Corresponding Author: Brett P Dyer. **Email:** b.p.dyer@keele.ac.uk

ORCID ID's: Brett P Dyer: 0000-0001-8039-7631, Trishna Rathod-Mistry: 0000-0002-6369-4746, Claire Burton 0000-0003-4688-3075, Danielle A van der Windt: 0000-0002-7248-6703, Miliça Blagojevic-Bucknall: 0000-0001-7230-7771.

Word count: 3191 words.

For peer review only

Title

Diabetes as a Risk Factor for the Onset of Frozen Shoulder: A Systematic Review and Meta-Analysis

Abstract

Objective Summarise longitudinal observational studies to determine whether diabetes (types 1 and 2) is a risk factor for frozen shoulder.

Methods Studies were identified through a systematic literature search of eleven bibliographic databases (MEDLINE, EMBASE, AMED, PsycINFO, Web of Science core collection, CINAHL, Epistemonikos, Trip, PEDro, OpenGrey, and The Grey Literature Report; searched on January 2019, and updated in June 2021), reference screening and emailing professional contacts. Longitudinal observational studies that estimated the association between diabetes and developing frozen shoulder were eligible. Risk of bias was judged using the Quality In Prognosis Studies tool. For studies providing sufficient data, random-effects meta-analysis was used to derive summary estimates of the association between diabetes and the onset of frozen shoulder.

Results A meta-analysis of six case-control studies including 5388 people estimated the odds of developing frozen shoulder for people with diabetes to be 3.69 (95% CI: 2.99, 4.56) times the odds for people without diabetes. Two cohort studies were identified, both suggesting diabetes was associated with frozen shoulder, with hazard ratios of 1.32 (95% CI: 1.22, 1.42) and 1.67 (95% CI: 1.46, 1.91). Risk of bias was judged as high in seven studies and moderate in one study.

Conclusions People with diabetes are more likely to develop frozen shoulder. Risk of unmeasured confounding was the main limitation of this systematic review. High-quality studies are needed to confirm the strength of, and understand reasons for, the association.

PROSPERO registration number CRD42019122963.

Funding This work was supported by Versus Arthritis grant number 21899.

Keywords: Diabetes, Frozen shoulder, Adhesive capsulitis, Risk factor, Meta-analysis

Strengths and limitations of this study

- This systematic review is the first to summarise the results of studies estimating the longitudinal association between diabetes and the onset of frozen shoulder.
- Robust meta-analytic methods were used to synthesise and analyse data.
- Sensitivity to influential estimates and sensitivity to small study bias were assessed.
- Risk of bias was judged to be high in seven studies and moderate in one study; this limits the certainty in evidence.
- Only two cohort studies were identified which meant that pooling of association estimates was not suitable.

For peer review only

1 - Introduction

Frozen shoulder, also known as adhesive capsulitis, is a painful and severely debilitating condition. The inflammatory contracture of the glenohumeral joint capsule in frozen shoulder restricts both active and passive range of motion, with loss of external rotation being especially characteristic of this condition [1].

Frozen shoulder generally presents between the ages of 50 and 60 years and rarely presents before 40 years [2]. Women (58%) are more likely to develop frozen shoulder than men (42%) [3]. The contralateral shoulder is also affected in 6% to 17% of patients [4]. Although the exact aetiology remains unclear, several factors have been found to be associated with frozen shoulder, including trauma [3], thyroid dysfunction [5-7], cardiovascular disease [2,8], metabolic factors [7,9-11], and other musculoskeletal conditions such as Dupuytren's contracture [12,13]. The most common comorbidity in people with frozen shoulder is diabetes [2], both type 1 and type 2 [6]. The prevalence of frozen shoulder in the general population is around 0.75% [1], but the prevalence of frozen shoulder in people with diabetes is much higher. A meta-analysis of 13 cross-sectional studies estimated the prevalence of frozen shoulder in populations with diabetes to be 13.4% (95% confidence interval (CI): 10.2%, 17.2%) [14].

Diabetes is a term used to describe a group of chronic diseases characterised by hyperglycaemia. The two most prevalent types of diabetes are type 1 and type 2, making up 8% and 90% of cases, respectively [15]. It is well-known that people with diabetes are at risk of complications such as cardiovascular disease, retinopathy, neuropathy, and nephropathy [16], although the musculoskeletal complications of diabetes are not as well-known [17]. Musculoskeletal conditions, such as frozen shoulder, can significantly affect the quality of a patient's life and should not be overlooked. Our previous systematic review and narrative synthesis of 28 studies has shown that patients with diabetes may experience worse outcomes from frozen shoulder than people without frozen shoulder [18].

It has been suggested that diabetes may be a cause of frozen shoulder through glycation processes and/or inflammatory processes leading to capsular fibrosis and subsequent contracture [7,19,20]. To understand whether diabetes could potentially be a cause of frozen shoulder it is necessary (although not sufficient) to have evidence of the temporal relationship between diabetes and frozen shoulder [21]. This systematic review aims to summarise evidence from longitudinal observational studies to understand the temporal relationship between diabetes and frozen shoulder.

2 - Methods

2.1 – Search Strategy

The protocol for this systematic review was registered on PROSPERO (CRD42019122963) and the review was conducted and reported using PRISMA guidelines [22]. A systematic literature search of MEDLINE, EMBASE, AMED, PsycINFO, Web of Science core collection, CINAHL, Epistemonikos, Trip, PEDro, OpenGrey, and The Grey Literature Report was carried out in January 2019 and updated in June 2021. Reference lists of eligible studies were screened. Additionally, a professional contact of one author (DAvdW) was contacted to identify further studies. We retrieved all epidemiological studies containing index terms (e.g. Medical Subject Headings) and free-text words related to diabetes and shoulder pain more generally (not limited to frozen shoulder) to reduce the risk of missing potentially relevant publications. The search strategy for MEDLINE, which was constructed with the support of a health information specialist, can be found in Appendix A.

2.2 – Study Selection

Reviewer BPD screened all titles and abstracts to check eligibility using the pre-defined inclusion and exclusion criteria, and reviewers MB-B and CB independently checked a 20% random sample. Reviewer BPD checked all full-texts for eligibility using the inclusion and exclusion criteria, and reviewers MB-B, CB, TR-M also

For peer review only - http://bmjopen.bmj.com/site/about/guidelines.xhtml

independently checked eligibility. Disagreements regarding the inclusion of studies were resolved through discussion with DAvdW.

2.3 – Inclusion Criteria

To be eligible for inclusion, studies were required to have a longitudinal, prospective or retrospective, observational study design. Cohort studies were required to have a study population consisting of people without frozen shoulder at inclusion and must have established whether diabetes was present at baseline (all types of diabetes were considered). Case-control studies were required to have a study population consisting of people with frozen shoulder and a control group without frozen shoulder, with diabetes defined as the exposure of interest. The paper must have presented an odds ratio, risk ratio or hazard ratio, or they must have presented sufficient data to allow the associations to be estimated. There were no restrictions to setting; population based as well as clinical cohorts were eligible. All non-English language papers were assessed by reviewers with appropriate language skills. Cross-sectional studies and case series were excluded. Studies were also excluded if a full text could not be obtained.

2.4 – Data Extraction and Risk of Bias

Data extraction was completed by reviewer BPD and was independently checked by reviewers MB-B and TR-M. Types of data extracted included details of study design, setting, sample characteristics, exposure/outcome/covariate measurement, inclusion and exclusion criteria, sample size, attrition, covariate conditioning, follow-up time, statistical analysis, association estimates (odds ratio, risk ratio or hazard ratio) or raw data to estimate association sizes if they were not already presented. Risk of bias was independently assessed by pairs of reviewers (BPD, MB-B, TR-M). Risk of bias was judged using the Quality In Prognosis Studies (QUIPS) tool [23]. The QUIPS tool covers six domains: (1) study participation, (2) study attrition, (3) prognostic/risk factor measurement, (4) outcome measurement, (5) study confounding, (6) statistical analysis and reporting. Each of the six domains is scored as being at a low, medium, or high risk of bias [23]. Domain scores were used to guide judgement of the overall risk of bias (scored as low, medium or high) for the study. Overall risk of bias was based on author judgement and the use of a tallied or summated score was avoided. All disagreements regarding data extraction and assessment of risk of bias were resolved by discussion.

2.5 – Data Analysis

Case-control studies and cohort studies were analysed separately. Narrative synthesis was used where less than five studies were present and a random-effects meta-analysis model was used to estimate a summary estimate when five or more studies were present. Cohort study associations were measured using hazard ratios and case-control study associations were estimated using odds ratios. Where adjusted and crude estimates were both presented, the adjusted estimate was used. Where a zero-cell count was present within the results of a study, a continuity correction of 0.5 was added to all cells for that study. Restricted maximum likelihood estimation [24] was used to estimate the between-study variance, τ^2, and the Hartung-Knapp-Sidik-Jonkman variance correction method [25] was used in the estimation of the pooled effect confidence interval. Heterogeneity was assessed using Cochran's Q statistic, complemented by the I^2 index [26]. Prediction intervals were not estimated since they are inaccurate when there is little heterogeneity ($I^2 < 0.3$), or an imbalance in study sizes exists, both of which were found in the meta-analysis in this review (see Section 3) [27]. A forest plot was used to visualise results of individual results and of the pooled estimate. Evidence of small-study bias was assessed with a funnel plot of log odds ratios against their standard errors [28]. A test for funnel plot asymmetry was not used since the meta-analysis included less than ten studies [29]. The influence of each study on the overall pooled estimate was assessed by repeating the meta-analysis, each time leaving out a single study [30]. Statistical analysis was carried out using Stata version 16.1 [31].

2.6 - Patient and Public Involvement

No patient involved.

For peer review only

3 - Results

The searches identified 1784 unique citations, 12 of which were selected for full-text screening, and eight studies consisting of a total of 346,278 people fulfilled the inclusion criteria (Figure 1). Table 1 summarises information on risk of bias, study design, setting, participants, sample size, and methods used for diagnosing diabetes and frozen shoulder. Of the eight studies that met the criteria for inclusion, six [32-37] had case-control designs and two [38,39] had cohort designs. Three studies [37-39] (including the two cohort studies)

Table 1. Characteristics of Studies on diabetes as a risk factor for frozen shoulder

Source	Risk of Bias (QUIPS, overall assess-ment)	Design and Setting	% Female	Mean Age (years)	Sample Size	Method to diagnose diabetes and frozen shoulder	Variables conditioned on
**Case-control studies**							
K. L. Boyle-Walker, et al., 1997 [32]	High	Sex-Matched Case-Control at Physical Therapy Clinic in the USA	Case Group: 75%, Control Group: 68%	Not reported	Cases: 32, Controls: 31	Diabetes: Self-reported Questionnaire Frozen shoulder: Clinically diagnosed	Sex-matched
W. Li, et al., 2014 [33]	High	Hospital based case-control matched on time of hospitalisation in China	Case Group: 63%, Control Group: 55%	Cases: 57.2, Controls: 45.9	Cases: 182, Controls: 196	Diabetes: Face-to-face interview Frozen shoulder: Clinically diagnosed	Matched on time of hospitalisation, adjusted for history of minor shoulder trauma
S-Y. Lee, et al., 2012 [34]	High	Hospital based age- and sex-matched case-control in South Korea	Case Group: 55%, Control Group: not reported	Cases: 52.8, Controls: not reported	Cases: 40, Controls: 40	Diabetes: Unclear Frozen shoulder: Clinically diagnosed	Age- and sex-matched
C. Milgrom, et al., 2008 [35]	High	Hospital based age-matched case-control in Israel	Case Group: 60%, Control Group: 65%	Cases: 54.9, Controls: 55.4	Cases: 126, Controls: 98	Diabetes: If patient was receiving drug treatment for Diabetes or whose serum glucose was higher than 200 mg/dl Frozen shoulder: Clinically diagnosed	Age-matched

collected information from electronic health records, four studies [33-36] were hospital-based, and one study [32] was based in a physical therapy clinic. Among the case-control studies, the percentage of female cases ranged from 52% to 75% and the mean age for cases ranged from 52.8 years to 57.2 years.

For peer review only

K. Wang, et al., 2013 [36]	High	Hospital based age- and sex-matched case-control in Australia	Case Group: 64%, Control Group: 58%	Cases: 56, Controls: 55.3	Cases: 87, Controls: 176	Diabetes: Self-reported Frozen shoulder: Clinically diagnosed	Age- and sex-matched
K. Kingston, et al., 2018 [37]	High	Sex-matched case-control using electronic health records in the USA	Case Group: 58%, Control Group: 58%	Cases: 56.4, Controls: Not Reported	Cases: 2190, Controls: 2190	Diabetes: ICD-9 Code Frozen shoulder: ICD-9 Code	Sex-matched
**Cohort studies**							
Y-P. Huang, et al., 2013 [38]	High	Age- and sex-matched cohort with 3-year follow-up using electronic health records in Taiwan	Exposed Group: 47%, Non-Exposed Group: 47%	Exposed Group: 55.7, Non-Exposed Group: 55.5	Exposed Group: 78,827, Non-Exposed Group: 236,481	Diabetes: ICD-9 Code Frozen shoulder: ICD-9 Code	Age- and sex-matched. Multivariable analysis adjusted for age, sex, dyslipidaemia
S-F. Lo, et al., 2013 [39]	Moderate	Cohort with 8-year follow-up using electronic health records in Taiwan	Exposed Group: 52%, Non-Exposed Group: 51%	Not reported	Exposed Group: 5109, Non-Exposed Group: 20,473	Diabetes: ICD-9 Code Frozen shoulder: ICD-9 Code	Multivariable analysis adjusted for age, income, stroke, hypertension, hyperlipidaemia, obesity, chronic obstructive pulmonary disease

Presence of diabetes was identified using ICD-9 codes (codes to classify diseases, symptoms, clinical findings and causes of disease and injury) from electronic health records in three studies [37-39], self-reported in three studies [32,33,36], identified with a glucose test or if the patient was receiving drug treatment for diabetes in one study [35], and was unclear in one study [34]. Frozen shoulder was identified using [37-39] ICD-9 codes in three studies and was diagnosed clinically in five studies [32-36]. Only one study [39] reported the types of diabetes that the participants had. Lo et al. [39] stated that 296 (5.8%) of the 5109 people with diabetes in their study had type 1 diabetes. Two studies were conducted in Taiwan [38,39], two in the USA [32,37] and the remaining four were conducted in China [33], South Korea [34], Israel [35] and Australia [36].

Overall QUIPS risk of bias scores for each study can be found in Table 1 and full QUIPS assessments can be found in Table 2. Overall, there was a 75% agreement between reviewers across the individual bias domains, and reviewers agreed on 4 of the 8 overall risk of bias scores. One of the cohort studies [39] was scored as being at a moderate risk of bias for their overall study rating and the other seven studies were rated as being at a high risk of bias overall. A bar graph of the scores for individual risk of bias domains can be found in Appendix Figure B1. Risk of bias was generally high across most domains, but especially so for the risk of unaccounted confounding, which was scored as being at a high risk of bias in all eight studies. Five of the case-control studies [32,34-37] only accounted for age, gender or a combination of the two. One study [33] matched on the time of hospitalisation and adjusted for history of minor shoulder trauma. One cohort study [38] adjusted for age, sex and dyslipidaemia; the other cohort study [39] adjusted for age, income, stroke, hypertension, hyperlipidaemia, obesity and chronic obstructive pulmonary disease.

For peer review only

Table 2 QUIPS domain scores for each primary study

Source	Participation	Study Attrition	Risk Factor Measure-ment	Outcome Measure-ment	Confounding	Statistical Analysis and Presentation	Overall Risk of Bias
**Case-Control Studies**							
K. L. Boyle-Walker, et al., 1997 [32]	High	Moderate	High	Moderate	High	Moderate	High
W. Li, et al., 2014 [33]	Moderate	Low	Moderate	High	High	High	High
S-Y. Lee, et al., 2012 [34]	Moderate	Low	Moderate	Moderate	High	Moderate	High
C. Milgrom, et al., 2008 [35]	Moderate	Low	Low	Low	High	Low	High
K. Wang, et al., 2013 [36]	Low	Low	Low	Low	High	Low	High
K. Kingston, et al., 2018 [37]	Low	Moderate	Moderate	Low	High	Moderate	High
**Cohort studies**							
Y-P. Huang, et al., 2013 [38]	Low	Moderate	Low	High	High	High	High
S-F. Lo, et al., 2013 [39]	Low	Low	Low	Moderate	High	Low	Moderate

Six case-control studies including a total of 5388 people were pooled in a random-effects meta-analysis, with a pooled odds ratio of 3.69 (95% CI: 2.99, 4.56) (Figure 2). The raw data extracted from each study that was used to calculate odds ratios can be found in Appendix Table C1. The estimated between-study variance was small (τ^2<0.01, 95% CI: <0.01, 0.23) and little heterogeneity was detected (Q=2.07, df=5, p=0.84; I^2 <0.01% (95% CI: <0.1%, 67.6%)), but the estimate for I^2 was imprecise as indicated by the wide 95% confidence interval. The influence analysis showed that excluding the largest study [37], which contained 4380 of the 5388 participants, greatly reduced the precision of the pooled estimate but did not substantially affect the value of the pooled estimate (Figure 3). Further, excluding any other single study did not substantially affect the value of the pooled estimate (Figure 3). The two studies with the smallest standard errors for their effect estimates had the largest odds ratio's, making the funnel plot appear unsymmetrical. However, due to the small number of studies contributing to the funnel plot, the asymmetrical appearance could be due to chance (Figure 4).

The two cohort studies that were identified used Cox proportional-hazards models and obtained results suggesting that people with diabetes were more at risk of developing frozen shoulder. One cohort study [38] using electronic health records from Taiwan, with a 3-year follow-up and consisting of 315,308 people reported an age-, sex- and dyslipidaemia-adjusted hazard ratio of 1.32 (95% CI: 1.22, 1.42). Another cohort study [39], with an 8-year follow-up, consisting of 25,582 people, also using electronic health records from Taiwan, estimated an age-, income-, stroke-, hypertension-, hyperlipidaemia-, obesity- and chronic obstructive pulmonary disease- adjusted hazard ratio of 1.67 (95% CI: 1.46, 1.91).

4 - Discussion

This systematic review consists of eight studies each of which demonstrated evidence to suggest that diabetes is associated with the onset of frozen shoulder. Our meta-analysis of six case-control studies yielded a pooled odds ratio of 3.69 (95% CI: 2.99, 4.56), and the value of the pooled estimate was robust to the omission of any individual study. The odds ratio estimates of all but one study [37] were imprecise with large confidence intervals; this meant that the confidence intervals overlapped well, resulting in a small I^2 value. It is also

For peer review only

important to note that Cochran's Q statistic should be interpreted with caution since the number of studies included in the analysis was small [40].

The funnel plot was unsymmetrical. However, given that a small number of studies were available, it was difficult to assess accurately whether any small-study bias was present or if the appearance was due to chance. Since our influence analysis has shown that the inclusion/exclusion of any individual study had very little impact on the pooled effect estimate, any potential small-study bias would be unlikely to substantially affect the results.

Two cohort studies were identified, both of which corroborate the evidence from the six case-control studies reported above, that people with diabetes are more likely to develop frozen shoulder than those without diabetes. Of the two cohort studies, one was deemed to be at a high risk of bias and the other at a moderate risk of bias. The hazard ratios in the two studies did differ, which could have partly been due to the differences in the covariates that were adjusted for and/or the differences in the duration of follow-up. Both studies were rated as being at a high of bias for the outcome-measurement domain as the length of follow-up (3 years [38] and 8 years [39]) was deemed too short to establish whether a patient would develop frozen shoulder in the future. Previous studies have suggested that the duration of diabetes may be associated with the risk of developing frozen shoulder [41,42], with one of the cohort studies in this review also stating that their study suggested that "the development of [frozen shoulder] is associated with the duration of diabetes" [38]. Therefore, future studies should ensure that the follow-up period is long enough to observe participants from diabetes diagnosis through to the ages for which frozen shoulder is common. A cross-sectional study of 1,373 patients presenting with frozen shoulder estimated that the mean age of onset for frozen shoulder was 55.4 years with a standard deviation of 9.9 years [3].

The two cohort studies in the review were both conducted using Electronic Health Records (EHRs). EHR datasets can provide large sample sizes with long follow-up periods and detailed patient medical record history [43]. Misdiagnosis and miscoding in EHRs are common limitations and could potentially result in a risk of bias for frozen shoulder measurement [44]. Research in the UK [45] and in the Netherlands [46] has shown that general practitioners often use non-specific shoulder pain codes instead of codes for specific shoulder conditions, e.g., frozen shoulder. This would lead to an underdiagnosis of frozen shoulder. Further, this misclassification may be differential since clinicians may feel more confident in providing a specific frozen shoulder diagnosis in patients with diabetes due to the pre-existing knowledge of the association between the two conditions. Conversely, it has also been noted that frozen shoulder is sometimes used as a "waste-bin diagnosis" for patients presenting with any stiff and painful shoulder [47]. Thus, EHR data may include other shoulder conditions with similar clinical presentations being coded as frozen shoulder.

Another important limitation was the overall poor adjustment for confounding variables. All eight studies were rated as being at a high risk of unaccounted confounding. In each study, confounders were either ignored [32,34-38] or inappropriate statistical methods, such as univariable prefiltering and stepwise selection, were used [33,38,39]. These methods are especially poorly suited for aetiologic models [48]. Thus, these studies may have missed potentially important confounders [33,38,39] or erroneously adjusted for mediators, such as stroke [39].

The systematic review is also limited by there being only two cohort studies, meaning that pooling association estimates was not possible. Cohort studies are particularly useful for gaining a better understanding of temporal associations, as this review aimed to do. Further, both cohort studies were conducted in Taiwan using existing data from EHRs. Future studies with prospective designs will help to gauge whether the findings of these two cohort studies are reproducible, and whether the results are consistent across different populations.

Previously, a meta-analysis of cross-sectional studies established that frozen shoulder was more prevalent in people with diabetes than among people without diabetes. This systematic review provides evidence of a temporal relationship between diabetes and frozen shoulder. Understanding the temporal relationship is key to explaining why diabetes and frozen shoulder are associated, however further high-quality

For peer review only

research with appropriate methods and study design is required to confirm the strength of the association and establish whether diabetes is indeed a cause of frozen shoulder.

Whilst sound and reliable epidemiological evidence of a causal relationship between diabetes and frozen shoulder is currently unavailable, elsewhere in the literature, researchers have hypothesised about potential pathological mechanisms through which diabetes may lead to frozen shoulder. Current evidence, based on histological studies, suggests that a pathophysiological process consisting of chronic inflammation and capsular fibrosis leads to the contracture in frozen shoulder [49,50]. It has been hypothesised that the accumulation of advanced glycation end products (AGE's), which lead to the cross-linking of collagen [51,52], may explain the fibrosis in the capsule of frozen shoulder patients [33]. Glycation is a process by which simple sugars bond to proteins, which is enhanced by persistent hyperglycaemia. Thus, the role of glycation and AGEs in the fibrosis of the shoulder capsule could potentially be a reason why diabetes is associated with frozen shoulder. Another potential reason why diabetes may be associated with frozen shoulder is that hyperglycaemia may induce proinflammatory cytokines [53] which have been found to be elevated in the capsule and synovium of frozen shoulder patients [54].

The association between glycaemic control and the risk of developing frozen shoulder should also be a focus for future research. One study found evidence to suggest that poor long-term glycaemic control in people with diabetes is associated with an increased incidence of frozen shoulder [55], whilst another study found no association between HbA1c level in people with diabetes and the prevalence of frozen shoulder [56]. Further research is required to investigate whether glycaemic control is associated with the development of frozen shoulder.

5 - Conclusion

In summary, people with diabetes are more at risk of developing frozen shoulder than people without diabetes. However, existing research is limited by the high risk of unmeasured confounding. To better understand the nature of the relationship between diabetes and the onset of frozen shoulder, it is necessary to have high-quality cohort studies that use causal inference methods that are appropriate for aetiologic modelling. Given the existing evidence that has been summarised in this review, clinicians should consider checking whether patients with diabetes are experiencing shoulder pain at their routine follow-up appointments. An early diagnosis will help the clinician to provide treatment for the pain and lack of function that result from frozen shoulder.

6 - Acknowledgements

This study was supported by the Versus Arthritis PhD scholarship scheme [grant number 21899]. The author would also like to thank the information specialists of evidence synthesis team at the Primary Care Centre Versus Arthritis for their advice regarding the search strategy, and Elaine Willmore for taking time to check the results of our searches for this review. Further thanks are given to Dr Linda Chesterton, who was involved in the initial conceptualisation of this study. Claire Burton is funded by an NIHR (National Institute for Health Research) Clinical Lectureship. The views expressed are those of the authors and not necessarily those of the National Health Service, the NIHR, or the Department of Health and Social Care.

Abbreviations

QUIPS - Quality In Prognosis Studies

CI – Confidence interval

Q – Cochran's Q statistic

For peer review only

df – Degrees of freedom

Ethics Approval

No Ethics Committee or Institutional Board approval is required.

No human participants included.

Author contributions

All authors (BPD, CB, DvdW, MB-B and TR-M) contributed to the conception of the study and systematic review study selection. Data extraction and risk of bias assessment was performed by BPD, MB-B and TR-M. BPD performed the meta-analysis and narrative synthesis of the results and drafted the initial manuscript. All authors (BPD, CB, DvdW, MB-B and TR-M) contributed to editing and approval of the final manuscript.

Funding

This work was supported by Versus Arthritis grant number 21899. Claire Burton is funded by an NIHR (National Institute for Health Research) Clinical Lectureship. The views expressed are those of the authors and not necessarily those of the National Health Service, the NIHR, or the Department of Health and Social Care.

Data availability

Data have been included in Table 1 and Appendix Table C.1.

Conflicts of interest statement

The authors have no conflicts of interest.

Supplementary Material

Appendix A contains the search strategy for MEDLINE.

Appendix B contains full QUIPS risk of bias assessments for each primary study and a bar graph of QUIPS scores for each of the six bias domains.

Appendix C contains the raw data extracted from each primary study that was used to calculate the odds ratios included in the meta-analysis.

References

[1] Bunker T. Frozen Shoulder. *Orthopaedics and Trauma*. 2011; 25(1):11-8. https://doi.org/10.1016/j.mporth.2011.01.007

[2] Dias R, Cutts S, Massoud S. Frozen shoulder. *BMJ*. 2005; 331:1453-6. https://doi.org/10.1136/bmj.331.7530.1453

For peer review only

[3] Cho C-H, Koo TW, Cho N-S, Park K-J, Lee BG, Shin D, Choi S, Cho S-H, Kim M-S, Ko S-H, Kim C-H, Park J-Y, Yoo Y-S. Demographic and Clinical Characteristics of Primary Frozen Shoulder in a Korean Population. *Clin Shoulder Elbow*. 2015; 18(3):133-7. doi: 10.5397/cise.2015.18.3.133

[4] Rizk TE, Pinals RS. Frozen shoulder. *Seminars in Arthritis and Rheumatism.* 1982; 11(4):440-52. doi: 10.1016/0049-0172(82)90030-0

[5] Cohen C, Tortato T, Silva OBS, Ferreira Leal M, Ejnisman B, Faloppa F. Association between frozen shoulder and thyroid diseases: strengthening the evidences. *Rev Bras Ortop.* 2020; 55(4):483–9. doi: 10.1055/s-0039-3402476

[6] Huang S-W, Lin J-W, Wang W-T, Wu C-W, Lious T-H, Lin H-W. Hyperthyroidism is a risk factor for developing adhesive capsulitis of the shoulder: a nationwide longitudinal population-based study. *Sci Rep*. 2014; 25(4):4183. doi: 10.1038/srep04183

[7] Cucchi D, Marmotti A, De Giorgi S, Costa A, D'Apolito R, Conca M, Russo A, Saccomanno MF, de Girolamo L. Risk Factors for Shoulder Stiffness: Current Concept. *Joints*. 2017; 5(4):217-23. doi: 10.1055/s-0037-1608951

[8] Nagy MT, MacFarlane RJ, Khan Y, Waseem M. The frozen shoulder: myths and realities. *Open Orthop J*, 2013; 7:352-355. doi: 10.2174/1874325001307010352

[9] Sung CM, Sung TS, Park HB. Are serum lipids involved in primary frozen shoulder? A case-control study. *J Bone Joint Surg*. 2014; 96(21):1828–33. doi: 10.2106/JBJS.M.00936

[10] Nayak SP, Panda CK. Is hyperlipidaemia a cause of primary frozen shoulder? A case-controlled study. *J. Evid based med. healthc.* 2017; 4(13):697-710. doi 10.18410/jebmh/2017/135

[11] Austin D, Gans I, Park M, Carey J, Kelly J. The association of metabolic syndrome markers with adhesive capsulitis. *J Shoulder and Elbow Surg.* 2014; 23(7):1043-51. doi: 10.1016/j.jse.2013.11.004

[12] Smith SP, Devaraj VS, Bunker TD. The association between frozen shoulder and Dupuytren's disease. *J Shoulder Elbow Surg*. 2001; 10(2):149-51. doi: 10.1067/mse.2001.112883.

[13] Bunker TD, Anthony PP. The pathology of frozen shoulder. A Dupuytren-like disease. *J Bone Joint Surg Br.* 1995; 77(5):677-83. PMID: 7559688.

[14] Zreik NH, Malik RA, Charalambos CP. Adhesive capsulitis of the shoulder and diabetes: a meta-analysis of prevalence. *MLTJ.* 2016; 6(1):26-34. doi: 10.11138/mltj/2016.6.1.026

[15] NHS Digital. *National Diabetes Audit 2017/18 - Report 1 Care Processes and Treatment Targets.* 2020. Accessed: 8th September 2022 https://digital.nhs.uk/data-and-information/publications/statistical/national-diabetes-audit/report-1-care-processes-and-treatment-targets-2017-18-short-report

[16] Fowler MJ. Microvascular and Macrovascular Complications of Diabetes. *Clin Diabetes*. 2008; 26(2):77–82. https://doi.org/10.2337/diaclin.26.2.77

[17] Sözen T, Başaran NC, Tınazlı M, Özışık L. Musculoskeletal problems in diabetes mellitus. Eur J Rheumatol, 2018; 5(4):258–65. doi: 10.5152/eurjrheum.2018.18044

[18] Dyer BP, Burton C, Rathod-Mistry T, Blagojevic-Bucknall M, van der Windt DA. Diabetes as a prognostic factor in frozen shoulder: a systematic review. Arch Rehabil Res Clin Transl. 2021; 3:100141. https://doi.org/10.1016/j.arrct.2021.100141

[19] Hsu C-L, Sheu WH-H. Diabetes and shoulder disorders. *J Diabetes Investig*. 2016; 7(5):649-51. https://doi.org/10.1111/jdi.12491

[20] Pietrzak M, Adhesive capsulitis: An age-related symptom of metabolic syndrome and chronic low-grade inflammation? Med Hypotheses. 2016; 88:12-7. doi: 10.1016/j.mehy.2016.01.002.

[21] Rothman KJ, Greenland S, Lash TL. *Modern Epidemiology*: 3rd ed. Lippincott Williams & Wilkins. 2013.

For peer review only

[22] Moher D, Liberati A, Tetzlaff J, Altman DG. Preferred reporting items for systematic reviews and meta-analyses: the PRISMA statement. *BMJ.* 2009; 339:b2535. doi: 10.1136/bmj.b2535

[23] Hayden JA, van der Windt DA, Cartwright JL, Côté P, Bombardier C. Assessing bias in studies of prognostic factors. *Ann Intern Med*. 2019; 158(4):280–6. doi: 10.7326/0003-4819-158-4-201302190-00009

[24] Veroniki AA, Jackson D, Viechtbauer W, Bender R, Bowden J, Knapp G, Kuss O, Higgins JPT, Langan D, Salanti G. Methods to estimate the between-study variance and its uncertainty in meta-analysis. *Res Synth Meth. 2016;* 7(1):55-79. doi: 10.1002/jrsm.1164

[25] IntHout J, Ioannidis JPA, Borm GF. The Hartung-Knapp-Sidik-Jonkman method for random-effects meta-analysis is straightforward and considerably outperforms the standard Der-Simonian-Laird method. *BMC Med Res Methodol*. 2014; 14:25. https://doi.org/10.1186/1471-2288-14-25

[26] Higgins JPT, Thompson SG, Deeks JJ, Altman DG. Measuring inconsistency in meta-analyses. *BMJ.* 2003; 327:557-60. https://doi.org/10.1136/bmj.327.7414.557

[27] Partlett C, Riley RD. Random effects meta-analysis: Coverage performance of 95% confidence and prediction intervals following REML estimation. *Statist. Med*. 2016; 36(2):301-17. https://doi.org/10.1002/sim.7140

[28] Sterne JAC, Harbord RM. Funnel Plots in Meta-analysis. *Stata Journal*. 2004; 4(2):127-41. https://doi.org/10.1177/1536867X0400400204

[29] Higgins JPT, Green S. Cochrane Handbook for Systematic Reviews of Interventions Version 5.1.0 [updated March 2011]. *The Cochrane Collaboration*. 2011.

[30] Viechtbauer W, Cheung MWL. Outlier and influence diagnostics for meta-analysis. *Res Syn Meth. 2010;* 1(2):112-25. doi: 10.1002/jrsm.11

[31] StataCorp. 2019. *Stata Statistical Software: Release 16.* College Station, TX: StataCorp LLC.

[32] Boyle-Walker KL, Gabard DL, Bietsch E, Masek-VanArsdale DM, Robinson BL. A profile of patients with adhesive capsulitis. *J Hand Ther*. 1997; 10(3):222-8. https://doi.org/10.1016/S0894-1130(97)80025-7

[33] Li W, Lu N, Xu H, Wang H, Huang J. Case control study of risk factors for frozen shoulder in China. *Int J Rheum Dis*. 2014; 18(5):508-13. https://doi.org/10.1111/1756-185X.12246

[34] Lee S-Y, Park J, Song S-W. Correlation of MR Arthrographic Findings and Range of Shoulder Motions in Patients With Frozen Shoulder. *American Journal of Roentgenology*. 2012; 198(1):173-9. doi: 10.2214/AJR.10.6173

[35] Milgrom C, Novack V, Weil Y, Jaber S, Radeva-Petrova DR, Finestone A. Risk factors for idiopathic frozen shoulder. *Isr Med Assoc J*. 2008; 10(5):361-4. PMID: 18605360

[36] Wang K, Ho V, Hunter-Smith DJ, Beh PS, Smith KM, Weber AB. Risk factors in idiopathic adhesive capsulitis: a case control study. *J Shoulder Elbow Surg*. 2013; 22(7):24-9. doi: 10.11138/mltj/2016.6.1.026

[37] Kingston K, Curry EJ, Galvin JW, Li X. Shoulder adhesive capsulitis: epidemiology and predictors of surgery. *J Shoulder Elbow Surg*. 2018; 27(8):1437-43. doi: 10.1016/j.jse.2018.04.004

[38] Huang Y-P, Fann C-Y, Chiu Y-H, Yen M-F, Chen L-S, Chen H-H, Pan S-L. Association of Diabetes Mellitus With the Risk of Developing Adhesive Capsulitis of the Shoulder: A Longitudinal Population-Based Follow up Study. *Arthritis Care & Research*. 2013; 65(7):1197-202. https://doi.org/10.1002/acr.21938

[39] Lo S-F, Chu S-W, Muo C-H, Meng N-H, Chou L-W, Huang W-C, Huang C-M, Sung F-C. Diabetes mellitus and accompanying hyperlipidemia are independent risk factors for adhesive capsulitis: a nationwide population-based cohort study. *Rheumatology International*. 2014; 34:67-74. doi: 10.1007/s00296-013-2847-4

[40] Hardy RJ, Thompson SG. Detecting and describing heterogeneity in meta-analysis. *Stat Med*. 1998; 17(8):841-56. doi: 10.1002/(sici)1097-0258(19980430)17:8<841::aid-sim781>3.0.co;2-d

[41] Thomas SJ, McDougall C, Brown IDM, Jaberoo M-C, Stearns A, Ashraf R, Fisher M, Kelly IG. Prevalence of symptoms and signs of shoulder problems in people with diabetes mellitus. *J Shoulder and Elbow Surg.* 16(6); 748-51. doi: 10.1016/j.jse.2007.02.133

[42] Bridgeman JF, Periarthritis of the shoulder and diabetes mellitus. *Ann Rheum Dis.* 31: 69-71; 1972. doi: 10.1136/ard.31.1.69

[43] Herrett E, Gallagher AM, Bhaskaran K, Forbes H, Mathur R, van Staa T, Smeeth L. Data Resource Profile: Clinical Practice Research Datalink (CPRD). *Int J Epidemiol*. 2015; 44(3):827-36. doi: 10.1093/ije/dyv098.

[44] Young JC, Conover MM, Jonsson Funk M. Measurement error and misclassification in electronic medical records: methods to mitigate bias. *Curr Epidemiol Rep*. 2018; 5(4): 343–356. doi: 10.1007/s40471-018-0164-x

[45] Linsell L, Dawson J, Zondervan K, Rose P, Randall T, Fitzpatrick R, Carr A. Prevalence and incidence of adults consulting for shoulder conditions in UK primary care; patterns of diagnosis and referral. *Rheumat* 2006; 45(2):215-21.

[46] Dorrestijn O, Greving K. Patients with shoulder complaints in general practice: consumption of medical care. *Rheumatol*. 2011; 50(2): 389-95.

[47] Bunker T Time for a new name for frozen shoulder – contracture of the shoulder. *Shoulder Elbow.* 2009; 1(1):4-9. https://doi.org/10.1111/j.1758-5740.2009.00007.x

[48] Heinze G, Dunkler D. Five myths about variable selection. *Transplant International*. 2017; 30(1):6-10. https://doi.org/10.1111/tri.12895

[49] Wong PL, Tan HC. A Review on Frozen Shoulder. *Singapore Med J*. 2010; 51(9):694-7. PMID: 20938608

[50] Cho C-H, Song K-S, Kim B-S, Kim D.H, Lho Y-M. Biological Aspect of Pathophysiology for Frozen Shoulder. *BioMed Research International*. 2018; Article ID 7274517. https://doi.org/10.1155/2018/7274517

[51] Hwang KR, Murrell GAC, Millar NL, Bonar F, Lam R, Walton JR. Advanced glycation end products in idiopathic frozen shoulders. *J Shoulder and Elbow Surg.* 2016; 25(6): 981-8. doi: 10.1016/j.jse.2015.10.015

[52] Goldin A, Beckman JA, Schmidt AM, Creager MA. Advanced Glycation End Products. *Circulation*. 2006; 114(6): 597-605. doi: 10.1161/CIRCULATIONAHA.106.621854.

[53] Shanmugam N, Reddy MA, Guha M, Natarajan R. High glucose-induced expression of proinflammatory cytokine and chemokine genes in monocytic cells. *Diabetes*. 2003; 52(5):1256-64. doi: 10.2337/diabetes.52.5.1256

[54] Lho Y-M, Ha E, Cho C-H, Song K-S, Min B-W, Bae K-C, Lee K-J, Hwang I, Park H-B. Inflammatory cytokines are overexpressed in the subacromial bursa of frozen shoulder. *J Shoulder and Elbow Surg.* 2013; 22(5): 666-72. https://doi.org/10.1016/j.jse.2012.06.014

[55] Chan JH, Ho BS, Alvi HM, Saltzman MD, Marra G. The relationship between the incidence of adhesive capsulitis and hemoglobin A1c. *J Shoulder and Elbow Surg.* 2017; 26(10):1834-7. https://doi.org/10.1016/j.jse.2017.03.015

[56] Yian EH, Contreras R, Sodl JF. Effects of Glycemic Control on Prevalence of Diabetic Frozen Shoulder. *J Bone Joint Surg.* 2012; 94(10):919-23. doi: 10.2106/JBJS.J.01930

Figure Captions

Fig. 1 PRISMA flow diagram summarising record identification and study selection.

For peer review only

Fig. 2 Random effects meta-analysis forest plot of the association between diabetes and the odds of developing frozen shoulder.

Fig. 3 Influence plot showing the result of repeating the original meta-analysis (Figure 2), each time with a different primary study removed.

Fig. 4 Funnel plot of log odds ratios for developing frozen shoulder in people with diabetes vs. those without diabetes.

For peer review only

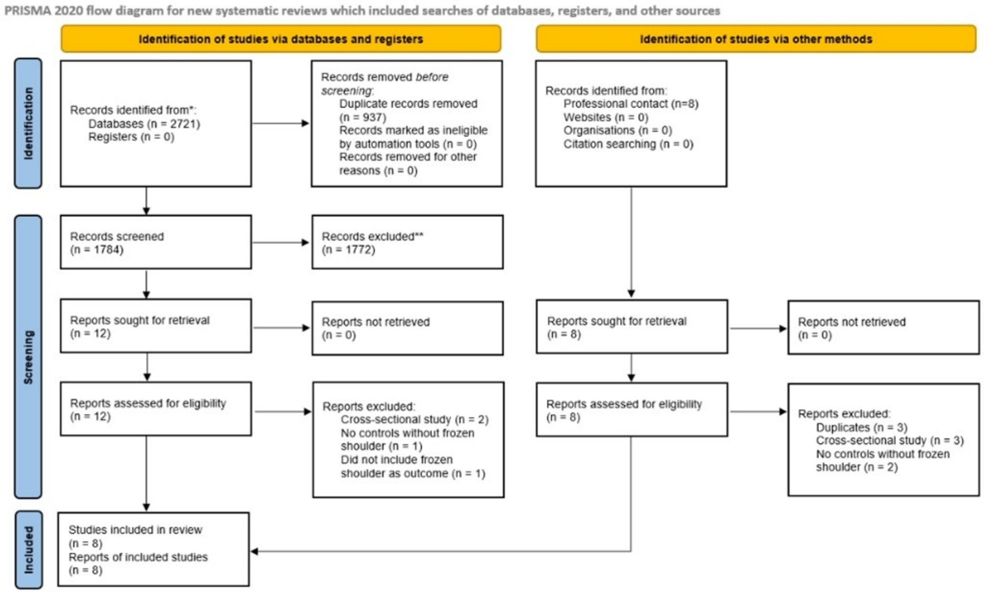

PRISMA flow diagram summarising record identification and study selection.

175x104mm (150 x 150 DPI)

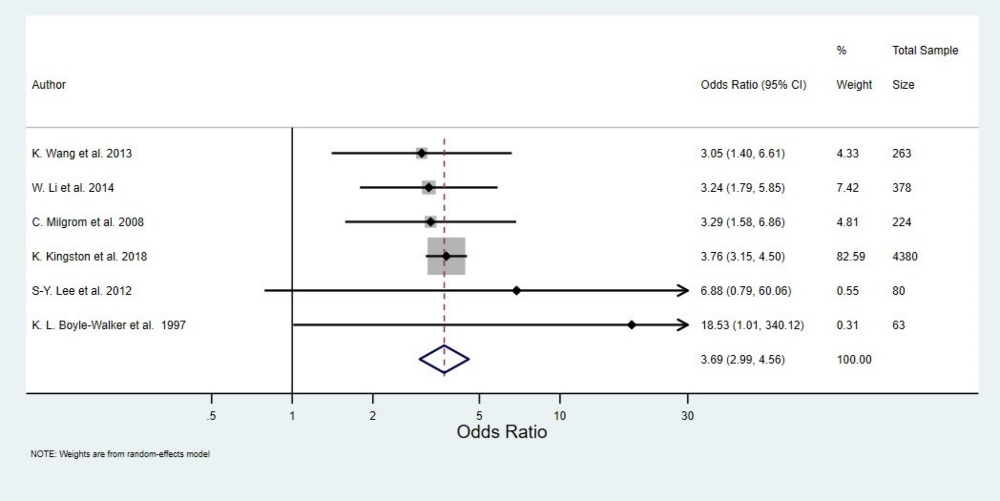

Random effects meta-analysis of the association between diabetes and the odds of developing frozen shoulder.

277x138mm (96 x 96 DPI)

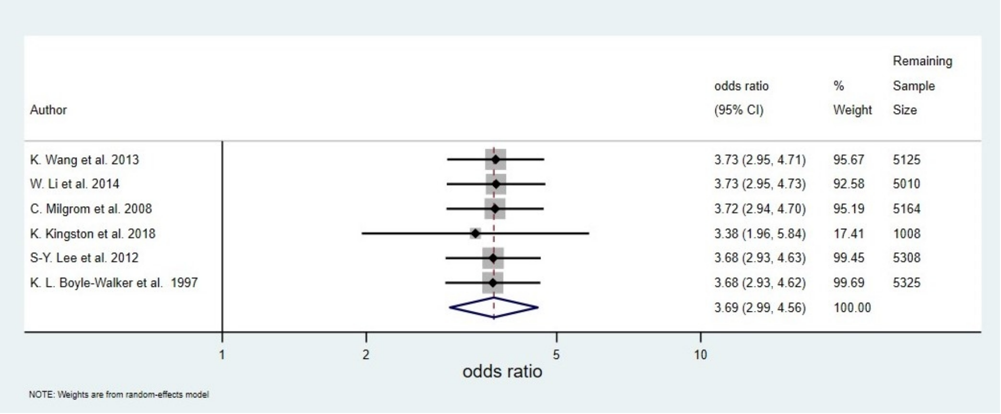

Influence plot showing the result of repeating the original meta-analysis (Figure 2), each time with a different primary study removed.

275x113mm (96 x 96 DPI)

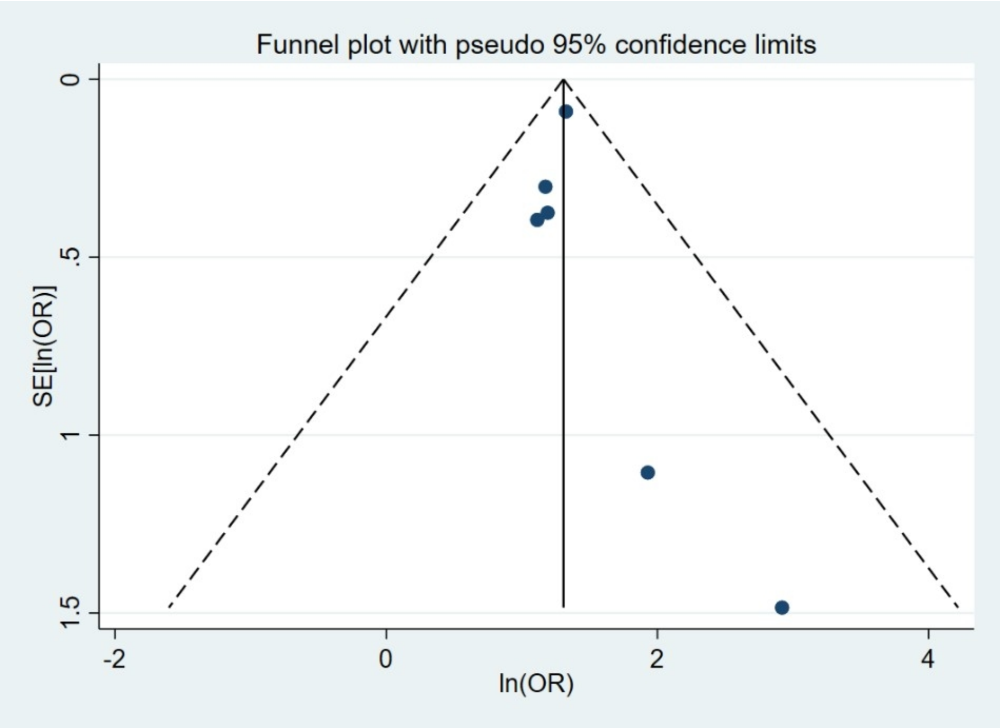

Funnel plot of log odds ratios for developing frozen shoulder in people with diabetes vs. those without diabetes.

109x79mm (220 x 220 DPI)

Appendix A

The following searches were originally conducted in December 2018 and updated in June 2021.

MEDLINE

Interface: OVID.

1. ((shoulder* or glenohumer* or subacromi* or acromi* or rotator cuff) adj3 (instability or bursitis or frozen or impinge* or tendonitis or tendinitis or pain* or osteoarthr* or periarthriti* or peri arthriti* or arthralgia)).ti,ab,kw.

2. Shoulder Impingement Syndrome/

3. exp Bursitis/

4. Rotator Cuff/

5. adhesive capsuliti*.ti,ab,kw.

6. Shoulder Pain/

7. or/1-6

8. exp Pain/

9. pain*.ti,ab,kw.

10. Arthralgia/

11. arthralgia.ti,ab,kw.

12. or/8-11

13. Shoulder/

14. Shoulder joint/

15. Acromioclavicular Joint/

16. (shoulder* or glenohumer* or subacromi* or acromi* or rotator cuff).ti,ab,kw.

17. or/13-16

18. 12 and 17

19. 7 or 18

20. exp Diabetes Mellitus/

21. diabet*.ti,ab,kw.

22. (DMi or DM i).ti,ab,kw.

23. (DM1 or DM 1).ti,ab,kw.

24. (DM2 or DM 2).ti,ab,kw.

25. (DMii or DM ii).ti,ab,kw.

26. (DM adj2 type).ti,ab,kw.

27. or/20-26

For peer review only

28. 19 and 27

29. exp animals/ not humans/

30. 28 not 29

EMBASE

Interface: OVID.

1. ((shoulder* or glenohumer* or subacromi* or acromi* or rotator cuff) adj3 (instability or bursitis or frozen or impinge* or tendonitis or tendinitis or pain* or osteoarthr* or periarthriti* or peri arthriti* or arthralgia)).ti,ab,kw.

2. exp shoulder impingement syndrome/

3. exp bursitis/

4. exp rotator cuff/

5. exp humeroscapular periarthritis/

6. adhesive capsuliti*.ti,ab,kw.

7. exp shoulder pain/

8. or/1-7

9. exp pain/

10. pain*.ti,ab,kw.

11. exp arthralgia/

12. arthralgia.ti,ab,kw.

13. or/9-12

14. exp shoulder/

15. Acromioclavicular Joint/

16. (shoulder* or glenohumer* or subacromi* or acromi* or rotator cuff).ti,ab,kw.

17. or/14-16

18. 13 and 17

19. 8 or 18

20. exp Diabetes Mellitus/

21. diabet*.ti,ab,kw.

22. (DMi or DM i).ti,ab,kw.

23. (DM1 or DM1).ti,ab,kw.

24. (DM2 or DM 2).ti,ab,kw.

25. (DMii or DM ii).ti,ab,kw.

For peer review only

26. (DM adj2 type).ti,ab,kw.

27. or/20-26

28. 19 and 27

29. exp animals/ not humans/

30. 28 not 29

31. limit 30 to embase

AMED

Interface: OVID.

1. ((shoulder* or glenohumer* or subacromi* or acromi* or rotator cuff) adj3 (instability or bursitis or frozen or impinge* or tendonitis or tendinitis or pain* or osteoarthr* or periarthriti* or peri arthriti* or arthralgia)).ti,ab.

2. exp Shoulder impingement syndrome/

3. exp Bursitis/

4. exp Rotator cuff/

5. adhesive capsuliti*.ti,ab.

6. exp shoulder pain/

7. or/1-6

8. exp Pain/

9. pain*.ti,ab.

10. exp Arthralgia/

11. arthralgia.ti,ab.

12. or/8-11

13. shoulder/

14. (shoulder* or glenohumer* or subacromi* or acromi* or rotator cuff).ti,ab.

15. or/13-14

16. 12 and 15

17. 7 or 16

18. exp Diabetes mellitus/

19. diabet*.ti,ab.

20. (DMi or DM i).ti,ab.

21. (DM1 or DM 1).ti,ab.

22. (DM2 or DM 2).ti,ab.

For peer review only

23. (DMii or DM ii).ti,ab.

24. (DM adj2 type).ti,ab.

25. or/18-24

26. 17 and 25

27. exp animals/ not humans/

28. 26 not 27

PsycINFO

Interface: OVID.

1. ((shoulder* or glenohumer* or subacromi* or acromi* or rotator cuff) adj3 (instability or bursitis or frozen or impinge* or tendonitis or tendinitis or pain* or osteoarthr* or periarthriti* or peri arthriti* or arthralgia)).ti,ab.

2. Shoulder Impingement Syndrome.ti,ab.

3. bursitis.ti,ab.

4. rotator cuff.ti,ab.

5. adhesive capsuliti*.ti,ab.

6. shoulder pain.ti,ab.

7. or/1-6

8. exp PAIN/

9. pain*.ti,ab.

10. arthralgia.ti,ab.

11. or/8-10

12. *"shoulder (anatomy)"/

13. shoulder*.ti,ab.

14. shoulder joint.ti,ab.

15. acromi*.ti,ab.

16. glenohumer*.ti,ab.

17. subacromi*.ti,ab.

18. or/12-17

19. 11 and 18

20. 7 or 19

21. exp DIABETES MELLITUS/

22. diabet*.ti,ab.

For peer review only

23. (DMi or DM i).ti,ab.

24. (DM1 or DM 1).ti,ab.

25. (DM2 or DM 2).ti,ab.

26. (DMii or DM ii).ti,ab.

27. (DM adj2 type).ti,ab.

28. or/21-27

29. 20 and 28

Web of Science

Science Citation Index Expanded and the Science Conference Proceedings Citation Index.

((

TS=(Shoulder* NEAR/3 instability) OR TS=(Shoulder* NEAR/3 bursitis) OR TS=(Shoulder* NEAR/3 frozen) OR TS=(Shoulder* NEAR/3 impinge*) OR TS=(Shoulder* NEAR/3 tendonitis) OR TS=(Shoulder* NEAR/3 tendinitis) OR TS=(Shoulder* NEAR/3 pain) OR TS=(Shoulder* NEAR/3 osteoarthr*) OR TS=(Shoulder* NEAR/3 periarthriti*) OR TS=(Shoulder* NEAR/3 "peri arthriti*") OR TS=(Shoulder* NEAR/3 arthralgia)

OR

TS=(glenohumer* NEAR/3 instability) OR TS=(glenohumer* NEAR/3 bursitis) OR TS=(glenohumer* NEAR/3 frozen) OR TS=(glenohumer* NEAR/3 impinge*) OR TS=(glenohumer* NEAR/3 tendonitis) OR TS=(glenohumer* NEAR/3 tendinitis) OR TS=(glenohumer* NEAR/3 pain) OR TS=(glenohumer* NEAR/3 osteoarthr*) OR TS=(glenohumer* NEAR/3 periarthriti*) OR TS=(glenohumer* NEAR/3 "peri arthriti*") OR TS=(glenohumer* NEAR/3 arthralgia)

OR

TS=(subacromi* NEAR/3 instability) OR TS=(subacromi* NEAR/3 bursitis) OR TS=(subacromi* NEAR/3 frozen) OR TS=(subacromi* NEAR/3 impinge*) OR TS=(subacromi* NEAR/3 tendonitis) OR TS=(subacromi* NEAR/3 tendinitis) OR TS=(subacromi* NEAR/3 pain) OR TS=(subacromi* NEAR/3 osteoarthr*) OR TS=(subacromi* NEAR/3 periarthriti*) OR TS=(subacromi* NEAR/3 "peri arthriti*") OR TS=(subacromi* NEAR/3 arthralgia)

OR

TS=(acromi* NEAR/3 instability) OR TS=(acromi* NEAR/3 bursitis) OR TS=(acromi* NEAR/3 frozen) OR TS=(acromi* NEAR/3 impinge*) OR TS=(acromi* NEAR/3 tendonitis) OR TS=(acromi* NEAR/3 tendinitis) OR TS=(acromi* NEAR/3 pain) OR TS=(acromi* NEAR/3 osteoarthr*) OR TS=(acromi* NEAR/3 periarthriti*) OR TS=(acromi* NEAR/3 "peri arthriti*") OR TS=(acromi* NEAR/3 arthralgia)

For peer review only

OR

TS=(“rotator cuff” NEAR/3 instability) OR TS=(“rotator cuff” NEAR/3 bursitis) OR TS=(“rotator cuff” NEAR/3 frozen) OR TS=(“rotator cuff” NEAR/3 impinge*) OR TS=(“rotator cuff” NEAR/3

tendonitis) OR TS=(“rotator cuff” NEAR/3 tendinitis) OR TS=(“rotator cuff” NEAR/3 pain)

OR TS=(“rotator cuff” NEAR/3 osteoarthr*) OR TS=(“rotator cuff” NEAR/3 periarthriti*) OR

TS=(“rotator cuff” NEAR/3 “peri arthriti*”) OR TS=(“rotator cuff” NEAR/3 arthralgia)

OR

TS=(“Rotator cuff”)

OR

TS=(“Adhesive capsuliti*”)

)

OR

TS=(arthralgia NEAR/3 shoulder* or arthralgia NEAR/3 glenohumer* or arthralgia NEAR/3

subacromi* or arthralgia NEAR/3 acromi* or arthralgia NEAR/3 “rotator cuff”)

OR TS=(pain* NEAR/3 shoulder* or pain* NEAR/3 glenohumer* or pain* NEAR/3 subacromi* or pain* NEAR/3 acromi* or pain* NEAR/3 “rotator cuff”)

)

And

TS=(diabet* or DM1 or “DM 1” or DM2 or “DM 2” or DMi or “DM i” or DMii or “DM ii” or

DM NEAR/2 type)

CINAHL

Interface: EBSCO. Filters: title or abstract

(

((shoulder* or glenohumer* or subacromi* or acromi* or “rotator cuff”) N3 (instability or bursitis or frozen or impinge* or tendonitis or tendinitis or pain* or osteoarthr* or periarthriti* or

“peri arthriti*” or arthralgia))

OR

(MH “Shoulder Impingement Syndrome”) OR (MH “Bursitis+”) OR (MH “Rotator Cuff+”) OR

(MH “Periarthritis”) OR (MH “Adhesive Capsulitis+”) OR (MH “Shoulder Pain”)

OR

((MH “Pain+”) or pain or (MH “Arthralgia+”) or arthralgia) and ((MH “Shoulder”) or (MH

“Acromioclavicular Joint”) or shoulder* or glenohumer* or subacromi* or acromi* or “rotator

cuff”)

For peer review only

)

AND

((MH “Diabetes Mellitus+”) or diabet* or (DMi or “DM i”) or (DM1 or “DM 1”) or (DMii or “DM ii”) or (DM2 or “DM 2”) or (DM N2 type))

Epistemonikos

Filters: title or abstract. Primary study. Not an RCT.

((“frozen shoulder” or “shoulder impinge*” or “shoulder bursitis” or “shoulder tendonitis” or “shoulder tendinitis” or “shoulder pain” or “pain in the shoulder” or “painful shoulder” or “shoulder osteoarthr*” or “shoulder joint arthr*” or “shoulder arthr”)

OR

(“glenohumeral impinge*” or “glenohumeral bursitis” or “glenohumeral tendonitis” or “glenohumeral tendinitis” or “glenohumeral pain” or “pain in the glenohumeral” or “glenohumeral osteoarthr*” or “glenohumeral arthr*” or “glenohumeral arthr”)

OR

(“subacromial impinge*” or “subacromial bursitis” or “subacromial tendonitis” or “subacromial tendinitis” or “subacromial pain” or “pain in the subacromial” or “subacromial osteoarthr*” or “subacromial arthr*” or “subacromial arthr”)

OR

“Rotator cuff”

OR

“periarthriti*”

OR

“peri arthriti*”

OR

“Adhesive capsuliti*”

)

AND

(diabet* or DM1 or DM2 or DMi or DMii or “type 1 DM” or “type 2 DM” or “type i DM” or “type ii DM”)

TRIP

For peer review only

("frozen shoulder" or "shoulder pain" or "periathriti*" or "peri arthriti*" or "adhesive capsuliti*" or "shoulder impingement" or "bursitis" or "rotator cuff") and "diabet*"

PEDro

Filters: body part = upper arm, shoulder or shoulder girdle

Title and abstract search: diabet*

Open Grey

Search 1: Diabet* and shoulder*

Search 2: Diabet* and glenohumer*

Search 3: Diabet* and subacromi*

Search 4: Diabet* and acromi*

Search 5: Diabet* and "rotator cuff*"

Search 6: Diabet* and bursitis

Search 7: Diabet* and periarthriti*

Search 8: Diabet* and "peri arthriti*"

Search 9: Diabet* and "adhesive capsuliti*"

Search 10: Diabet* and arthralgia

Grey literature report

Diabet*

For peer review only

Appendix B

Fig. B.1 Bar graph of QUIPS scores for each of the six bias domains: study participation, study attrition, diabetes/risk factor (RF) measurement, frozen shoulder/outcome measurement, study confounding, statistical analysis and reporting.

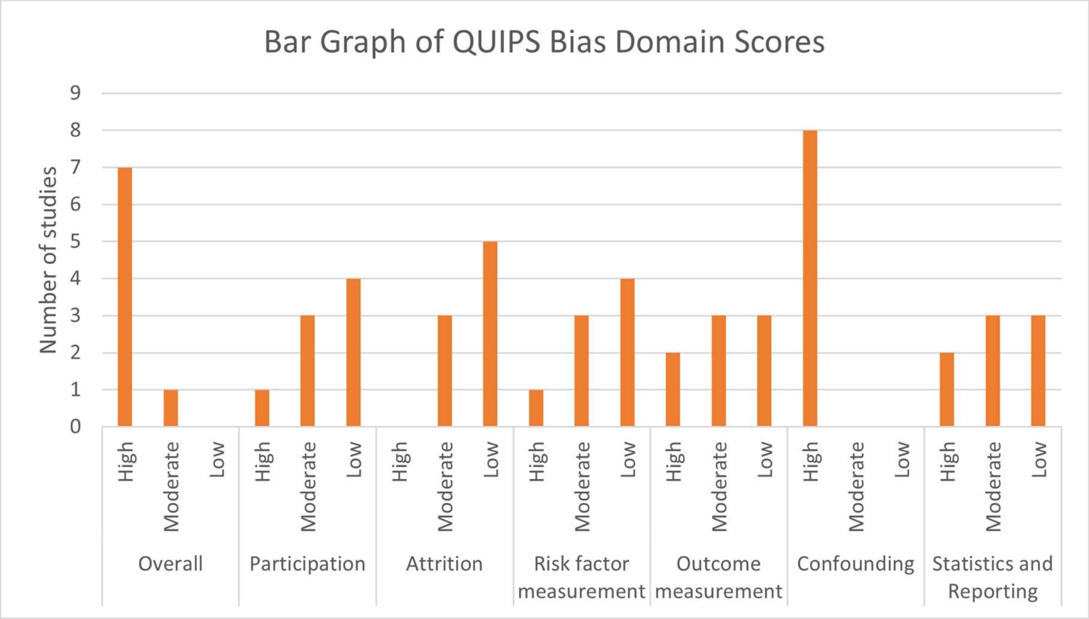

Appendix C

**Table C.1** Raw data from each study.				
**Case-Control Studies**				
Source	Number of cases	Number of controls	Number of cases with diabetes	Number of controls with diabetes
K. L. Boyle-Walker, et al., 1997 [32]	32	31	7	0
W. Li, et al., 2014 [33]	182	196	44	18
S-Y. Lee, et al., 2012 [34]	40	40	6	1
C. Milgrom, et al., 2008 [35]	126	98	37	11
K. Wang, et al., 2013 [36]	87	176	17	13
K. Kingston, et al., 2018 [37]	2190	2190	572	188
**Cohort studies**				
Source	Number of people with diabetes	Number of controls	Number of people with diabetes that developed frozen shoulder	Number of people without diabetes that developed frozen shoulder
Y-P. Huang, et al., 2013 [38]	78,827	236,481	946	2254
S-F. Lo, et al., 2013 [39]	5109	20,473	553	768

For peer review only

PRISMA 2020 for Abstracts Checklist

Section and Topic	Item #	Checklist item	Reported (Yes/No)
**TITLE**			
Title	1	Identify the report as a systematic review.	Y
**BACKGROUND**			
Objectives	2	Provide an explicit statement of the main objective(s) or question(s) the review addresses.	Y
**METHODS**			
Eligibility criteria	3	Specify the inclusion and exclusion criteria for the review.	Y
Information sources	4	Specify the information sources (e.g. databases, registers) used to identify studies and the date when each was last searched.	Y
Risk of bias	5	Specify the methods used to assess risk of bias in the included studies.	Y
Synthesis of results	6	Specify the methods used to present and synthesise results.	Y
**RESULTS**			
Included studies	7	Give the total number of included studies and participants and summarise relevant characteristics of studies.	Y
Synthesis of results	8	Present results for main outcomes, preferably indicating the number of included studies and participants for each. If meta-analysis was done, report the summary estimate and confidence/credible interval. If comparing groups, indicate the direction of the effect (i.e. which group is favoured).	Y
**DISCUSSION**			
Limitations of evidence	9	Provide a brief summary of the limitations of the evidence included in the review (e.g. study risk of bias, inconsistency and imprecision).	Y
Interpretation	10	Provide a general interpretation of the results and important implications.	Y
**OTHER**			
Funding	11	Specify the primary source of funding for the review.	Y
Registration	12	Provide the register name and registration number.	Y

From: Page MJ, McKenzie JE, Bossuyt PM, Boutron I, Hoffmann TC, Mulrow CD, et al. The PRISMA 2020 statement: an updated guideline for reporting systematic reviews. BMJ 2021;372:n71. doi: 10.1136/bmj.n71

For more information, visit: http://www.prisma-statement.org/

For peer review only - http://bmjopen.bmj.com/site/about/guidelines.xhtml

PRISMA 2020 Checklist

Section and Topic	Item #	Checklist item	Location where item is reported
**TITLE**			
Title	1	Identify the report as a systematic review.	Title
**ABSTRACT**			
Abstract	2	See the PRISMA 2020 for Abstracts checklist.	Abstract checklist attached
**INTRODUCTION**			
Rationale	3	Describe the rationale for the review in the context of existing knowledge.	Introduction paragraph 4
Objectives	4	Provide an explicit statement of the objective(s) or question(s) the review addresses.	Introduction paragraph 4
**METHODS**			
Eligibility criteria	5	Specify the inclusion and exclusion criteria for the review and how studies were grouped for the syntheses.	Section 2.3
Information sources	6	Specify all databases, registers, websites, organisations, reference lists and other sources searched or consulted to identify studies. Specify the date when each source was last searched or consulted.	Section 2.1
Search strategy	7	Present the full search strategies for all databases, registers and websites, including any filters and limits used.	Appendix A
Selection process	8	Specify the methods used to decide whether a study met the inclusion criteria of the review, including how many reviewers screened each record and each report retrieved, whether they worked independently, and if applicable, details of automation tools used in the process.	Section 2.2
Data collection process	9	Specify the methods used to collect data from reports, including how many reviewers collected data from each report, whether they worked independently, any processes for obtaining or confirming data from study investigators, and if applicable, details of automation tools used in the process.	Section 2.4
Data items	10a	List and define all outcomes for which data were sought. Specify whether all results that were compatible with each outcome domain in each study were sought (e.g. for all measures, time points, analyses), and if not, the methods used to decide which results to collect.	Section 2.3, 2.4
	10b	List and define all other variables for which data were sought (e.g. participant and intervention characteristics, funding sources). Describe any assumptions made about any missing or unclear information.	Section 2.4
Study risk of bias assessment	11	Specify the methods used to assess risk of bias in the included studies, including details of the tool(s) used, how many reviewers assessed each study and whether they worked independently, and if applicable, details of automation tools used in the process.	Section 2.4
Effect measures	12	Specify for each outcome the effect measure(s) (e.g. risk ratio, mean difference) used in the synthesis or presentation of results.	Section 2.5
Synthesis methods	13a	Describe the processes used to decide which studies were eligible for each synthesis (e.g. tabulating the study intervention characteristics and comparing against the planned groups for each synthesis (item #5)).	Section 2.5 lines 1-3.
	13b	Describe any methods required to prepare the data for presentation or synthesis, such as handling of missing summary statistics, or data conversions.	Section 2.5 lines 4-6
	13c	Describe any methods used to tabulate or visually display results of individual studies and syntheses.	Section 2.5 line 12
	13d	Describe any methods used to synthesize results and provide a rationale for the choice(s). If meta-analysis was performed, describe the model(s), method(s) to identify the presence and extent of statistical heterogeneity, and software package(s) used.	Section 2.5
	13e	Describe any methods used to explore possible causes of heterogeneity among study results (e.g. subgroup analysis, meta-regression).	n/a Not enough

For peer review only

PRISMA 2020 Checklist

Section and Topic	Item #	Checklist item	Location where item is reported
			studies present to do this
	13f	Describe any sensitivity analyses conducted to assess robustness of the synthesized results.	Section 2.5 lines 14-16
Reporting bias assessment	14	Describe any methods used to assess risk of bias due to missing results in a synthesis (arising from reporting biases).	n/a no missing results
Certainty assessment	15	Describe any methods used to assess certainty (or confidence) in the body of evidence for an outcome.	n/a
**RESULTS**			
Study selection	16a	Describe the results of the search and selection process, from the number of records identified in the search to the number of studies included in the review, ideally using a flow diagram.	Section 1 paragraph 1, Figure 1
	16b	Cite studies that might appear to meet the inclusion criteria, but which were excluded, and explain why they were excluded.	Figure 1
Study characteristics	17	Cite each included study and present its characteristics.	Table 1
Risk of bias in studies	18	Present assessments of risk of bias for each included study.	Table 2
Results of individual studies	19	For all outcomes, present, for each study: (a) summary statistics for each group (where appropriate) and (b) an effect estimate and its precision (e.g. confidence/credible interval), ideally using structured tables or plots.	Figure 2
Results of syntheses	20a	For each synthesis, briefly summarise the characteristics and risk of bias among contributing studies.	Table 1
	20b	Present results of all statistical syntheses conducted. If meta-analysis was done, present for each the summary estimate and its precision (e.g. confidence/credible interval) and measures of statistical heterogeneity. If comparing groups, describe the direction of the effect.	Section 3 paragraph 4
	20c	Present results of all investigations of possible causes of heterogeneity among study results.	n/a not enough studies to investigate causes of heterogeneity
	20d	Present results of all sensitivity analyses conducted to assess the robustness of the synthesized results.	Section 3 paragraph 4, Figure 3
Reporting biases	21	Present assessments of risk of bias due to missing results (arising from reporting biases) for each synthesis assessed.	n/a no missing results
Certainty of evidence	22	Present assessments of certainty (or confidence) in the body of evidence for each outcome assessed.	n/a
**DISCUSSION**			

For peer review only

PRISMA 2020 Checklist

Section and Topic	Item #	Checklist item	Location where item is reported
Discussion	23a	Provide a general interpretation of the results in the context of other evidence.	Section 4, paragraphs 1-3
	23b	Discuss any limitations of the evidence included in the review.	Section 4, paragraphs 4-6
	23c	Discuss any limitations of the review processes used.	n/a we did not come across any limitations of the review process
	23d	Discuss implications of the results for practice, policy, and future research.	Section 4, paragraphs 7-9, Section 5.
**OTHER INFORMATION**			
Registration and protocol	24a	Provide registration information for the review, including register name and registration number, or state that the review was not registered.	PROSPERO registration number is included in abstract
	24b	Indicate where the review protocol can be accessed, or state that a protocol was not prepared.	PROSPERO registration number is included in abstract
	24c	Describe and explain any amendments to information provided at registration or in the protocol.	PROSPERO registration number is included in abstract
Support	25	Describe sources of financial or non-financial support for the review, and the role of the funders or sponsors in the review.	Funding section
Competing interests	26	Declare any competing interests of review authors.	Conflicts of interest statement
Availability of data, code and other materials	27	Report which of the following are publicly available and where they can be found: template data collection forms; data extracted from included studies; data used for all analyses; analytic code; any other materials used in the review.	Appendix B, C

For peer review only

PRISMA 2020 Checklist

From: Page MJ, McKenzie JE, Bossuyt PM, Boutron I, Hoffmann TC, Mulrow CD, et al. The PRISMA 2020 statement: an updated guideline for reporting systematic reviews. BMJ 2021;372:n71. doi: 10.1136/bmj.n71
For more information, visit: http://www.prisma-statement.org/

For peer review only

BMJ Open

Diabetes as a Risk Factor for the Onset of Frozen Shoulder: A Systematic Review and Meta-Analysis

Journal:	*BMJ Open*
Manuscript ID	bmjopen-2022-062377.R2
Article Type:	Original research
Date Submitted by the Author:	24-Nov-2022
Complete List of Authors:	Dyer, Brett; Keele University, Primary Care Centre Versus Arthritis, School of Medicine<br>Rathod, Trishna; Keele University, Primary Care Centre Versus Arthritis, School of Medicine<br>Burton, Claire; Keele University, Primary Care Centre Versus Arthritis, School of Medicine<br>van der Windt, Danielle; Keele University, Primary Care Centre Versus Arthritis, School of Medicine<br>Bucknall, Milica; Keele University, Primary Care Centre Versus Arthritis, School of Medicine
**Primary Subject Heading**:	Rheumatology
Secondary Subject Heading:	Diabetes and endocrinology, Epidemiology
Keywords:	DIABETES & ENDOCRINOLOGY, EPIDEMIOLOGY, PRIMARY CARE, RHEUMATOLOGY

SCHOLARONE™
Manuscripts

I, the Submitting Author has the right to grant and does grant on behalf of all authors of the Work (as defined in the below author licence), an exclusive licence and/or a non-exclusive licence for contributions from authors who are: i) UK Crown employees; ii) where BMJ has agreed a CC-BY licence shall apply, and/or iii) in accordance with the terms applicable for US Federal Government officers or employees acting as part of their official duties; on a worldwide, perpetual, irrevocable, royalty-free basis to BMJ Publishing Group Ltd ("BMJ") its licensees and where the relevant Journal is co-owned by BMJ to the co-owners of the Journal, to publish the Work in this journal and any other BMJ products and to exploit all rights, as set out in our licence.

The Submitting Author accepts and understands that any supply made under these terms is made by BMJ to the Submitting Author unless you are acting as an employee on behalf of your employer or a postgraduate student of an affiliated institution which is paying any applicable article publishing charge ("APC") for Open Access articles. Where the Submitting Author wishes to make the Work available on an Open Access basis (and intends to pay the relevant APC), the terms of reuse of such Open Access shall be governed by a Creative Commons licence – details of these licences and which Creative Commons licence will apply to this Work are set out in our licence referred to above.

Other than as permitted in any relevant BMJ Author's Self Archiving Policies, I confirm this Work has not been accepted for publication elsewhere, is not being considered for publication elsewhere and does not duplicate material already published. I confirm all authors consent to publication of this Work and authorise the granting of this licence.

For peer review only

Title Page

Authors: Brett P Dyer, Trishna Rathod-Mistry, Claire Burton, Danielle A van der Windt, Miliça Blagojevic-Bucknall.

Title: Diabetes as a Risk Factor for the Onset of Frozen Shoulder: A Systematic Review and Meta-Analysis.

Affiliation: Primary Care Centre Versus Arthritis, School of Medicine, Keele University, Staffordshire, UK

Corresponding Author: Brett P Dyer. **Email:** b.p.dyer@keele.ac.uk

ORCID ID's: Brett P Dyer: 0000-0001-8039-7631, Trishna Rathod-Mistry: 0000-0002-6369-4746, Claire Burton 0000-0003-4688-3075, Danielle A van der Windt: 0000-0002-7248-6703, Miliça Blagojevic-Bucknall: 0000-0001-7230-7771.

Word count: 3275 words.

For peer review only

Title

Diabetes as a Risk Factor for the Onset of Frozen Shoulder: A Systematic Review and Meta-Analysis

Abstract

Objective Summarise longitudinal observational studies to determine whether diabetes (types 1 and 2) is a risk factor for frozen shoulder.

Design Systematic review and meta-analysis.

Data sources MEDLINE, EMBASE, AMED, PsycINFO, Web of Science core collection, CINAHL, Epistemonikos, Trip, PEDro, OpenGrey, and The Grey Literature Report were searched on January 2019 and updated in June 2021. Reference screening and emailing professional contacts were also utilised.

Eligibility criteria Longitudinal observational studies that estimated the association between diabetes and developing frozen shoulder.

Data extraction and synthesis Data extraction was completed by one reviewer and independently checked by another using a pre-defined extraction sheet. Risk of bias was judged using the Quality In Prognosis Studies tool. For studies providing sufficient data, random-effects meta-analysis was used to derive summary estimates of the association between diabetes and the onset of frozen shoulder.

Results A meta-analysis of six case-control studies including 5388 people estimated the odds of developing frozen shoulder for people with diabetes to be 3.69 (95% CI: 2.99, 4.56) times the odds for people without diabetes. Two cohort studies were identified, both suggesting diabetes was associated with frozen shoulder, with hazard ratios of 1.32 (95% CI: 1.22, 1.42) and 1.67 (95% CI: 1.46, 1.91). Risk of bias was judged as high in seven studies and moderate in one study.

Conclusions People with diabetes are more likely to develop frozen shoulder. Risk of unmeasured confounding was the main limitation of this systematic review. High-quality studies are needed to confirm the strength of, and understand reasons for, the association.

PROSPERO registration number CRD42019122963.

Funding This work was supported by Versus Arthritis grant number 21899.

Keywords: Diabetes, Frozen shoulder, Adhesive capsulitis, Risk factor, Meta-analysis

Strengths and limitations of this study

- This systematic review is the first to summarise the results of studies estimating the longitudinal association between diabetes and the onset of frozen shoulder.
- Robust meta-analytic methods were used to synthesise and analyse data.
- Sensitivity to influential estimates and sensitivity to small study bias were assessed.
- Risk of bias was judged to be high in seven studies and moderate in one study; this limits the certainty in evidence.
- Only two cohort studies were identified which meant that pooling of association estimates was not suitable.

For peer review only

1 - Introduction

Frozen shoulder, also known as adhesive capsulitis, is a painful and severely debilitating condition. The inflammatory contracture of the glenohumeral joint capsule in frozen shoulder restricts both active and passive range of motion, with loss of external rotation being especially characteristic of this condition [1].

Frozen shoulder generally presents between the ages of 50 and 60 years and rarely presents before 40 years [2]. Women (58%) are more likely to develop frozen shoulder than men (42%) [3]. The contralateral shoulder is also affected in 6% to 17% of patients [4]. Although the exact aetiology remains unclear, several factors have been found to be associated with frozen shoulder, including trauma [3], thyroid dysfunction [5-7], cardiovascular disease [2,8], metabolic factors [7,9-11], and other musculoskeletal conditions such as Dupuytren's contracture [12,13]. The most common comorbidity in people with frozen shoulder is diabetes [2], both type 1 and type 2 [6]. The prevalence of frozen shoulder in the general population is around 0.75% [1], but the prevalence of frozen shoulder in people with diabetes is much higher. A meta-analysis of 13 cross-sectional studies estimated the prevalence of frozen shoulder in populations with diabetes to be 13.4% (95% confidence interval (CI): 10.2%, 17.2%) [14].

Diabetes is a term used to describe a group of chronic diseases characterised by hyperglycaemia. The two most prevalent types of diabetes are type 1 and type 2, making up 8% and 90% of cases, respectively [15]. It is well-known that people with diabetes are at risk of complications such as cardiovascular disease, retinopathy, neuropathy, and nephropathy [16], although the musculoskeletal complications of diabetes are not as well-known [17]. Musculoskeletal conditions, such as frozen shoulder, can significantly affect the quality of a patient's life and should not be overlooked. Our previous systematic review and narrative synthesis of 28 studies has shown that patients with diabetes may experience worse outcomes from frozen shoulder than people without frozen shoulder [18].

It has been suggested that diabetes may be a cause of frozen shoulder through glycation processes and/or inflammatory processes leading to capsular fibrosis and subsequent contracture [7,19,20]. To understand whether diabetes could potentially be a cause of frozen shoulder it is necessary (although not sufficient) to have evidence of the temporal relationship between diabetes and frozen shoulder [21]. This systematic review aims to summarise evidence from longitudinal observational studies to understand the temporal relationship between diabetes and frozen shoulder.

2 - Methods

2.1 – Search Strategy

The protocol for this systematic review was registered on PROSPERO (CRD42019122963) and the review was conducted and reported using PRISMA guidelines [22]. A systematic literature search of MEDLINE, EMBASE, AMED, PsycINFO, Web of Science core collection, CINAHL, Epistemonikos, Trip, PEDro, OpenGrey, and The Grey Literature Report was carried out in January 2019 and updated in June 2021. Reference lists of eligible studies were screened. Additionally, a professional contact of one author (DAvdW) was contacted to identify further studies. We retrieved all epidemiological studies containing index terms (e.g. Medical Subject Headings) and free-text words related to diabetes and shoulder pain more generally (not limited to frozen shoulder) to reduce the risk of missing potentially relevant publications. The search strategy for MEDLINE, which was constructed with the support of a health information specialist, can be found in Appendix A.

2.2 – Study Selection

Reviewer BPD screened all titles and abstracts to check eligibility using the pre-defined inclusion and exclusion criteria, and reviewers MB-B and CB independently checked a 20% random sample. Reviewer BPD checked all full-texts for eligibility using the inclusion and exclusion criteria, and reviewers MB-B, CB, TR-M also

For peer review only

independently checked eligibility. Disagreements regarding the inclusion of studies were resolved through discussion with DAvdW.

2.3 – Inclusion Criteria

To be eligible for inclusion, studies were required to have a longitudinal, prospective or retrospective, observational study design. Cohort studies were required to have a study population consisting of people without frozen shoulder at inclusion and must have established whether diabetes was present at baseline (all types of diabetes were considered). Case-control studies were required to have a study population consisting of people with frozen shoulder and a control group without frozen shoulder, with diabetes defined as the exposure of interest. The paper must have presented an odds ratio, risk ratio or hazard ratio, or they must have presented sufficient data to allow the associations to be estimated. There were no restrictions to setting; population based as well as clinical cohorts were eligible. All non-English language papers were assessed by reviewers with appropriate language skills. Cross-sectional studies and case series were excluded. Studies were also excluded if a full text could not be obtained.

2.4 – Data Extraction and Risk of Bias

Data extraction was completed by reviewer BPD and was independently checked by reviewers MB-B and TR-M. Types of data extracted included details of study design, setting, sample characteristics, exposure/outcome/covariate measurement, inclusion and exclusion criteria, sample size, attrition, covariate conditioning, follow-up time, statistical analysis, association estimates (odds ratio, risk ratio or hazard ratio) or raw data to estimate association sizes if they were not already presented. Risk of bias was independently assessed by pairs of reviewers (BPD, MB-B, TR-M). Risk of bias was judged using the Quality In Prognosis Studies (QUIPS) tool [23]. The QUIPS tool covers six domains: (1) study participation, (2) study attrition, (3) prognostic/risk factor measurement, (4) outcome measurement, (5) study confounding, (6) statistical analysis and reporting. Each of the six domains is scored as being at a low, medium, or high risk of bias [23]. Domain scores were used to guide judgement of the overall risk of bias (scored as low, medium or high) for the study. Overall risk of bias was based on author judgement and the use of a tallied or summated score was avoided. All disagreements regarding data extraction and assessment of risk of bias were resolved by discussion.

2.5 – Data Analysis

Case-control studies and cohort studies were analysed separately. Narrative synthesis was used where less than five studies were present and a random-effects meta-analysis model was used to calculate a summary estimate when five or more studies were present. Cohort study associations were measured using hazard ratios and case-control study associations were estimated using odds ratios. Where adjusted and crude estimates were both presented, the adjusted estimate was used. Where a zero-cell count was present within the results of a study, a continuity correction of 0.5 was added to all cells for that study. Restricted maximum likelihood estimation [24] was used to estimate the between-study variance, τ^2, and the Hartung-Knapp-Sidik-Jonkman variance correction method [25] was used in the estimation of the pooled effect confidence interval. Heterogeneity was assessed using Cochran's Q statistic, complemented by the I^2 index [26]. Prediction intervals were not estimated since they are inaccurate when there is little heterogeneity ($I^2 < 0.3$), or an imbalance in study sizes exists, both of which were found in the meta-analysis in this review (see Section 3) [27]. A forest plot was used to visualise results of individual results and of the pooled estimate. Evidence of small-study bias was assessed with a funnel plot of log odds ratios against their standard errors [28]. A test for funnel plot asymmetry was not used since the meta-analysis included less than ten studies [29]. The influence of each study on the overall pooled estimate was assessed by repeating the meta-analysis, each time leaving out a single study [30]. Statistical analysis was carried out using Stata version 16.1 [31].

2.6 - Patient and Public Involvement

No patient involved.

For peer review only

3 - Results

The searches identified 1784 unique citations, 12 of which were selected for full-text screening, and eight studies consisting of a total of 346,278 people fulfilled the inclusion criteria (Figure 1). Table 1 summarises information on risk of bias, study design, setting, participants, sample size, and methods used for diagnosing diabetes and frozen shoulder. Of the eight studies that met the criteria for inclusion, six [32-37] had case-control designs and two [38,39] had cohort designs. Three studies [37-39] (including the two cohort studies)

Table 1. Characteristics of studies on diabetes as a risk factor for frozen shoulder

Source	Risk of Bias (QUIPS, overall assess-ment)	Design and Setting	% Female	Mean Age (years)	Sample Size	Method to diagnose diabetes and frozen shoulder	Variables conditioned on
**Case-control studies**							
K. L. Boyle-Walker, et al., 1997 [32]	High	Sex-Matched Case-Control at Physical Therapy Clinic in the USA	Case Group: 75%, Control Group: 68%	Not reported	Cases: 32, Controls: 31	Diabetes: Self-reported Questionnaire Frozen shoulder: Clinically diagnosed	Sex-matched
W. Li, et al., 2014 [33]	High	Hospital based case-control matched on time of hospitalisation in China	Case Group: 63%, Control Group: 55%	Cases: 57.2, Controls: 45.9	Cases: 182, Controls: 196	Diabetes: Face-to-face interview Frozen shoulder: Clinically diagnosed	Matched on time of hospitalisation, adjusted for history of minor shoulder trauma
S-Y. Lee, et al., 2012 [34]	High	Hospital based age- and sex-matched case-control in South Korea	Case Group: 55%, Control Group: not reported	Cases: 52.8, Controls: not reported	Cases: 40, Controls: 40	Diabetes: Unclear Frozen shoulder: Clinically diagnosed	Age- and sex-matched
C. Milgrom, et al., 2008 [35]	High	Hospital based age-matched case-control in Israel	Case Group: 60%, Control Group: 65%	Cases: 54.9, Controls: 55.4	Cases: 126, Controls: 98	Diabetes: If patient was receiving drug treatment for Diabetes or whose serum glucose was higher than 200 mg/dl Frozen shoulder: Clinically diagnosed	Age-matched
K. Wang, et al.,	High	Hospital based age- and sex-matched	Case Group:	Cases: 56, Controls:	Cases: 87, Controls:	Diabetes: Self-reported	Age- and sex-matched

collected information from electronic health records, four studies [33-36] were hospital-based, and one study [32] was based in a physical therapy clinic. Among the case-control studies, the percentage of female cases ranged from 52% to 75% and the mean age for cases ranged from 52.8 years to 57.2 years.

For peer review only

2013 [36]		case-control in Australia	64%, Control Group: 58%	55.3	176	Frozen shoulder: Clinically diagnosed	
K. Kingston, et al., 2018 [37]	High	Sex-matched case-control using electronic health records in the USA	Case Group: 58%, Control Group: 58%	Cases: 56.4, Controls: Not Reported	Cases: 2190, Controls: 2190	Diabetes: ICD-9 Code Frozen shoulder: ICD-9 Code	Sex-matched
**Cohort studies**							
Y-P. Huang, et al., 2013 [38]	High	Age- and sex-matched cohort with 3-year follow-up using electronic health records in Taiwan	Exposed Group: 47%, Non-Exposed Group: 47%	Exposed Group: 55.7, Non-Exposed Group: 55.5	Exposed Group: 78,827, Non-Exposed Group: 236,481	Diabetes: ICD-9 Code Frozen shoulder: ICD-9 Code	Age- and sex-matched. Multivariable analysis adjusted for age, sex, dyslipidaemia
S-F. Lo, et al., 2013 [39]	Moderate	Cohort with 8-year follow-up using electronic health records in Taiwan	Exposed Group: 52%, Non-Exposed Group: 51%	Not reported	Exposed Group: 5109, Non-Exposed Group: 20,473	Diabetes: ICD-9 Code Frozen shoulder: ICD-9 Code	Multivariable analysis adjusted for age, income, stroke, hypertension, hyperlipidaemia, obesity, chronic obstructive pulmonary disease

Presence of diabetes was identified using ICD-9 codes (codes to classify diseases, symptoms, clinical findings and causes of disease and injury) from electronic health records in three studies [37-39], self-reported in three studies [32,33,36], identified with a glucose test or if the patient was receiving drug treatment for diabetes in one study [35], and was unclear in one study [34]. Frozen shoulder was identified using [37-39] ICD-9 codes in three studies and was diagnosed clinically in five studies [32-36]. Only one study [39] reported the types of diabetes that the participants had. Lo et al. [39] stated that 296 (5.8%) of the 5109 people with diabetes in their study had type 1 diabetes. Two studies were conducted in Taiwan [38,39], two in the USA [32,37] and the remaining four were conducted in China [33], South Korea [34], Israel [35] and Australia [36].

Overall QUIPS risk of bias scores for each study can be found in Table 1 and full QUIPS assessments can be found in Table 2. Overall, there was a 75% agreement between reviewers across the individual bias domains, and reviewers agreed on 4 of the 8 overall risk of bias scores. One of the cohort studies [39] was scored as being at a moderate risk of bias for their overall study rating and the other seven studies were rated as being at a high risk of bias overall. A bar graph of the scores for individual risk of bias domains can be found in Appendix Figure B1. Risk of bias was generally high across most domains, but especially so for the risk of unaccounted confounding, which was scored as being at a high risk of bias in all eight studies. Five of the case-control studies [32,34-37] only accounted for age, gender or a combination of the two. One study [33] matched on the time of hospitalisation and adjusted for history of minor shoulder trauma. One cohort study [38] adjusted for age, sex and dyslipidaemia; the other cohort study [39] adjusted for age, income, stroke, hypertension, hyperlipidaemia, obesity and chronic obstructive pulmonary disease.

Table 2 QUIPS domain scores for each primary study

For peer review only

Source	Participation	Study Attrition	Risk Factor Measure-ment	Outcome Measure-ment	Confounding	Statistical Analysis and Presentation	Overall Risk of Bias
**Case-Control Studies**							
K. L. Boyle-Walker, et al., 1997 [32]	High	Moderate	High	Moderate	High	Moderate	High
W. Li, et al., 2014 [33]	Moderate	Low	Moderate	High	High	High	High
S-Y. Lee, et al., 2012 [34]	Moderate	Low	Moderate	Moderate	High	Moderate	High
C. Milgrom, et al., 2008 [35]	Moderate	Low	Low	Low	High	Low	High
K. Wang, et al., 2013 [36]	Low	Low	Low	Low	High	Low	High
K. Kingston, et al., 2018 [37]	Low	Moderate	Moderate	Low	High	Moderate	High
**Cohort studies**							
Y-P. Huang, et al., 2013 [38]	Low	Moderate	Low	High	High	High	High
S-F. Lo, et al., 2013 [39]	Low	Low	Low	Moderate	High	Low	Moderate

Six case-control studies including a total of 5388 people were pooled in a random-effects meta-analysis, with a pooled odds ratio of 3.69 (95% CI: 2.99, 4.56) (Figure 2). The raw data extracted from each study that was used to calculate odds ratios can be found in Appendix Table C1. The estimated between-study variance was small (τ^2<0.01, 95% CI: <0.01, 0.23) and little heterogeneity was detected (Q=2.07, df=5, p=0.84; I^2<0.01% (95% CI: <0.1%, 67.6%)), but the estimate for I^2 was imprecise as indicated by the wide 95% confidence interval. The influence analysis showed that excluding the largest study [37], which contained 4380 of the 5388 participants, greatly reduced the precision of the pooled estimate but did not substantially affect the value of the pooled estimate (Figure 3). Further, excluding any other single study did not substantially affect the value of the pooled estimate (Figure 3). The two studies with the smallest standard errors for their effect estimates had the largest odds ratio's, making the funnel plot appear unsymmetrical. However, due to the small number of studies contributing to the funnel plot, the asymmetrical appearance could be due to chance (Figure 4).

The two cohort studies that were identified used Cox proportional-hazards models and obtained results suggesting that people with diabetes were more at risk of developing frozen shoulder. One cohort study [38] using electronic health records from Taiwan, with a 3-year follow-up and consisting of 315,308 people reported an age-, sex- and dyslipidaemia-adjusted hazard ratio of 1.32 (95% CI: 1.22, 1.42). Another cohort study [39], with an 8-year follow-up, consisting of 25,582 people, also using electronic health records from Taiwan, estimated an age-, income-, stroke-, hypertension-, hyperlipidaemia-, obesity- and chronic obstructive pulmonary disease- adjusted hazard ratio of 1.67 (95% CI: 1.46, 1.91).

4 - Discussion

This systematic review aimed to summarise evidence from longitudinal observational studies to determine whether diabetes (types 1 and 2) is a risk factor for frozen shoulder.

Eight studies met the eligibility criteria for the review; each individual study demonstrated evidence to suggest that diabetes is associated with the onset of frozen shoulder. Our meta-analysis of six case-control studies yielded a pooled odds ratio of 3.69 (95% CI: 2.99, 4.56), and the value of the pooled estimate was robust to the omission of any individual study. The odds ratio estimates of all but one study [37] were imprecise with large

For peer review only

confidence intervals; this meant that the confidence intervals overlapped well, resulting in a small I^2 value. It is also important to note that Cochran's Q statistic should be interpreted with caution since the number of studies included in the analysis was small [40].

The funnel plot was unsymmetrical. However, given that a small number of studies were available, it was difficult to assess accurately whether any small-study bias was present or if the appearance was due to chance. Since our influence analysis has shown that the inclusion/exclusion of any individual study had very little impact on the pooled effect estimate, any potential small-study bias would be unlikely to substantially affect the results.

Two cohort studies were identified, both of which corroborate the evidence from the six case-control studies reported above, that people with diabetes are more likely to develop frozen shoulder than those without diabetes. Of the two cohort studies, one was deemed to be at a high risk of bias and the other at a moderate risk of bias. The hazard ratios in the two studies did differ, which could have partly been due to the differences in the covariates that were adjusted for and/or the differences in the duration of follow-up. Both studies were rated as being at a high of bias for the outcome-measurement domain as the length of follow-up (3 years [38] and 8 years [39]) was deemed too short to establish whether a patient would develop frozen shoulder in the future. Previous studies have suggested that the duration of diabetes may be associated with the risk of developing frozen shoulder [41,42], with one of the cohort studies in this review also stating that their study suggested that "the development of [frozen shoulder] is associated with the duration of diabetes" [38]. Therefore, future studies should ensure that the follow-up period is long enough to observe participants from diabetes diagnosis through to the ages for which frozen shoulder is common. A cross-sectional study of 1373 patients presenting with frozen shoulder estimated that the mean age of onset for frozen shoulder was 55.4 years with a standard deviation of 9.9 years [3].

The following three paragraphs describe some limitations that may complicate the understanding of the association between diabetes and the onset of frozen shoulder.

The two cohort studies in the review were both conducted using Electronic Health Records (EHRs). EHR datasets can provide large sample sizes with long follow-up periods and detailed patient medical record history [43]. Misdiagnosis and miscoding in EHRs are common limitations and could potentially result in a risk of bias for frozen shoulder measurement [44]. Research in the UK [45] and in the Netherlands [46] has shown that general practitioners often use non-specific shoulder pain codes instead of codes for specific shoulder conditions, e.g., frozen shoulder. This would lead to an underdiagnosis of frozen shoulder. Further, this misclassification may be differential since clinicians may feel more confident in providing a specific frozen shoulder diagnosis in patients with diabetes due to the pre-existing knowledge of the association between the two conditions. Conversely, it has also been noted that frozen shoulder is sometimes used as a "waste-bin diagnosis" for patients presenting with any stiff and painful shoulder [47]. Thus, EHR data may include other shoulder conditions with similar clinical presentations being coded as frozen shoulder.

Another important limitation was the overall poor adjustment for confounding variables. All eight studies were rated as being at a high risk of unaccounted confounding. In each study, confounders were either ignored [32,34-38] or inappropriate statistical methods, such as univariable prefiltering and stepwise selection, were used [33,38,39]. These methods are especially poorly suited for aetiologic models [48]. Thus, these studies may have missed potentially important confounders [33,38,39] or erroneously adjusted for mediators, such as stroke [39].

The systematic review is also limited by there being only two cohort studies, meaning that pooling association estimates was not possible. Cohort studies are particularly useful for gaining a better understanding of temporal associations, as this review aimed to do. Further, both cohort studies were conducted in Taiwan using existing data from EHRs. Future studies with prospective designs will help to gauge whether the findings of these two cohort studies are reproducible, and whether the results are consistent across different populations.

For peer review only

Previously, a meta-analysis of cross-sectional studies established that frozen shoulder was more prevalent in people with diabetes than among people without diabetes. This systematic review provides evidence of a temporal relationship between diabetes and frozen shoulder. Understanding the temporal relationship is key to explaining why diabetes and frozen shoulder are associated, however further high-quality research with appropriate methods and study design is required to confirm the strength of the association and establish whether diabetes is indeed a cause of frozen shoulder.

Whilst sound and reliable epidemiological evidence of a causal relationship between diabetes and frozen shoulder is currently unavailable, elsewhere in the literature, researchers have hypothesised about potential pathological mechanisms through which diabetes may lead to frozen shoulder. Current evidence, based on histological studies, suggests that a pathophysiological process consisting of chronic inflammation and capsular fibrosis leads to the contracture in frozen shoulder [49,50]. It has been hypothesised that the accumulation of advanced glycation end products (AGEs), which lead to the cross-linking of collagen [51,52], may explain the fibrosis in the capsule of frozen shoulder patients [33]. Glycation is a process by which simple sugars bond to proteins, which is enhanced by persistent hyperglycaemia. Thus, the role of glycation and AGEs in the fibrosis of the shoulder capsule could potentially be a reason why diabetes is associated with frozen shoulder. Another potential reason why diabetes may be associated with frozen shoulder is that hyperglycaemia may induce proinflammatory cytokines [53] which have been found to be elevated in the capsule and synovium of frozen shoulder patients [54].

The association between glycaemic control and the risk of developing frozen shoulder should also be a focus for future research. One study found evidence to suggest that poor long-term glycaemic control in people with diabetes is associated with an increased incidence of frozen shoulder [55], whilst another study found no association between HbA1c level in people with diabetes and the prevalence of frozen shoulder [56]. Further research is required to investigate whether glycaemic control is associated with the development of frozen shoulder.

5 - Conclusion

In summary, people with diabetes are more at risk of developing frozen shoulder than people without diabetes. However, existing research is limited by the high risk of unmeasured confounding. To better understand the nature of the relationship between diabetes and the onset of frozen shoulder, it is necessary to have high-quality cohort studies that use causal inference methods that are appropriate for aetiologic modelling. Given the existing evidence that has been summarised in this review, clinicians should consider checking whether patients with diabetes are experiencing shoulder pain at their routine follow-up appointments. An early diagnosis will help the clinician to provide treatment for the pain and lack of function that result from frozen shoulder.

6 - Acknowledgements

This study was supported by the Versus Arthritis PhD scholarship scheme [grant number 21899]. The author would also like to thank the information specialists of evidence synthesis team at the Primary Care Centre Versus Arthritis for their advice regarding the search strategy, and Elaine Willmore for taking time to check the results of our searches for this review. Further thanks are given to Dr Linda Chesterton, who was involved in the initial conceptualisation of this study. Claire Burton is funded by an NIHR (National Institute for Health Research) Clinical Lectureship. The views expressed are those of the authors and not necessarily those of the National Health Service, the NIHR, or the Department of Health and Social Care.

Abbreviations

QUIPS - Quality In Prognosis Studies

For peer review only

CI – Confidence interval

Q – Cochran's Q statistic

df – Degrees of freedom

Ethics Approval

No Ethics Committee or Institutional Board approval is required.

No human participants included.

Author contributions

All authors (BPD, CB, DvdW, MB-B and TR-M) contributed to the conception of the study and systematic review study selection. Data extraction and risk of bias assessment was performed by BPD, MB-B and TR-M. BPD performed the meta-analysis and narrative synthesis of the results and drafted the initial manuscript. All authors (BPD, CB, DvdW, MB-B and TR-M) contributed to editing and approval of the final manuscript.

Funding

This work was supported by Versus Arthritis grant number 21899. Claire Burton is funded by an NIHR (National Institute for Health Research) Clinical Lectureship. The views expressed are those of the authors and not necessarily those of the National Health Service, the NIHR, or the Department of Health and Social Care.

Data availability

Data have been included in Table 1 and Appendix Table C.1.

Conflicts of interest statement

The authors have no conflicts of interest.

Supplementary Material

Appendix A contains the search strategy for MEDLINE.

Appendix B contains full QUIPS risk of bias assessments for each primary study and a bar graph of QUIPS scores for each of the six bias domains.

Appendix C contains the raw data extracted from each primary study that was used to calculate the odds ratios included in the meta-analysis.

References

[1] Bunker T. Frozen Shoulder. *Orthopaedics and Trauma*. 2011; 25(1):11-8. https://doi.org/10.1016/j.mporth.2011.01.007

For peer review only

[2] Dias R, Cutts S, Massoud S. Frozen shoulder. *BMJ*. 2005; 331:1453-6. https://doi.org/10.1136/bmj.331.7530.1453

[3] Cho C-H, Koo TW, Cho N-S, Park K-J, Lee BG, Shin D, Choi S, Cho S-H, Kim M-S, Ko S-H, Kim C-H, Park J-Y, Yoo Y-S. Demographic and Clinical Characteristics of Primary Frozen Shoulder in a Korean Population. *Clin Shoulder Elbow*. 2015; 18(3):133-7. doi: 10.5397/cise.2015.18.3.133

[4] Rizk TE, Pinals RS. Frozen shoulder. *Seminars in Arthritis and Rheumatism.* 1982; 11(4):440-52. doi: 10.1016/0049-0172(82)90030-0

[5] Cohen C, Tortato T, Silva OBS, Ferreira Leal M, Ejnisman B, Faloppa F. Association between frozen shoulder and thyroid diseases: strengthening the evidences. *Rev Bras Ortop.* 2020; 55(4):483–9. doi: 10.1055/s-0039-3402476

[6] Huang S-W, Lin J-W, Wang W-T, Wu C-W, Lious T-H, Lin H-W. Hyperthyroidism is a risk factor for developing adhesive capsulitis of the shoulder: a nationwide longitudinal population-based study. *Sci Rep*. 2014; 25(4):4183. doi: 10.1038/srep04183

[7] Cucchi D, Marmotti A, De Giorgi S, Costa A, D'Apolito R, Conca M, Russo A, Saccomanno MF, de Girolamo L. Risk Factors for Shoulder Stiffness: Current Concept. *Joints*. 2017; 5(4):217-23. doi: 10.1055/s-0037-1608951

[8] Nagy MT, MacFarlane RJ, Khan Y, Waseem M. The frozen shoulder: myths and realities. *Open Orthop J*, 2013; 7:352-355. doi: 10.2174/1874325001307010352

[9] Sung CM, Sung TS, Park HB. Are serum lipids involved in primary frozen shoulder? A case-control study. *J Bone Joint Surg*. 2014; 96(21):1828–33. doi: 10.2106/JBJS.M.00936

[10] Nayak SP, Panda CK. Is hyperlipidaemia a cause of primary frozen shoulder? A case-controlled study. *J. Evid based med. healthc.* 2017; 4(13):697-710. doi 10.18410/jebmh/2017/135

[11] Austin D, Gans I, Park M, Carey J, Kelly J. The association of metabolic syndrome markers with adhesive capsulitis. *J Shoulder and Elbow Surg.* 2014; 23(7):1043-51. doi: 10.1016/j.jse.2013.11.004

[12] Smith SP, Devaraj VS, Bunker TD. The association between frozen shoulder and Dupuytren's disease. *J Shoulder Elbow Surg*. 2001; 10(2):149-51. doi: 10.1067/mse.2001.112883.

[13] Bunker TD, Anthony PP. The pathology of frozen shoulder. A Dupuytren-like disease. *J Bone Joint Surg Br.* 1995; 77(5):677-83. PMID: 7559688.

[14] Zreik NH, Malik RA, Charalambos CP. Adhesive capsulitis of the shoulder and diabetes: a meta-analysis of prevalence. *MLTJ.* 2016; 6(1):26-34. doi: 10.11138/mltj/2016.6.1.026

[15] NHS Digital. *National Diabetes Audit 2017/18 - Report 1 Care Processes and Treatment Targets.* 2020. Accessed: 8th September 2022 https://digital.nhs.uk/data-and-information/publications/statistical/national-diabetes-audit/report-1-care-processes-and-treatment-targets-2017-18-short-report

[16] Fowler MJ. Microvascular and Macrovascular Complications of Diabetes. *Clin Diabetes*. 2008; 26(2):77–82. https://doi.org/10.2337/diaclin.26.2.77

[17] Sözen T, Başaran NC, Tınazlı M, Özışık L. Musculoskeletal problems in diabetes mellitus. Eur J Rheumatol, 2018; 5(4):258–65. doi: 10.5152/eurjrheum.2018.18044

[18] Dyer BP, Burton C, Rathod-Mistry T, Blagojevic-Bucknall M, van der Windt DA. Diabetes as a prognostic factor in frozen shoulder: a systematic review. Arch Rehabil Res Clin Transl. 2021; 3:100141. https://doi.org/10.1016/j.arrct.2021.100141

[19] Hsu C-L, Sheu WH-H. Diabetes and shoulder disorders. *J Diabetes Investig*. 2016; 7(5):649-51. https://doi.org/10.1111/jdi.12491

For peer review only

[20] Pietrzak M, Adhesive capsulitis: An age-related symptom of metabolic syndrome and chronic low-grade inflammation? Med Hypotheses. 2016; 88:12-7. doi: 10.1016/j.mehy.2016.01.002.

[21] Rothman KJ, Greenland S, Lash TL. *Modern Epidemiology*: 3rd ed. Lippincott Williams & Wilkins. 2013.

[22] Moher D, Liberati A, Tetzlaff J, Altman DG. Preferred reporting items for systematic reviews and meta-analyses: the PRISMA statement. *BMJ.* 2009; 339:b2535. doi: 10.1136/bmj.b2535

[23] Hayden JA, van der Windt DA, Cartwright JL, Côté P, Bombardier C. Assessing bias in studies of prognostic factors. *Ann Intern Med*. 2019; 158(4):280–6. doi: 10.7326/0003-4819-158-4-201302190-00009

[24] Veroniki AA, Jackson D, Viechtbauer W, Bender R, Bowden J, Knapp G, Kuss O, Higgins JPT, Langan D, Salanti G. Methods to estimate the between-study variance and its uncertainty in meta-analysis. *Res Synth Meth. 2016;* 7(1):55-79. doi: 10.1002/jrsm.1164

[25] IntHout J, Ioannidis JPA, Borm GF. The Hartung-Knapp-Sidik-Jonkman method for random-effects meta-analysis is straightforward and considerably outperforms the standard Der-Simonian-Laird method. *BMC Med Res Methodol*. 2014; 14:25. https://doi.org/10.1186/1471-2288-14-25

[26] Higgins JPT, Thompson SG, Deeks JJ, Altman DG. Measuring inconsistency in meta-analyses. *BMJ.* 2003; 327:557-60. https://doi.org/10.1136/bmj.327.7414.557

[27] Partlett C, Riley RD. Random effects meta-analysis: Coverage performance of 95% confidence and prediction intervals following REML estimation. *Statist. Med*. 2016; 36(2):301-17. https://doi.org/10.1002/sim.7140

[28] Sterne JAC, Harbord RM. Funnel Plots in Meta-analysis. *Stata Journal*. 2004; 4(2):127-41. https://doi.org/10.1177/1536867X0400400204

[29] Higgins JPT, Green S. Cochrane Handbook for Systematic Reviews of Interventions Version 5.1.0 [updated March 2011]. *The Cochrane Collaboration*. 2011.

[30] Viechtbauer W, Cheung MWL. Outlier and influence diagnostics for meta-analysis. *Res Syn Meth. 2010;* 1(2):112-25. doi: 10.1002/jrsm.11

[31] StataCorp. 2019. *Stata Statistical Software: Release 16.* College Station, TX: StataCorp LLC.

[32] Boyle-Walker KL, Gabard DL, Bietsch E, Masek-VanArsdale DM, Robinson BL. A profile of patients with adhesive capsulitis. *J Hand Ther*. 1997; 10(3):222-8. https://doi.org/10.1016/S0894-1130(97)80025-7

[33] Li W, Lu N, Xu H, Wang H, Huang J. Case control study of risk factors for frozen shoulder in China. *Int J Rheum Dis*. 2014; 18(5):508-13. https://doi.org/10.1111/1756-185X.12246

[34] Lee S-Y, Park J, Song S-W. Correlation of MR Arthrographic Findings and Range of Shoulder Motions in Patients With Frozen Shoulder. *American Journal of Roentgenology*. 2012; 198(1):173-9. doi: 10.2214/AJR.10.6173

[35] Milgrom C, Novack V, Weil Y, Jaber S, Radeva-Petrova DR, Finestone A. Risk factors for idiopathic frozen shoulder. *Isr Med Assoc J*. 2008; 10(5):361-4. PMID: 18605360

[36] Wang K, Ho V, Hunter-Smith DJ, Beh PS, Smith KM, Weber AB. Risk factors in idiopathic adhesive capsulitis: a case control study. *J Shoulder Elbow Surg*. 2013; 22(7):24-9. doi: 10.11138/mltj/2016.6.1.026

[37] Kingston K, Curry EJ, Galvin JW, Li X. Shoulder adhesive capsulitis: epidemiology and predictors of surgery. *J Shoulder Elbow Surg*. 2018; 27(8):1437-43. doi: 10.1016/j.jse.2018.04.004

[38] Huang Y-P, Fann C-Y, Chiu Y-H, Yen M-F, Chen L-S, Chen H-H, Pan S-L. Association of Diabetes Mellitus With the Risk of Developing Adhesive Capsulitis of the Shoulder: A Longitudinal Population-Based Follow up Study. *Arthritis Care & Research*. 2013; 65(7):1197-202. https://doi.org/10.1002/acr.21938

For peer review only

[39] Lo S-F, Chu S-W, Muo C-H, Meng N-H, Chou L-W, Huang W-C, Huang C-M, Sung F-C. Diabetes mellitus and accompanying hyperlipidemia are independent risk factors for adhesive capsulitis: a nationwide population-based cohort study. *Rheumatology International*. 2014; 34:67-74. doi: 10.1007/s00296-013-2847-4

[40] Hardy RJ, Thompson SG. Detecting and describing heterogeneity in meta-analysis. *Stat Med*. 1998; 17(8):841-56. doi: 10.1002/(sici)1097-0258(19980430)17:8<841::aid-sim781>3.0.co;2-d

[41] Thomas SJ, McDougall C, Brown IDM, Jaberoo M-C, Stearns A, Ashraf R, Fisher M, Kelly IG. Prevalence of symptoms and signs of shoulder problems in people with diabetes mellitus. *J Shoulder and Elbow Surg.* 16(6); 748-51. doi: 10.1016/j.jse.2007.02.133

[42] Bridgeman JF, Periarthritis of the shoulder and diabetes mellitus. *Ann Rheum Dis.* 31: 69-71; 1972. doi: 10.1136/ard.31.1.69

[43] Herrett E, Gallagher AM, Bhaskaran K, Forbes H, Mathur R, van Staa T, Smeeth L. Data Resource Profile: Clinical Practice Research Datalink (CPRD). *Int J Epidemiol*. 2015; 44(3):827-36. doi: 10.1093/ije/dyv098.

[44] Young JC, Conover MM, Jonsson Funk M. Measurement error and misclassification in electronic medical records: methods to mitigate bias. *Curr Epidemiol Rep*. 2018; 5(4): 343–356. doi: 10.1007/s40471-018-0164-x

[45] Linsell L, Dawson J, Zondervan K, Rose P, Randall T, Fitzpatrick R, Carr A. Prevalence and incidence of adults consulting for shoulder conditions in UK primary care; patterns of diagnosis and referral. *Rheumat* 2006; 45(2):215-21.

[46] Dorrestijn O, Greving K. Patients with shoulder complaints in general practice: consumption of medical care. *Rheumatol*. 2011; 50(2): 389-95.

[47] Bunker T Time for a new name for frozen shoulder – contracture of the shoulder. *Shoulder Elbow.* 2009; 1(1):4-9. https://doi.org/10.1111/j.1758-5740.2009.00007.x

[48] Heinze G, Dunkler D. Five myths about variable selection. *Transplant International*. 2017; 30(1):6-10. https://doi.org/10.1111/tri.12895

[49] Wong PL, Tan HC. A Review on Frozen Shoulder. *Singapore Med J*. 2010; 51(9):694-7. PMID: 20938608

[50] Cho C-H, Song K-S, Kim B-S, Kim D.H, Lho Y-M. Biological Aspect of Pathophysiology for Frozen Shoulder. *BioMed Research International*. 2018; Article ID 7274517. https://doi.org/10.1155/2018/7274517

[51] Hwang KR, Murrell GAC, Millar NL, Bonar F, Lam R, Walton JR. Advanced glycation end products in idiopathic frozen shoulders. *J Shoulder and Elbow Surg.* 2016; 25(6): 981-8. doi: 10.1016/j.jse.2015.10.015

[52] Goldin A, Beckman JA, Schmidt AM, Creager MA. Advanced Glycation End Products. *Circulation*. 2006; 114(6): 597-605. doi: 10.1161/CIRCULATIONAHA.106.621854.

[53] Shanmugam N, Reddy MA, Guha M, Natarajan R. High glucose-induced expression of proinflammatory cytokine and chemokine genes in monocytic cells. *Diabetes*. 2003; 52(5):1256-64. doi: 10.2337/diabetes.52.5.1256

[54] Lho Y-M, Ha E, Cho C-H, Song K-S, Min B-W, Bae K-C, Lee K-J, Hwang I, Park H-B. Inflammatory cytokines are overexpressed in the subacromial bursa of frozen shoulder. *J Shoulder and Elbow Surg.* 2013; 22(5): 666-72. https://doi.org/10.1016/j.jse.2012.06.014

[55] Chan JH, Ho BS, Alvi HM, Saltzman MD, Marra G. The relationship between the incidence of adhesive capsulitis and hemoglobin A1c. *J Shoulder and Elbow Surg.* 2017; 26(10):1834-7. https://doi.org/10.1016/j.jse.2017.03.015

[56] Yian EH, Contreras R, Sodl JF. Effects of Glycemic Control on Prevalence of Diabetic Frozen Shoulder. *J Bone Joint Surg.* 2012; 94(10):919-23. doi: 10.2106/JBJS.J.01930

Figure Captions

Fig. 1 PRISMA flow diagram summarising record identification and study selection.

Fig. 2 Random effects meta-analysis of the association between diabetes and the odds of developing frozen shoulder.

Fig. 3 Influence plot showing the result of repeating the original meta-analysis (Figure 2), each time with a different primary study removed.

Fig. 4 Funnel plot of log odds ratios for developing frozen shoulder in people with diabetes vs. those without diabetes.

For peer review only

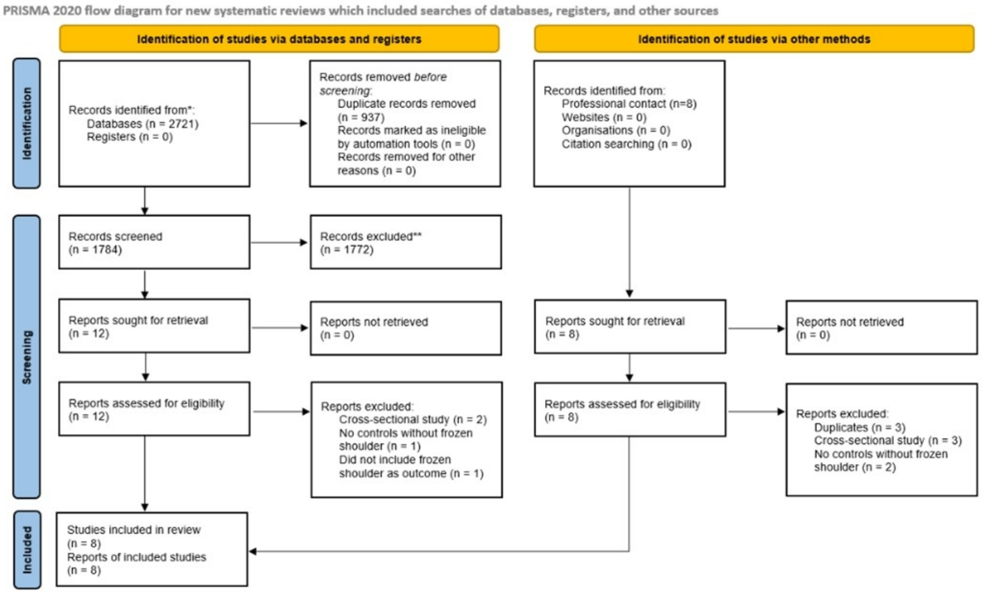

PRISMA flow diagram summarising record identification and study selection.

175x104mm (150 x 150 DPI)

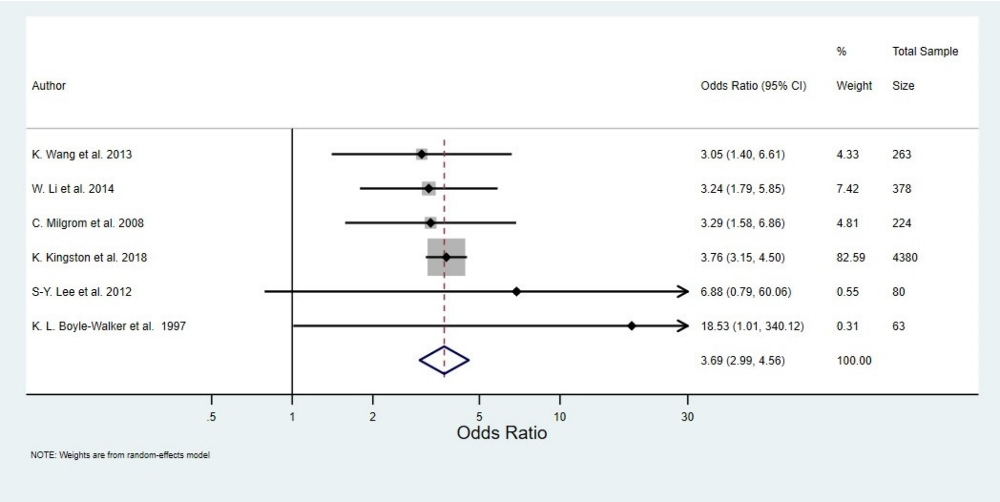

Random effects meta-analysis of the association between diabetes and the odds of developing frozen shoulder.

277x138mm (96 x 96 DPI)

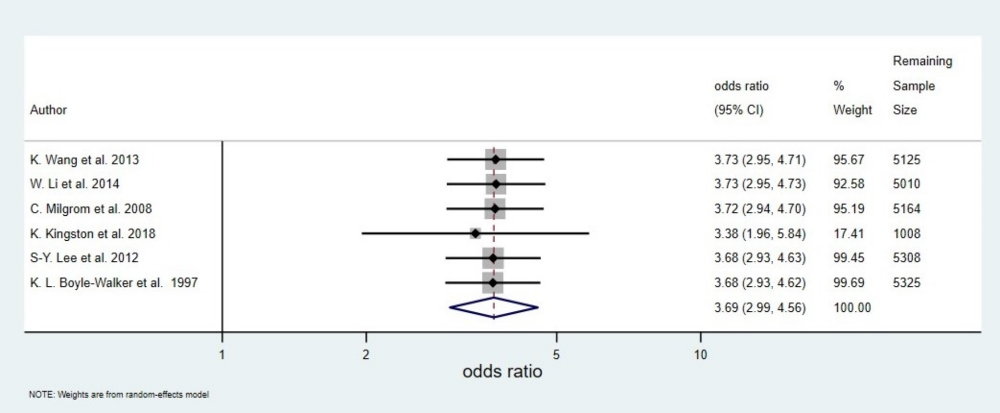

Influence plot showing the result of repeating the original meta-analysis (Figure 2), each time with a different primary study removed.

275x113mm (96 x 96 DPI)

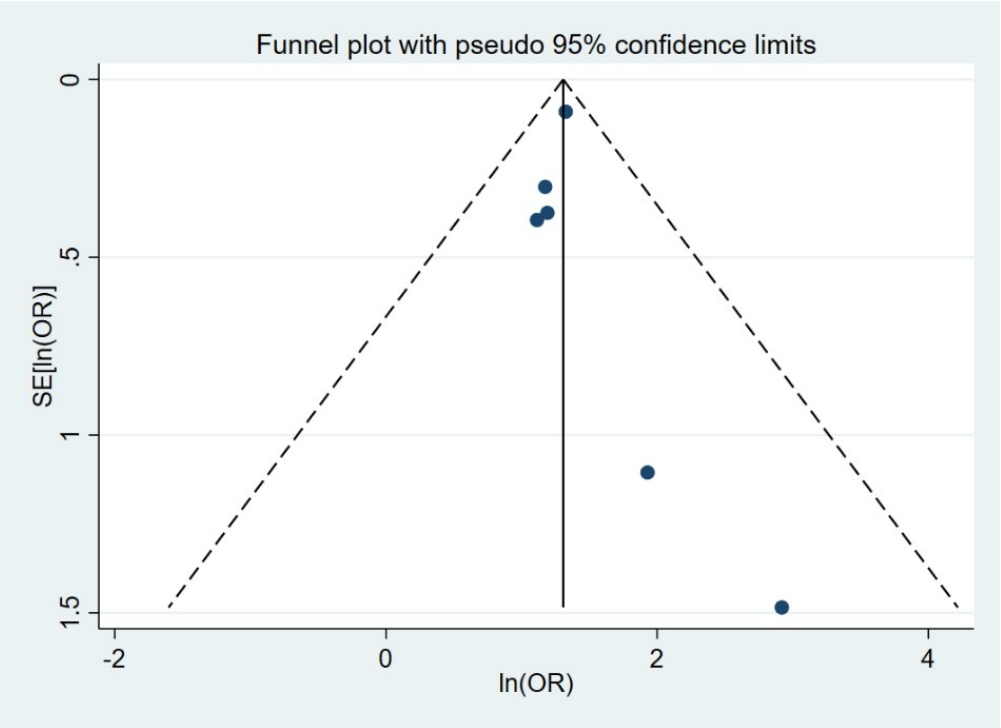

Funnel plot of log odds ratios for developing frozen shoulder in people with diabetes vs. those without diabetes.

109x79mm (220 x 220 DPI)

Appendix A

The following searches were originally conducted in December 2018 and updated in June 2021.

MEDLINE

Interface: OVID.

1. ((shoulder* or glenohumer* or subacromi* or acromi* or rotator cuff) adj3 (instability or bursitis or frozen or impinge* or tendonitis or tendinitis or pain* or osteoarthr* or periarthriti* or peri arthriti* or arthralgia)).ti,ab,kw.

2. Shoulder Impingement Syndrome/

3. exp Bursitis/

4. Rotator Cuff/

5. adhesive capsuliti*.ti,ab,kw.

6. Shoulder Pain/

7. or/1-6

8. exp Pain/

9. pain*.ti,ab,kw.

10. Arthralgia/

11. arthralgia.ti,ab,kw.

12. or/8-11

13. Shoulder/

14. Shoulder joint/

15. Acromioclavicular Joint/

16. (shoulder* or glenohumer* or subacromi* or acromi* or rotator cuff).ti,ab,kw.

17. or/13-16

18. 12 and 17

19. 7 or 18

20. exp Diabetes Mellitus/

21. diabet*.ti,ab,kw.

22. (DMi or DM i).ti,ab,kw.

23. (DM1 or DM 1).ti,ab,kw.

24. (DM2 or DM 2).ti,ab,kw.

25. (DMii or DM ii).ti,ab,kw.

26. (DM adj2 type).ti,ab,kw.

27. or/20-26

For peer review only

28. 19 and 27

29. exp animals/ not humans/

30. 28 not 29

EMBASE

Interface: OVID.

1. ((shoulder* or glenohumer* or subacromi* or acromi* or rotator cuff) adj3 (instability or bursitis or frozen or impinge* or tendonitis or tendinitis or pain* or osteoarthr* or periarthriti* or peri arthriti* or arthralgia)).ti,ab,kw.

2. exp shoulder impingement syndrome/

3. exp bursitis/

4. exp rotator cuff/

5. exp humeroscapular periarthritis/

6. adhesive capsuliti*.ti,ab,kw.

7. exp shoulder pain/

8. or/1-7

9. exp pain/

10. pain*.ti,ab,kw.

11. exp arthralgia/

12. arthralgia.ti,ab,kw.

13. or/9-12

14. exp shoulder/

15. Acromioclavicular Joint/

16. (shoulder* or glenohumer* or subacromi* or acromi* or rotator cuff).ti,ab,kw.

17. or/14-16

18. 13 and 17

19. 8 or 18

20. exp Diabetes Mellitus/

21. diabet*.ti,ab,kw.

22. (DMi or DM i).ti,ab,kw.

23. (DM1 or DM1).ti,ab,kw.

24. (DM2 or DM 2).ti,ab,kw.

25. (DMii or DM ii).ti,ab,kw.

For peer review only

26. (DM adj2 type).ti,ab,kw.

27. or/20-26

28. 19 and 27

29. exp animals/ not humans/

30. 28 not 29

31. limit 30 to embase

AMED

Interface: OVID.

1. ((shoulder* or glenohumer* or subacromi* or acromi* or rotator cuff) adj3 (instability or bursitis or frozen or impinge* or tendonitis or tendinitis or pain* or osteoarthr* or periarthriti* or peri arthriti* or arthralgia)).ti,ab.

2. exp Shoulder impingement syndrome/

3. exp Bursitis/

4. exp Rotator cuff/

5. adhesive capsuliti*.ti,ab.

6. exp shoulder pain/

7. or/1-6

8. exp Pain/

9. pain*.ti,ab.

10. exp Arthralgia/

11. arthralgia.ti,ab.

12. or/8-11

13. shoulder/

14. (shoulder* or glenohumer* or subacromi* or acromi* or rotator cuff).ti,ab.

15. or/13-14

16. 12 and 15

17. 7 or 16

18. exp Diabetes mellitus/

19. diabet*.ti,ab.

20. (DMi or DM i).ti,ab.

21. (DM1 or DM 1).ti,ab.

22. (DM2 or DM 2).ti,ab.

For peer review only

23. (DMii or DM ii).ti,ab.

24. (DM adj2 type).ti,ab.

25. or/18-24

26. 17 and 25

27. exp animals/ not humans/

28. 26 not 27

PsycINFO

Interface: OVID.

1. ((shoulder* or glenohumer* or subacromi* or acromi* or rotator cuff) adj3 (instability or bursitis or frozen or impinge* or tendonitis or tendinitis or pain* or osteoarthr* or periarthriti* or peri arthriti* or arthralgia)).ti,ab.

2. Shoulder Impingement Syndrome.ti,ab.

3. bursitis.ti,ab.

4. rotator cuff.ti,ab.

5. adhesive capsuliti*.ti,ab.

6. shoulder pain.ti,ab.

7. or/1-6

8. exp PAIN/

9. pain*.ti,ab.

10. arthralgia.ti,ab.

11. or/8-10

12. *“shoulder (anatomy)”/

13. shoulder*.ti,ab.

14. shoulder joint.ti,ab.

15. acromi*.ti,ab.

16. glenohumer*.ti,ab.

17. subacromi*.ti,ab.

18. or/12-17

19. 11 and 18

20. 7 or 19

21. exp DIABETES MELLITUS/

22. diabet*.ti,ab.

For peer review only

23. (DMi or DM i).ti,ab.

24. (DM1 or DM 1).ti,ab.

25. (DM2 or DM 2).ti,ab.
26. (DMii or DM ii).ti,ab.

27. (DM adj2 type).ti,ab.

28. or/21-27

29. 20 and 28

Web of Science

Science Citation Index Expanded and the Science Conference Proceedings Citation Index.

((

TS=(Shoulder* NEAR/3 instability) OR TS=(Shoulder* NEAR/3 bursitis) OR TS=(Shoulder*

NEAR/3 frozen) OR TS=(Shoulder* NEAR/3 impinge*) OR TS=(Shoulder* NEAR/3 tendonitis) OR TS=(Shoulder* NEAR/3 tendinitis) OR TS=(Shoulder* NEAR/3 pain) OR TS=(Shoulder*

NEAR/3 osteoarthr*) OR TS=(Shoulder* NEAR/3 periarthriti*) OR TS=(Shoulder* NEAR/3

“peri arthriti*”) OR TS=(Shoulder* NEAR/3 arthralgia)

OR

TS=(glenohumer* NEAR/3 instability) OR TS=(glenohumer* NEAR/3 bursitis) OR TS=(glenohumer*

NEAR/3 frozen) OR TS=(glenohumer* NEAR/3 impinge*) OR TS=(glenohumer* NEAR/3

tendonitis) OR TS=(glenohumer* NEAR/3 tendinitis) OR TS=(glenohumer* NEAR/3 pain)

OR TS=(glenohumer* NEAR/3 osteoarthr*) OR TS=(glenohumer* NEAR/3 periarthriti*) OR

TS=(glenohumer* NEAR/3 “peri arthriti*”) OR TS=(glenohumer* NEAR/3 arthralgia)

OR

TS=(subacromi* NEAR/3 instability) OR TS=(subacromi* NEAR/3 bursitis) OR TS=(subacromi*

NEAR/3 frozen) OR TS=(subacromi* NEAR/3 impinge*) OR TS=(subacromi* NEAR/3 tendonitis) OR TS=(subacromi* NEAR/3 tendinitis) OR TS=(subacromi* NEAR/3 pain) OR TS=(subacromi*

NEAR/3 osteoarthr*) OR TS=(subacromi* NEAR/3 periarthriti*) OR TS=(subacromi* NEAR/3

“peri arthriti*”) OR TS=(subacromi* NEAR/3 arthralgia)

OR

TS=(acromi* NEAR/3 instability) OR TS=(acromi* NEAR/3 bursitis) OR TS=(acromi* NEAR/3

frozen) OR TS=(acromi* NEAR/3 impinge*) OR TS=(acromi* NEAR/3 tendonitis) OR TS=(acromi*

NEAR/3 tendinitis) OR TS=(acromi* NEAR/3 pain) OR TS=(acromi* NEAR/3 osteoarthr*)

OR TS=(acromi* NEAR/3 periarthriti*) OR TS=(acromi* NEAR/3 “peri arthriti*”) OR TS=(acromi*

NEAR/3 arthralgia)

For peer review only

OR

TS=(“rotator cuff” NEAR/3 instability) OR TS=(“rotator cuff” NEAR/3 bursitis) OR TS=(“rotator cuff” NEAR/3 frozen) OR TS=(“rotator cuff” NEAR/3 impinge*) OR TS=(“rotator cuff” NEAR/3

tendonitis) OR TS=(“rotator cuff” NEAR/3 tendinitis) OR TS=(“rotator cuff” NEAR/3 pain)

OR TS=(“rotator cuff” NEAR/3 osteoarthr*) OR TS=(“rotator cuff” NEAR/3 periarthriti*) OR

TS=(“rotator cuff” NEAR/3 “peri arthriti*”) OR TS=(“rotator cuff” NEAR/3 arthralgia)

OR

TS=(“Rotator cuff”)

OR

TS=(“Adhesive capsuliti*”)

)

OR

TS=(arthralgia NEAR/3 shoulder* or arthralgia NEAR/3 glenohumer* or arthralgia NEAR/3

subacromi* or arthralgia NEAR/3 acromi* or arthralgia NEAR/3 “rotator cuff”)

OR TS=(pain* NEAR/3 shoulder* or pain* NEAR/3 glenohumer* or pain* NEAR/3 subacromi* or pain* NEAR/3 acromi* or pain* NEAR/3 “rotator cuff”)

)

And

TS=(diabet* or DM1 or “DM 1” or DM2 or “DM 2” or DMi or “DM i” or DMii or “DM ii” or

DM NEAR/2 type)

CINAHL

Interface: EBSCO. Filters: title or abstract

(

((shoulder* or glenohumer* or subacromi* or acromi* or “rotator cuff”) N3 (instability or bursitis or frozen or impinge* or tendonitis or tendinitis or pain* or osteoarthr* or periarthriti* or

“peri arthriti*” or arthralgia))

OR

(MH “Shoulder Impingement Syndrome”) OR (MH “Bursitis+”) OR (MH “Rotator Cuff+”) OR

(MH “Periarthritis”) OR (MH “Adhesive Capsulitis+”) OR (MH “Shoulder Pain”)

OR

((MH “Pain+”) or pain or (MH “Arthralgia+”) or arthralgia) and ((MH “Shoulder”) or (MH

“Acromioclavicular Joint”) or shoulder* or glenohumer* or subacromi* or acromi* or “rotator

cuff”)

For peer review only

)

AND

((MH “Diabetes Mellitus+”) or diabet* or (DMi or “DM i”) or (DM1 or “DM 1”) or (DMii or “DM ii”) or (DM2 or “DM 2”) or (DM N2 type))

Epistemonikos

Filters: title or abstract. Primary study. Not an RCT.

((“frozen shoulder” or “shoulder impinge*” or “shoulder bursitis” or “shoulder tendonitis” or “shoulder tendinitis” or “shoulder pain” or “pain in the shoulder” or “painful shoulder” or “shoulder osteoarthr*” or “shoulder joint arthr*” or “shoulder arthr”)

OR

(“glenohumeral impinge*” or “glenohumeral bursitis” or “glenohumeral tendonitis” or “glenohumeral tendinitis” or “glenohumeral pain” or “pain in the glenohumeral” or “glenohumeral osteoarthr*” or “glenohumeral arthr*” or “glenohumeral arthr”)

OR

(“subacromial impinge*” or “subacromial bursitis” or “subacromial tendonitis” or “subacromial tendinitis” or “subacromial pain” or “pain in the subacromial” or “subacromial osteoarthr*” or “subacromial arthr*” or “subacromial arthr”)

OR

“Rotator cuff”

OR

“periarthriti*”

OR

“peri arthriti*”

OR

“Adhesive capsuliti*”

)

AND

(diabet* or DM1 or DM2 or DMi or DMii or “type 1 DM” or “type 2 DM” or “type i DM” or “type ii DM”)

TRIP

For peer review only

(“frozen shoulder” or “shoulder pain” or “periathriti*” or “peri arthriti*” or “adhesive capsuliti*” or “shoulder impingement” or “bursitis” or “rotator cuff”) and “diabet*”

PEDro

Filters: body part = upper arm, shoulder or shoulder girdle

Title and abstract search: diabet*

Open Grey

Search 1: Diabet* and shoulder*

Search 2: Diabet* and glenohumer*

Search 3: Diabet* and subacromi*

Search 4: Diabet* and acromi*

Search 5: Diabet* and “rotator cuff*”

Search 6: Diabet* and bursitis

Search 7: Diabet* and periarthriti*

Search 8: Diabet* and “peri arthriti*”

Search 9: Diabet* and “adhesive capsuliti*”

Search 10: Diabet* and arthralgia

Grey literature report

Diabet*

For peer review only

Appendix B

Fig. B.1 Bar graph of QUIPS scores for each of the six bias domains: study participation, study attrition, diabetes/risk factor (RF) measurement, frozen shoulder/outcome measurement, study confounding, statistical analysis and reporting.

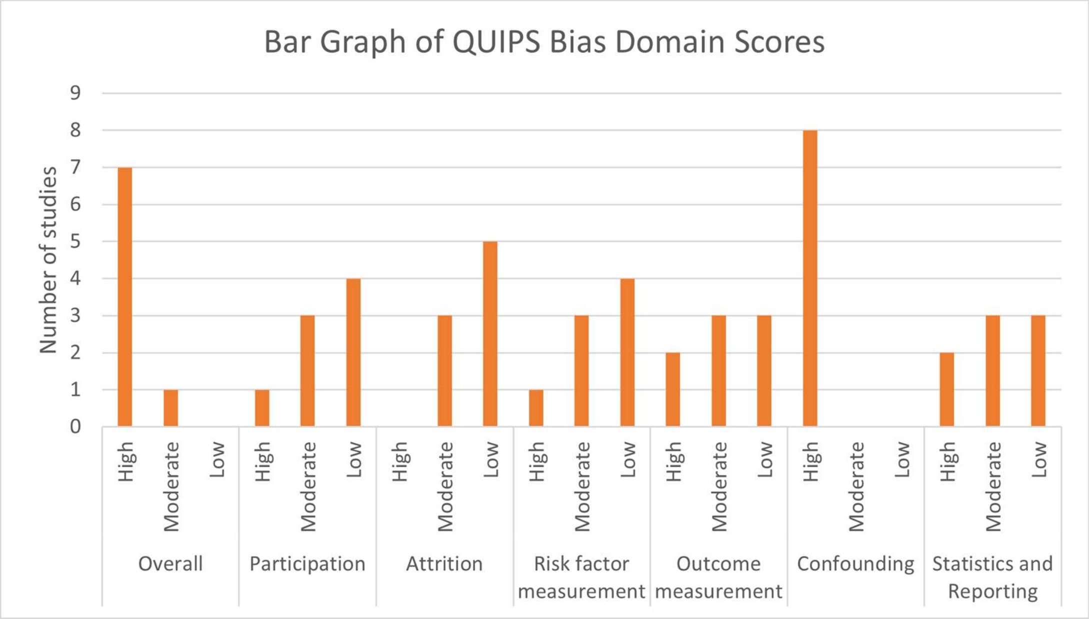

Appendix C

Table C.1 Raw data from each study.

**Case-Control Studies**				
Source	Number of cases	Number of controls	Number of cases with diabetes	Number of controls with diabetes
K. L. Boyle-Walker, et al., 1997 [32]	32	31	7	0
W. Li, et al., 2014 [33]	182	196	44	18
S-Y. Lee, et al., 2012 [34]	40	40	6	1
C. Milgrom, et al., 2008 [35]	126	98	37	11
K. Wang, et al., 2013 [36]	87	176	17	13
K. Kingston, et al., 2018 [37]	2190	2190	572	188
**Cohort studies**				
Source	Number of people with diabetes	Number of controls	Number of people with diabetes that developed frozen shoulder	Number of people without diabetes that developed frozen shoulder
Y-P. Huang, et al., 2013 [38]	78,827	236,481	946	2254
S-F. Lo, et al., 2013 [39]	5109	20,473	553	768

For peer review only

PRISMA 2020 for Abstracts Checklist

Section and Topic	Item #	Checklist item	Reported (Yes/No)
**TITLE**			
Title	1	Identify the report as a systematic review.	Y
**BACKGROUND**			
Objectives	2	Provide an explicit statement of the main objective(s) or question(s) the review addresses.	Y
**METHODS**			
Eligibility criteria	3	Specify the inclusion and exclusion criteria for the review.	Y
Information sources	4	Specify the information sources (e.g. databases, registers) used to identify studies and the date when each was last searched.	Y
Risk of bias	5	Specify the methods used to assess risk of bias in the included studies.	Y
Synthesis of results	6	Specify the methods used to present and synthesise results.	Y
**RESULTS**			
Included studies	7	Give the total number of included studies and participants and summarise relevant characteristics of studies.	Y
Synthesis of results	8	Present results for main outcomes, preferably indicating the number of included studies and participants for each. If meta-analysis was done, report the summary estimate and confidence/credible interval. If comparing groups, indicate the direction of the effect (i.e. which group is favoured).	Y
**DISCUSSION**			
Limitations of evidence	9	Provide a brief summary of the limitations of the evidence included in the review (e.g. study risk of bias, inconsistency and imprecision).	Y
Interpretation	10	Provide a general interpretation of the results and important implications.	Y
**OTHER**			
Funding	11	Specify the primary source of funding for the review.	Y
Registration	12	Provide the register name and registration number.	Y

From: Page MJ, McKenzie JE, Bossuyt PM, Boutron I, Hoffmann TC, Mulrow CD, et al. The PRISMA 2020 statement: an updated guideline for reporting systematic reviews. BMJ 2021;372:n71. doi: 10.1136/bmj.n71

For more information, visit: http://www.prisma-statement.org/

For peer review only - http://bmjopen.bmj.com/site/about/guidelines.xhtml

PRISMA 2020 Checklist

Section and Topic	Item #	Checklist item	Location where item is reported
**TITLE**			
Title	1	Identify the report as a systematic review.	Title
**ABSTRACT**			
Abstract	2	See the PRISMA 2020 for Abstracts checklist.	Abstract checklist attached
**INTRODUCTION**			
Rationale	3	Describe the rationale for the review in the context of existing knowledge.	Introduction paragraph 4
Objectives	4	Provide an explicit statement of the objective(s) or question(s) the review addresses.	Introduction paragraph 4
**METHODS**			
Eligibility criteria	5	Specify the inclusion and exclusion criteria for the review and how studies were grouped for the syntheses.	Section 2.3
Information sources	6	Specify all databases, registers, websites, organisations, reference lists and other sources searched or consulted to identify studies. Specify the date when each source was last searched or consulted.	Section 2.1
Search strategy	7	Present the full search strategies for all databases, registers and websites, including any filters and limits used.	Appendix A
Selection process	8	Specify the methods used to decide whether a study met the inclusion criteria of the review, including how many reviewers screened each record and each report retrieved, whether they worked independently, and if applicable, details of automation tools used in the process.	Section 2.2
Data collection process	9	Specify the methods used to collect data from reports, including how many reviewers collected data from each report, whether they worked independently, any processes for obtaining or confirming data from study investigators, and if applicable, details of automation tools used in the process.	Section 2.4
Data items	10a	List and define all outcomes for which data were sought. Specify whether all results that were compatible with each outcome domain in each study were sought (e.g. for all measures, time points, analyses), and if not, the methods used to decide which results to collect.	Section 2.3, 2.4
	10b	List and define all other variables for which data were sought (e.g. participant and intervention characteristics, funding sources). Describe any assumptions made about any missing or unclear information.	Section 2.4
Study risk of bias assessment	11	Specify the methods used to assess risk of bias in the included studies, including details of the tool(s) used, how many reviewers assessed each study and whether they worked independently, and if applicable, details of automation tools used in the process.	Section 2.4
Effect measures	12	Specify for each outcome the effect measure(s) (e.g. risk ratio, mean difference) used in the synthesis or presentation of results.	Section 2.5
Synthesis methods	13a	Describe the processes used to decide which studies were eligible for each synthesis (e.g. tabulating the study intervention characteristics and comparing against the planned groups for each synthesis (item #5)).	Section 2.5 lines 1-3.
	13b	Describe any methods required to prepare the data for presentation or synthesis, such as handling of missing summary statistics, or data conversions.	Section 2.5 lines 4-6
	13c	Describe any methods used to tabulate or visually display results of individual studies and syntheses.	Section 2.5 line 12
	13d	Describe any methods used to synthesize results and provide a rationale for the choice(s). If meta-analysis was performed, describe the model(s), method(s) to identify the presence and extent of statistical heterogeneity, and software package(s) used.	Section 2.5
	13e	Describe any methods used to explore possible causes of heterogeneity among study results (e.g. subgroup analysis, meta-regression).	n/a Not enough

For peer review only

PRISMA 2020 Checklist

Section and Topic	Item #	Checklist item	Location where item is reported
			studies present to do this
	13f	Describe any sensitivity analyses conducted to assess robustness of the synthesized results.	Section 2.5 lines 14-16
Reporting bias assessment	14	Describe any methods used to assess risk of bias due to missing results in a synthesis (arising from reporting biases).	n/a no missing results
Certainty assessment	15	Describe any methods used to assess certainty (or confidence) in the body of evidence for an outcome.	n/a
**RESULTS**			
Study selection	16a	Describe the results of the search and selection process, from the number of records identified in the search to the number of studies included in the review, ideally using a flow diagram.	Section 1 paragraph 1, Figure 1
	16b	Cite studies that might appear to meet the inclusion criteria, but which were excluded, and explain why they were excluded.	Figure 1
Study characteristics	17	Cite each included study and present its characteristics.	Table 1
Risk of bias in studies	18	Present assessments of risk of bias for each included study.	Table 2
Results of individual studies	19	For all outcomes, present, for each study: (a) summary statistics for each group (where appropriate) and (b) an effect estimate and its precision (e.g. confidence/credible interval), ideally using structured tables or plots.	Figure 2
Results of syntheses	20a	For each synthesis, briefly summarise the characteristics and risk of bias among contributing studies.	Table 1
	20b	Present results of all statistical syntheses conducted. If meta-analysis was done, present for each the summary estimate and its precision (e.g. confidence/credible interval) and measures of statistical heterogeneity. If comparing groups, describe the direction of the effect.	Section 3 paragraph 4
	20c	Present results of all investigations of possible causes of heterogeneity among study results.	n/a not enough studies to investigate causes of heterogeneity
	20d	Present results of all sensitivity analyses conducted to assess the robustness of the synthesized results.	Section 3 paragraph 4, Figure 3
Reporting biases	21	Present assessments of risk of bias due to missing results (arising from reporting biases) for each synthesis assessed.	n/a no missing results
Certainty of evidence	22	Present assessments of certainty (or confidence) in the body of evidence for each outcome assessed.	n/a
**DISCUSSION**			

For peer review only

PRISMA 2020 Checklist

Section and Topic	Item #	Checklist item	Location where item is reported
Discussion	23a	Provide a general interpretation of the results in the context of other evidence.	Section 4, paragraphs 1-3
	23b	Discuss any limitations of the evidence included in the review.	Section 4, paragraphs 4-6
	23c	Discuss any limitations of the review processes used.	n/a we did not come across any limitations of the review process
	23d	Discuss implications of the results for practice, policy, and future research.	Section 4, paragraphs 7-9, Section 5.
**OTHER INFORMATION**			
Registration and protocol	24a	Provide registration information for the review, including register name and registration number, or state that the review was not registered.	PROSPERO registration number is included in abstract
	24b	Indicate where the review protocol can be accessed, or state that a protocol was not prepared.	PROSPERO registration number is included in abstract
	24c	Describe and explain any amendments to information provided at registration or in the protocol.	PROSPERO registration number is included in abstract
Support	25	Describe sources of financial or non-financial support for the review, and the role of the funders or sponsors in the review.	Funding section
Competing interests	26	Declare any competing interests of review authors.	Conflicts of interest statement
Availability of data, code and other materials	27	Report which of the following are publicly available and where they can be found: template data collection forms; data extracted from included studies; data used for all analyses; analytic code; any other materials used in the review.	Appendix B, C

For peer review only

PRISMA 2020 Checklist

From: Page MJ, McKenzie JE, Bossuyt PM, Boutron I, Hoffmann TC, Mulrow CD, et al. The PRISMA 2020 statement: an updated guideline for reporting systematic reviews. BMJ 2021;372:n71. doi: 10.1136/bmj.n71

For more information, visit: http://www.prisma-statement.org/

For peer review only
